# Photo-uncaging of a ferrocene-bridged dinuclear iridium(iii) complex for three-photon photoimmunotherapy against hypoxic melanoma†

**DOI:** 10.1039/d5sc04006j

**Published:** 2025-07-10

**Authors:** Lina Xie, Zhuoli Chen, Tianying Wang, Jinzhe Liang, Qiaoshan Lie, Chengzhi Jin, Xiting Zhang, Yu Chen, Hui Chao

**Affiliations:** a MOE Key Laboratory of Bioinorganic and Synthetic Chemistry, State Key Laboratory of Anti-Infective Drug Discovery and Development, Guangdong Basic Research Center of Excellence for Functional Molecular Engineering, School of Chemistry, Sun Yat-Sen University Guangzhou 510006 P. R. China chenyu63@mail.sysu.edu.cn ceschh@mail.sysu.edu.cn; b Huangpu Hydrogen Innovation Center, Guangzhou Key Laboratory for Clean Energy and Materials, School of Chemistry and Chemical Engineering, Guangzhou University Guangzhou 510006 P. R. China zhxt@gzhu.edu.cn; c MOE Key Laboratory of Theoretical Organic Chemistry and Functional Molecule, School of Chemistry and Chemical Engineering, Hunan University of Science and Technology Xiangtan 400201 P. R. China

## Abstract

The complexity of solid tumors in terms of light scattering, oxygen insufficiency, and redox imbalance complicates the strategic design of photoactivated therapy. In this work, an unprecedented photoactivated homolysis process of ferrocene is driven by the photochemistry of a conjugated cyclometalated iridium(iii) complex upon 970 nm three-photon excitation, exhibiting photo-uncaging, biocatalysis, and an ROS storm all in one moiety. Trapping assays, ultrafast spectroscopy, and DFT calculations reveal the release of Fe^2+^ ions, the location of carbon-centered radicals, and the essential single electron transfer (SET) process for their generation. Such a photo-uncaging pattern harnesses peripheral substrates (O_2_, H_2_O_2,_ and H_2_O) for ROS generation. It continuously degrades the biomolecule homeostasis (GSH and NADH), inducing high immunogenic ferroptosis and necroptosis in hypoxic melanoma models for long-term photoimmunotherapy. The uncaging of the photostable ferrocene by transition metal photochemistry develops an elegant paradigm for multi-functional molecular photoactivated therapy.

## Abstract 2

A novel ferrocene-bridged dinuclear Ir(iii) photoactivated prodrug was found to photo-uncage the ferrocene upon three-photon excitation up to 970 nm to induce ferroptosis and necroptosis to evoke systemic anticancer immunity.

## Graphical abstract

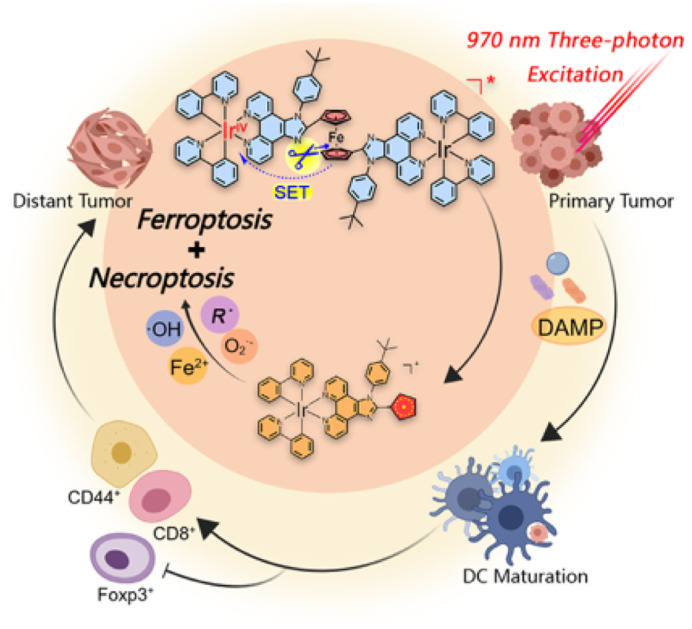

## Introduction

The flourishing application of photodynamic therapy in the clinical treatment of skin-related disorders and endoscope-accessible solid tumors encourages the development of novel photosensitizers and superior photo-triggered therapeutic strategies.^1^ Research on oxygen-independent type I photosensitizers has been thriving to conquer the oxygen limitation in solid tumors.^2–5^ However, the competition among diverse decay processes and the dilemma between electron transfer efficiency and excited redox potentials stave off further advancements. Photoactivated prodrug strategies, first aiming to optimize the biosafety and accuracy of PDT, show great potential in multi-module therapy for enhanced photocytotoxicity, in which the photo-responsive cage silences the druggable moiety or acts as a cage for the photosensitizer.^6,7^ Specifically, metal complexes could be a powerful photoactivated platform owing to their unique excited states and flexible structure expandability. It has been reported that Pt(iv),^8–10^ Au(iii),^11–13^ Ru(ii)^14–16^ and Ir(iii)^17–19^ complexes could be photoactivated in response to the gradient of oxygen in the tumor, in which intramolecular photoinduced electron transfer (PET) processes or photoredox reactions with biomolecules are the prevalent strategies to combat the severely hypoxic TME with an O_2_ content of 0.02–2%.^20^

Conventional molecular design of multi-module photoactivated prodrugs relies on aggregating multiple functional modules. Structural redundancy emerges as a primary concern due to the functional oversimplification of individual modules, where limited cross-module synergy compromises design efficiency.^21^ This issue is compounded by fixed stoichiometric ratios of chemotherapeutic modules that require elevated concentrations to achieve therapeutic efficacy. Furthermore, conventional photoactivated linkers demand high-energy photons within the UV/visible spectrum range, while photocatalytic activation systems exhibit stringent substrate specificity.^22^ These chemical and photophysical limitations restrict *in vivo* applicability and exacerbate the complexity of optimizing the performance parameters of prodrugs. Accordingly, recent advancements in optimizing photoactivated prodrugs focus on (1) multicomponent molecular assembly platforms for flexible and universal prodrug and photocatalyst design;^23–27^ (2) highly reactive oxygen-independent radical generation for broader substrate adaptability;^17,28–32^ (3) catalysis-based photoactivated systems for long-lasting therapeutic effects^10,33–37^ and (4) photoinduced intermolecular electron/energy transfer for lower photon energy (*i.e.* red light) prodrug activation.^38–40^

Oxidative stress induced by phototherapy is reportedly proinflammatory, and recent studies have focused on stimulating a systemic anticancer immune response through phototherapy, aiming at the reinforcement of metastatic inhibition.^41,42^ Cancer cells undergoing apoptosis suffer from the internalization and degradation of damage-associated molecular patterns (DAMPs), leading to poor immunogenicity. Non-apoptotic programmed cell death pathways shed light on photo-immunotherapy by delicately tuning the reactive oxygen species (ROS) destination to trigger the acute damage of specific organelles or plasma membranes, consequently accompanied by the release of DAMPs.^43–46^

**Scheme 1:**
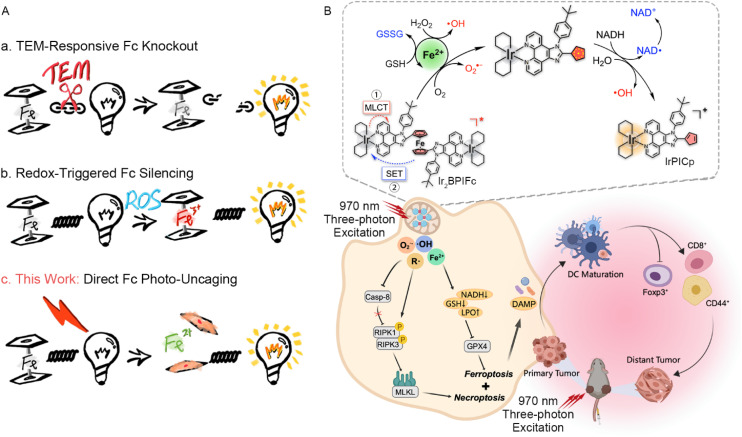
(A) Comparison between the traditional ferrocene (Fc) prodrug activation and the direct Fc photo-uncaging strategy in this work. (B) Schematic illustration of the chemical and biological mechanism of action of Ir_2_BPIFc for three-photon photoimmunotherapy.

The robust stability of ferrocene (Fc) in air and aqueous media, as well as in chemical environments allows its utilization and derivatization in medicinal chemistry.^47,48^ Capitalizing on the reversible redox properties of Fc, the modification of clinical drugs with Fc endows them with Fenton-like hydroxyl radical generating efficacy, driving clinical trials of ferroquine^49,50^ and fueling the development of Fc-derived chemodynamic therapy (CDT).^51–54^ Ferrocenyl prodrugs with such patterns rely on the recognition of the specific tumor microenvironment (TME), bringing the challenge of balancing activation efficiency and specificity. In phototherapy, Fc is used as an antenna in the PET process, generally causing emission quenching and photocatalysis.^55–57^ The action mechanism of ferrocenyl prodrugs focuses on the conditional knockout or silencing of ferrocene, rather than direct photoactivation (Scheme 1A).^58–60^

Earlier studies revealed the photo-liability of benzoyl-ferrocene, during which a cyclopentadienyl anion and free Fe^3+^ ion were generated.^61^ The photolysis of a ferrocenyl–BODIPY conjugate in water discovered by Chen *et al.* brings a new horizon to the design of ferrocenyl prodrugs, but advanced studies remain stagnant.^62^ Recent studies that utilized ferrocene as a photo-responsive group also capture Fe^3+^ ions as the primary product.^63^ By imposing molecular strain on the ferrocene, the excited triplet state of a Fc-nanohoop became accessible for external attack to release Fe^2+^ ions.^64^ In this article, a novel ferrocene-bridged dinuclear cyclometalated iridium(iii) complex Ir_2_BPIFc is designed and synthesized, with its emission and intrinsic cytotoxicity markedly silenced. Upon excitation with 405 nm LED light or a femtosecond laser up to 970 nm, the ferrocene moiety is found to dissociate to generate mononuclear complex IrPICp and free Fe^2+^ ions, indicating an unprecedent homolysis behavior (Scheme 1B). Detailed investigations show that the photolysis of Ir_2_BPIFc generates carbon-centered radicals that oxidize the peripheral water molecules or biomolecules. Various trapping assays confirmed the presence of photoinduced carbon-centered radicals through high-resolution mass spectrometry (HRMS) and electron spin resonance (EPR) spectroscopy. The ultrafast spectroscopy and DFT simulation reveal that the robust metal-to-ligand charge transfer (MLCT) in the iridium moiety and the SET from ferrocene to the excited iridium moiety are the keys to the carbon-centered radical generation.

It is worth highlighting that the flexible photoredox properties of the iridium(iii) complex reshape ferrocene, a moiety formerly considered stable, to be a multi-functional photoactivated cage with catalytic and redox-potential-independent phototherapeutic performance. In a simulated hypoxic environment (2% O_2_), the abundant generation of multiple types of ROS through (1) the photo-generated carbon-centered radicals from ferrocene, (2) the Fenton reaction by the released Fe^2+^ and (3) the photo-uncaged type I PDT, along with the disruption of key redox biomolecules by (1) the redox shuttling of iron ions and (2) direct attack from photo-generated carbon-centered radicals, triggers the ferroptosis and necroptosis of cancer cells. This universal redox attack, initiated by the iridium-enabled photo-uncaging of ferrocene, successfully demonstrates potent inhibition of primary tumors and compelling activation of systemic anticancer immunity for long-term distant tumor inhibition.

## Results and discussion

Harnessing the robust chemical stability of ferrocene, the ligand BPIFc was synthesized by the imidazole condensation of 1,1′-ferrocene-dicarboxaldehyde, 1,10-phenanthroline-5,6-dione and 4-*tert*-butylaniline (Scheme S1†). After refluxing with [Ir(ppy)_2_Cl]_2_, the resultant dinuclear complex Ir_2_BPIFc, but not BPIFc, was found to need protection from light when purified by column chromatography. This suggests that the coordination into the cyclometalated Ir(iii) complex drastically changes the photophysical properties of BPIFc, inspiring us to pay attention to the photo-responsive performance of Ir_2_BPIFc. All of the synthesized products were characterized using ^1^H NMR, ^13^C NMR, and HRMS (see ESI, Fig. S1–S8†). The purity of Ir_2_BPIFc (99.09%) is adequate for biological analyses (Fig. S9†). Specifically, the stability of Ir_2_BPIFc in the dark in a redox (H_2_O_2_ or GSH) environment and *in vitro* cultured A735 cells was analyzed by HPLC, resulting in at least 93.67% Ir_2_BPIFc remaining intact after 24 h of incubation (Fig. S10†). The robustness of Ir_2_BPIFc in various active environments meets the application's needs for photoactivated prodrugs.

### Photo-uncaging of Ir_2_BPIFc to generate ROS

**Fig. 1:**
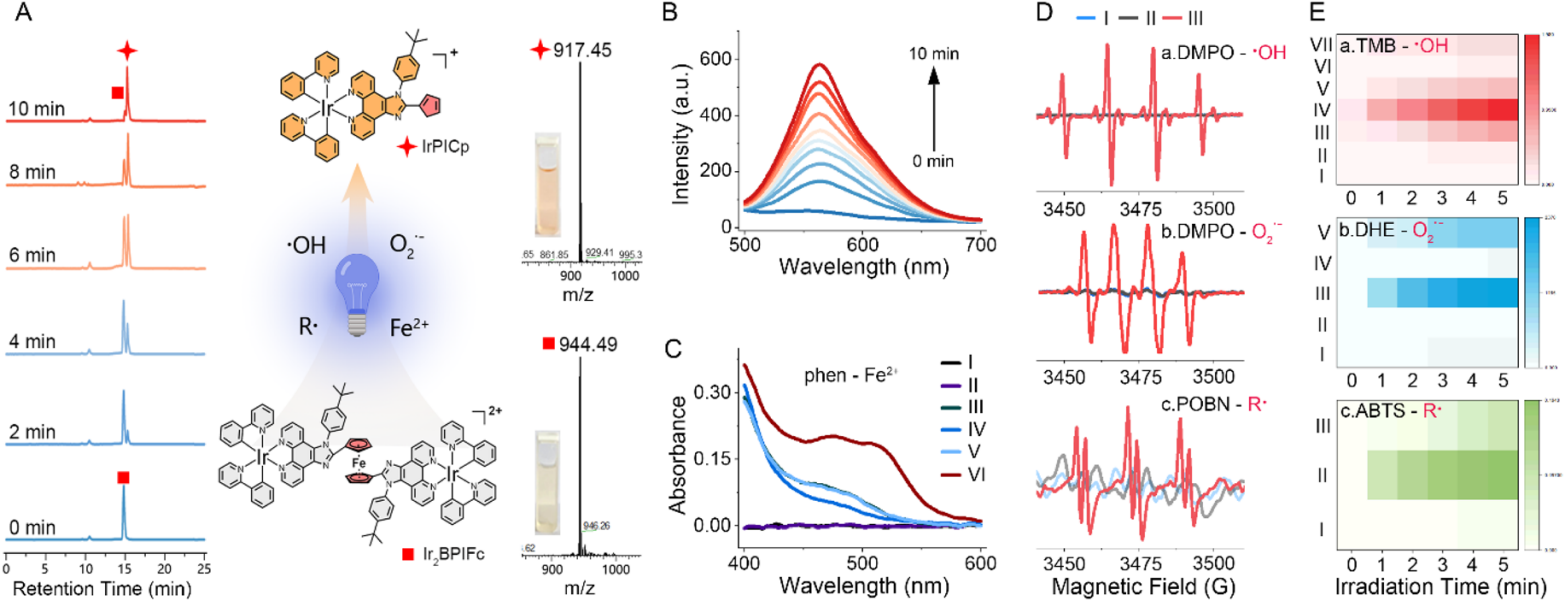
(A) Illustration of the key discovery of the photolysis processes of Ir_2_BPIFc in hypoxia upon irradiation; (left panel) HPLC monitoring of the photolysis of Ir_2_BPIFc; (right panel) ESI-MS analysis of the photo-reaction solution at 0 min and 10 min. (B) The emission recovery by the photolysis of Ir_2_BPIFc in hypoxia upon irradiation. (C) Detection of the released Fe^2+^ from Ir_2_BPIFc by phenanthroline (phen, 1 mM) upon irradiation in hypoxia; (I) phen + dark; (II) phen + light; (III) Ir_2_BPIFc + dark; (IV) Ir_2_BPIFc + light; (V) Ir_2_BPIFc + phen + dark; (VI) Ir_2_BPIFc + phen + light. (D) EPR examination of the generation of various radicals in hypoxia upon irradiation: (I) trapping agent + light; (II) Ir_2_BPIFc + dark; (III) Ir_2_BPIFc + light. (E) (a) TMB as the probe to detect the ˙OH generation of Ir_2_BPIFc and IrPICp (20 μM): (I) TMB + light; (II) Ir_2_BPIFc + dark; (III) Ir_2_BPIFc + light; (IV) Ir_2_BPIFc + H_2_O_2_ (100 μM) + light; (V) Ir_2_BPIFc + phen (1 mM) + H_2_O_2_ + light; (VI) IrPICp + dark; (VII) IrPICp + light; (b) DHE as the probe to detect the O_2_˙^−^ generation of Ir_2_BPIFc and IrPICp (20 μM): (I) DHE + light; (II) Ir_2_BPIFc + dark; (III) Ir_2_BPIFc + light; (IV) IrPICp + dark; (V) IrPICp + light; (c) ABTS as the probe to detect the organic radical generation of Ir_2_BPIFc: (I) dioxane + light; (II) Ir_2_BPIFc + dark; (III) Ir_2_BPIFc + light. Concentration of Ir_2_BPIFc: 10 μM; irradiation parameter: 405 nm, 20 mW cm^−2^, and 10 min.

The ferrocene-bridged Ir_2_BPIFc showed similar absorption spectra to those reported for 2-phenylpyridine cyclometalated Ir(iii) complexes, with an additional peak at around 470 nm attributed to the ferrocene moiety. The ferrocene bridge markedly quenched the emission of Ir_2_BPIFc. To our surprise, under continuous irradiation by 405 nm LED light, the emission at ∼565 nm gradually recovered (*k* = 0.26 ± 0.03 min^−1^) (Fig. 1B). The common TME-enriched substances did not significantly affect the photolysis efficiency (Fig. S11†). Detailed estimations first revealed that an absorption hypochromicity (*k* = 0.164 ± 0.007 min^−1^) occurred at ∼470 nm in Ir_2_BPIFc, which could not be observed in ligand BPIFc (Fig. S13†). Meanwhile, after 10 min irradiation, a new species was captured (Fig. 1A), which was assigned to the mononuclear IrPICp by HRMS (*m*/*z* = 917.29382) and ^1^H NMR analysis (Fig. S15 and S16†) after purification. Combined with the successful capture of Fe^2+^ by 1,10-phenanthroline (phen) in solution upon 405 nm irradiation (Fig. 1C and S12†), it is believed that Ir_2_BPIFc undergoes photolysis on the ferrocene moiety upon light irradiation.

Given the photoactivated anticancer performance expectation, the ROS generation during the photolysis of Ir_2_BPIFc in hypoxia became the focus of further investigations. Screening radical species by the electron paramagnetic resonance (EPR) technique, the efficient generation of singlet oxygen (^1^O_2_) in normoxia upon irradiation was proved by 2,2,6,6-tetramethylpiperidine (TEMP) capture, indicating the potential of Ir_2_BPIFc to be a standard type II photosensitizer (Fig. S17A†). When employing 5,5-dimethyl-1-pyrroline *N*-oxide (DMPO) as the trapping agent in degassed water, the captured quartet peak with a 1 : 2 : 2 : 1 pattern upon irradiation could be assigned to the hydroxyl radical (˙OH) adduct, and the sextet peak was reported to be the carbon radical adduct (Fig. 1D, upper graph).^65,66^ Altering the solvent to dry acetonitrile, the ˙OH signals in EPR could not be captured (Fig. S16B†), suggesting the involvement of water molecules in the electron transfer generation of ˙OH. A typical superoxide anion (O_2_˙^−^) signal was also captured by DMPO in DMSO upon irradiation, suggesting the involvement of another electron transfer process during the photolysis (Fig. 1D, middle graph). *N-tert*-Butyl-α-(4-pyridyl-1-oxide) nitrone (POBN) was employed in degassed dioxane for another EPR estimation. The typical sextet signals (*α*_N_ = 15.08 G, *α*_β-H_ = 2.34 G) of the POBN adduct confirmed the generation of organic radicals (R˙) during the photolysis of Ir_2_BPIFc (Fig. 1D, lower graph).^67^

The generation of ˙OH, O_2_˙^−^ and R˙ was also semi-quantified in solution. In the presence of H_2_O_2_, the ˙OH generation efficiency evidently increased (Fig. 1E(a), group IV) compared to the standard irradiation (Fig. 1E(a), group III), while the introduction of the captured ligand phenanthroline (phen) markedly restricted the ˙OH generation to the standard conditions, indicating the Fenton effect from the released Fe^2+^ ions. Encouragingly, the released IrPICp was found to be a type I photosensitizer that moderately generated ˙OH (Fig. 1E(a), group VII). It is worth mentioning that the ˙OH generation of Ir_2_BPIFc (10 μM) was ∼3.5 fold higher than that of IrPICp (20 μM) with the same amount of substance of the iridium moiety, suggesting that the ˙OH generated during the photo-uncaging of Ir_2_BPIFc was derived from the photolysis process of Ir_2_BPIFc besides the type I PDT of IrPICp (Fig. S18†). The O_2_˙^−^ generation detected by dihydroethidium (DHE) also indicated the multiple origins of O_2_˙^−^ besides the type I PDT of IrPICp, as the O_2_˙^−^ generation of Ir_2_BPIFc (Fig. 1E(b), group III) was ∼1.9 fold higher than that of IrPICp (Fig. 1E(b), group V). When employing 2,2′-azino-bis(3-ethylbenzothiazoline-6-sulfonic acid) (ABTS) as the organic radical probe, the characteristic absorption changes of ABTS at 400 nm and 750 nm again proved the presence of organic radicals during the photo-uncaging of Ir_2_BPIFc (Fig. 1E(c) and S20†). Such multi-module ROS burst improved the feasibility of the phototherapy of Ir_2_BPIFc, and it was all under the control of the photo-uncaging of Ir_2_BPIFc, showing great potential in developing on-demand anticancer phototherapy.

### SET dominates the photo-uncaging of Ir_2_BPIFc

**Fig. 2:**
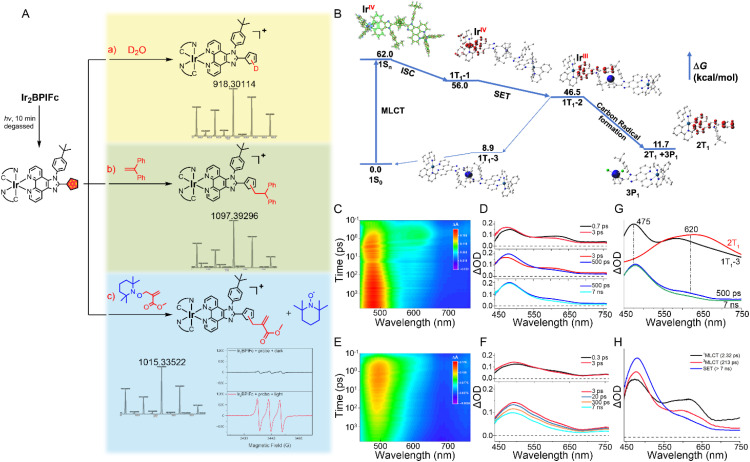
(A) Schematic illustration of the key strategies to identify the photo-uncaging carbon-centered radical generation in Ir_2_BPIFc (10 μM) upon irradiation at 405 nm (20 mW cm^−2^); (a) HRMS of Ir_2_BPIFc in acetonitrile/D_2_O (1/1, v/v); (b) HRMS of Ir_2_BPIFc with diphenylethene (10 μM); (c) HRMS of Ir_2_BPIFc with a synthesized probe (10 μM) and the EPR signal of the released TEMPO. (B) Potential energy surface profiles for Ir_2_BPIFc in H_2_O at the B3LYP/SDD(Ir, Fe)-6-31G*(H_2_O) level of theory; blue surface: hole, green surface: electron, red surface: α spin, blue surface: β spin. (C) Femtosecond transient absorption (fs-TA) spectra of Ir_2_BPIFc in DMF (*λ*_ex_ = 390 nm). (D) Evolution of the fs-TA spectra of Ir_2_BPIFc at different times. (E) Femtosecond transient absorption (fsTA) spectra of IrPICp in DMF (*λ*_ex_ = 390 nm). (F) Evolution of the fs-TA spectra of Ir_2_BPIFc at different times. (G) The calculated spectra of Ir_2_BPIFc at specific excited states obtained by TD-B3LYP/SDD(Ir, Fe)-6-31G*(H_2_O). (H) Species-associated spectra (SAS) of Ir_2_BPIFc; the fitting lifetimes are labelled in the legend.

The above phenomena prompted us to locate the organic radicals and further reveal the complete photolysis processes. Employing two organic radical traps during the photo-uncaging process successfully generated the corresponding IrPICp adducts (Fig. 2A) and the reporting TEMPO EPR signal (Fig. S21 and S22†),^68^ proving the generation of organic radicals on the photolytic intermediates. Altering the reaction environment to deuterium oxide, HRMS captured signals assigned to the single-deuterated IrPICp (*m*/*z* = 918.30114, Fig. S23†), and the ^1^H NMR spectra showed distinct shifts on the signals assigned to the cyclopentadiene after deuteration (Fig. S24†), indicating the possible localization of the carbon-centered radical on cyclopentadiene and verifying the water molecule attack on the radical site. The homolysis of the ferrocene moiety and the release of ferrous ions suggested a single electron transfer (SET) process from ferrocene to generate cyclopentadienyl radicals. According to the Rehm–Weller theory based on cyclic voltammetry analysis and the optical spectra,^69,70^ the Ir(iii) moiety in Ir_2_BPIFc could be an oxidant in the excited state (*E*(M*/M^−^) = 1.022 V *vs.* SCE) that undergoes single electron transfer (SET) from Fc to Ir(iii) upon light excitation (Fig. S25†), and a reductant (*E*(M^+^/M*) = −1.224 V *vs.* SCE) toward O_2_
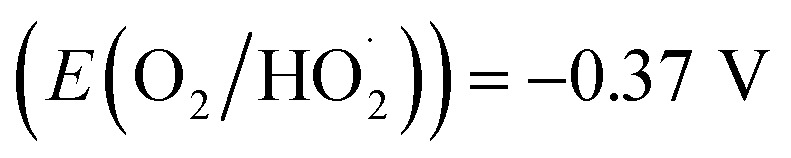
 to complete the redox cycle.

DFT calculation further demonstrated the potential energy surface profiles of Ir_2_BPIFc in different excited states (Fig. 2B). Undergoing efficient MLCT and ISC, the molecule in the 1T_1_-1 state could theoretically go through an SET process to generate separated α spins on the dipyridyl ligand and cyclopentadienyl group and β spins on Fe(ii). Once the decay inclination was overcome, a thermally steady state composed of cyclopentadienyl radicals could be generated (2T_1_). Femtosecond transient absorption spectroscopy was then applied to differentiate the excited state species (Fig. 2C and E). Upon excitation at 380 nm, Ir_2_BPIFc and IrPICp simultaneously exhibited hyperchromicity at 470 to 490 nm and hypochromicity at 620 to 630 nm in a similar lifetime (<3 ps), resulting in an isosbestic point that suggests the transformation of excited state species (Fig. 2D and F). Considering the iridium core's heavy atom effect, these signal kinetics could be ascribed to the intersystem crossing (ISC) process. Unlike the global decay of the signals of IrPICp after 3 ps (Fig. 2F), the spectra of Ir_2_BPIFc from 3 ps to 500 ps exhibited a bathochromic and hyperchromic change at ∼480 nm, demonstrating another isosbestic point. After 500 ps, the signals of Ir_2_BPIFc contained two kinds of excited state species that displayed different decay dynamics. Combing the partial spectral simulation of the potential energy surface profiles of Ir_2_BPIFc at different excited states (Fig. 2G) and the reported efficient SET to excited Ir(iii) complexes,^71^ it could be ascribed to the fast SET process that generates the mix of lower triplet states (*i.e.*, 1T_1_-3) and the photolytic radical-contained species (*i.e.*, 2T_1_). Global analysis of fs-TA data by Glotaran software successfully isolated three species-associated spectra that fitted the experimental signals and dynamics (Fig. 2H). Combined, it could be proposed that the SET process dominates the photo-uncaging of Ir_2_BPIFc, leading to the generation of a radical species with a relatively long lifetime to act as a potential anticancer prodrug.

### Synergistic cell death through the photo-uncaging of Ir_2_BPIFc

**Fig. 3:**
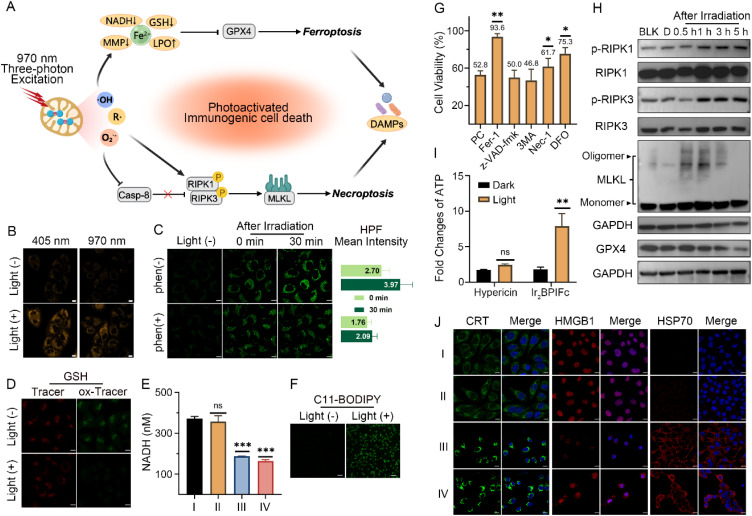
(A) Schematic illustration of the photo-uncaging immunotherapeutic effects of Ir_2_BPIFc*in vitro*. (B) The emission recovery by the photolysis of Ir_2_BPIFc in A375 cells upon irradiation at 405 nm (1 PE) or 970 nm (3 PE), scale bar: 10 μm. (C) ˙OH generation of the photo-uncaging of Ir_2_BPIFc in A375 cells upon irradiation at 970 (3 PE) in hypoxia, with phen (100 μM) as the Fe^2+^ scavenger, scale bar: 10 μm. (D) Intracellular GSH changes induced by the photo-uncaging release of Fe^2+^ from Ir_2_BPIFc in A375 cells upon irradiation with 970 nm (3 PE) in hypoxia, GSHTracer: *λ*_ex/em_ = 514/580 ± 10 nm, ox-GSHTracer: *λ*_ex/em_ = 488/525 ± 10 nm, scale bar: 10 μm. (E) Intracellular NADH concentration changes induced by the photo-uncaging of Ir_2_BPIFc in A375 cells upon irradiation with 970 nm (3 PE) in hypoxia. (I) Control, (II) Ir_2_BPIFc + dark; (III) Ir_2_BPIFc + light in hypoxia, (IV) Ir_2_BPIFc + light in normoxia. (F) Lipid peroxidation induced by the photo-uncaging of Ir_2_BPIFc in A375 cells upon irradiation with 970 nm (3 PE) in hypoxia, scale bar: 10 μm. (G) Cell viability 24 h after the photo-uncaging of Ir_2_BPIFc in hypoxia with/without pre-incubation of various inhibitors; PC: positive control, without inhibitors. (H) Western blotting of time-dependent monitoring of key protein expression in A375 cells related to ferroptosis and necroptosis after the photo-uncaging of Ir_2_BPIFc in hypoxia upon irradiation at 405 nm (1 PE); uncropped images in Fig. S36.† (I) Fold change of extracellular ATP in A375 cells detected at 12 h after treatment with Ir_2_BPIFc or hypericin (1 μM) upon irradiation with 405 nm (1 PE). (J) Confocal images of the immunofluorescence of CRT, HMGB1 and HSP70 in A375 cells upon irradiation with 405 nm (1 PE) in hypoxia; cells were treated with Ir_2_BPIFc or hypericin (1 μM) for 6 h, (I) control; (II) Ir_2_BPIFc (III) hypericin + light; (IV) Ir_2_BPIFc + light, scale bar: 10 μm. Concentration of Ir_2_BPIFc: 2 μM; *n* = 3, *p < 0.05, **p < 0.01 and ***p < 0.001.

Reviewing the unique photo-uncaging properties of Ir_2_BPIFc that fully utilizes the oxygen, the ambient water molecules, and the excess H_2_O_2_ in TME to generate multiple ROS, prompted us to investigate its PACT and PDT effects in hypoxic tumor models in depth. The photo-uncaging process of Ir_2_BPIFc in hypoxia (2% O_2_) was first reproduced in a monolayer cell model. Extensive attention has been devoted to developing near-infrared or nonlinear absorption photosensitizers, as another drawback of phototherapy comes from the scattering and absorption of incident light by tissues, body fluid, and endogenous pigments.^72^ Among them, metal complexes stand out again for their broadly tunable nonlinear photophysical properties.^73^ Given that our earlier investigation has revealed the outstanding three-photon excitation efficacy of the cyclometalated Ir(iii) complex with a *tert*-butyl phenanthroline imidazole ligand,^74^ a femtosecond laser was employed to estimate the photo-uncaging *in vitro*. The cubic relation of emission intensity *versus* incident power revealed its three-photon excitation nature, and the peak absorption cross-section was recorded at 970 nm (1.495 × 10^−78^ cm^6^ s^2^ per photon^2^) (Fig. S26†). Besides the successful photolysis upon 405 nm irradiation, Ir_2_BPIFc could also recover its fluorescence upon irradiation by a 970 nm femtosecond laser (Fig. 3B). An increased amount of Fe^2+^ in cancer cells after irradiation was also displayed (Fig. S27†). A co-localization assay showed a favorable overlay with MitoTracker Deep Red with a Pearson's correlation coefficient (PCC) of 0.72, in contrast to a PCC of only 0.26 with LysoTracker Deep Red (Fig. S28†). The cytotoxicity of Ir_2_BPIFc under various conditions was then estimated using an MTT assay. After being treated for 6 h in a simulated hypoxic atmosphere (∼2% O_2_), the photocytotoxicity (405 nm, 20 mW cm^−2^, 10 min) of Ir_2_BPIFc to A375 cells was recorded with an IC_50_ value of 2.06 μM, in contrast to its irrelevant dark cytotoxicity (IC_50_ = 16.32 μM) (Table S1†). Examined by ROS probe DCFH-DA, an enhanced DCF fluorescence intensity positively correlated with irradiation time was detected in hypoxia upon either 405 nm or 970 nm irradiation to Ir_2_BPIFc, confirming the phototherapeutic performance of Ir_2_BPIFc in hypoxic conditions (Fig. S29†). A similar tendency also appeared when using DHE as the O_2_˙^−^-specific probe. The enhancement and the translocalization of the DHE fluorescence from the cytoplasm to the nucleus indicate efficient O_2_˙^−^ generation even in hypoxia. Utilizing the ˙OH-specific probe HPF, we individually estimated cells' photoinduced ˙OH generation. For the involved Fenton reaction by the released Fe^2+^, the cells loaded with Fe^2+^ chelator phen were also examined. It was found that the fluorescence signals of HPF appeared in the cells immediately after irradiation. When incubation was continued for 30 min after irradiation, the cells without phen loaded showed a 1.5-fold fluorescence enhancement (Fig. 3C). It could be concluded that the released Fe^2+^ as a Fenton reagent takes part in the ˙OH generation in the physiological environment.

Individual investigation of the ROS generation of IrPICp*in vitro* revealed the availability of its type I PDT effects after being released by the photo-uncaging of Ir_2_BPIFc (Fig. S30†). The generation of highly reactive ˙OH and O_2_˙^−^ also led to severe lipid peroxidation (LPO), which was identified by C11–BODIPY (Fig. 3F and S31†). The generation of the reactive organic radicals and Fe^2+^ ions led us to investigate the broader disruption of redox homeostasis. Solution experiments first verified the capability of the photo-uncaging of Ir_2_BPIFc to oxidize NADH (Fig. S32†) and GSH (Fig. S33†), which was respectively hampered by the introduction of radical scavenger TEMPO and Fe^2+^ scavenger phen. The disruption effects are also displayed *in vitro* (Fig. 3D and E), resulting in a more liable microenvironment for efficient phototherapy.^75^ In this part, it was verified that the three-photon photo-uncaging of Ir_2_BPIFc is available in the cancer cell model in hypoxia, resulting in diverse ROS generation that effectively inhibits cancer cell proliferation.

*In situ* generation of ROS in mitochondria usually leads to significant mitochondrial damage. Flow cytometry by JC-1 assay showed that the photo-uncaging of Ir_2_BPIFc in A375 cells induced a proportional enrichment of the JC-1 monomer from 3.8% to 46.2% (Fig. S34†), indicating the severe loss of mitochondrial membrane potential under ROS attack. Such targeted organelle damage and LPO might trigger various cell death pathways. A flow cytometry analysis of the translocated phospholipid phosphatidylserine and the loss of plasma membrane could indicate typical PDT-induced apoptosis. Unexpectedly, after the phototherapy of Ir_2_BPIFc, 44.4% of A375 cells were first stained by propidium iodide (PI), which was not a typical apoptosis pattern (Fig. S35†). An inhibitor screening based on the MTT assay was conducted after the photoactivated therapy of Ir_2_BPIFc at the IC_50_ value (Fig. 3G). The results displayed that ferroptosis inhibitor ferrostatin-1 (Fer-1) recovered the cancer cell viability to 93.6%. Moreover, iron chelator deferoxamine (DFO) and necroptosis inhibitor necrostatin-1 (Nec-1) also showed efficiently rescued the cancer cells. Consequently, we focus on examining the hallmarks of ferroptosis and necroptosis. Previous research demonstrated the ability of ˙OH or O_2_˙^−^ generated by a type I photosensitizer to induce overloaded lipid peroxidation, which depleted glutathione peroxidase 4 (GPX4) against reductive protection from ferroptosis. Capitalizing on the potent LPO induction and GSH oxidation, 5 h after the photo-uncaging of Ir_2_BPIFc in cells, the GPX4 expression drastically decreased by 70% compared to the control group (Fig. 3H). All of the results above support the successful induction of ferroptosis in cancer cells by the photoactivated therapy of Ir_2_BPIFc. The pattern of PDT-induced necroptosis has rarely been investigated.^76–78^ Introducing photothermal therapy (PTT) seems to be a more effective way to initiate necroptosis.^79,80^ Considering that, meticulous time-dependent monitoring of the key initiating proteins of necroptosis was carried out. The silencing of caspase 8 is the switch to the necroptosis pathway.^81^ During 5 h monitoring after the photo-uncaging of Ir_2_BPIFc in A375 cells, no significant caspase 8 activation was observed (Fig. S37†). In contrast, 1 h after the photo-uncaging of Ir_2_BPIFc in A375 cells, apparent upregulation of phospho-RIPK1 and phospho-RIPK3 was observed, leading to the oligomerization of mixed lineage kinase domain-like protein (MLKL) (Fig. 3H), indicating the successful induction of necroptosis.

### Photoimmunotherapy of Ir_2_BPIFc against hypoxic melanoma

**Fig. 4:**
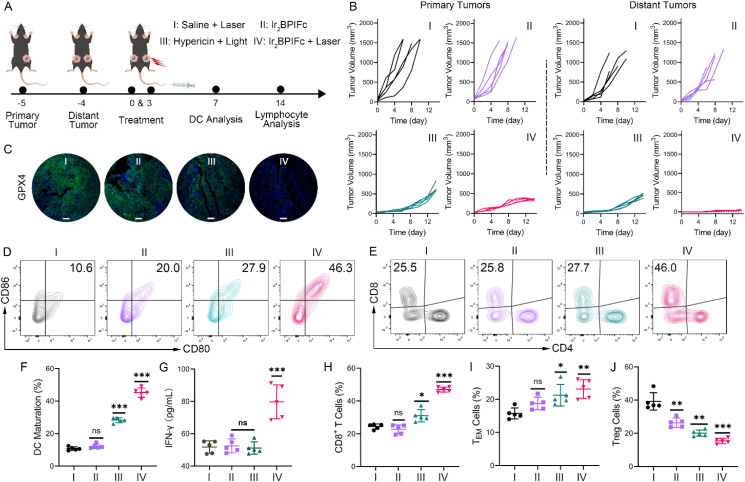
(A) Schematic illustration of the *in vivo* therapeutic protocol. (B) Statistical analysis of the volume changes of primary tumors (left) and distant tumors (right), *n* = 5, hypericin (Hyper.) *λ*_ex_ = 630 nm (1 PE) and Ir_2_BPIFc*λ*_ex_ = 970 nm (3 PE) for the optimal tissue penetration. (C) Confocal images of the immunofluorescence of GPX4 in primary tumor slices on day 14, scale bar: 100 μm. (D and E) Representative flow cytometry plots indicate the proportions of (D) mature DCs in tumor-draining lymph nodes and (E) CD8^+^ T cells in distant tumors. (F, H, I and J) Quantification of the proportion of (F) mature DCs in tumor-draining lymph nodes, (H) CD8^+^ T cells, (I) TEM cells and (J) T_reg_ cells in distant tumors. (G) The expression of IFN-γ in plasma on day 7. *n* = 5, *p < 0.05, **p < 0.01 and ***p < 0.001.

Cancer cells at certain stages of proinflammatory cell death pathways are believed to exhibit immunogenicity, which in turn triggers the host's immune response to achieve long-term cancer resistance. Immunogenic cell death (ICD) hallmark expression was estimated in human-derived melanoma cells A375 and murine-derived melanoma cells B16-F10. A red-light photosensitizer, hypericin, which has been determined to induce ICD *in vitro*,^43,82^ was introduced as the positive control. After 12 h of photoactivated therapy with Ir_2_BPIFc, the confocal images demonstrated the surface migration of calreticulin (CRT), the excretion of HMGB1 from the nucleus, and the upregulation of HSP70 (Fig. 3J). Moreover, up to a 4-fold increase of extracellular ATP was detected in the A375 cell culture supernatant in the Ir_2_BPIFc irradiation groups (Fig. 3I). Similar tendencies were also observed in B16-F10 cells (Fig. S38 and S39†). All of these indications suggest that ferroptosis and necroptosis induced by the photoactivated therapy of Ir_2_BPIFc are highly immunogenic, and their potential to activate systemic immunity is worth further investigation. Before carrying out estimation in animal models, the feasibility of 3 PE to Ir_2_BPIFc in solid tumors was of great concern. Due to the order of magnitude difference in excitation efficiency of 1 PE and 3 PE to Ir_2_BPIFc, instead of the direct estimation of the fluorescence of Ir_2_BPIFc, a high sensitivity ROS probe DCFH-DA was employed to estimate the penetration depth of 3 PE to Ir_2_BPIFc in a 3D cell spheroid model. As shown in Fig. S40,† evident fluorescence of DCF was captured after 3 PE of Ir_2_BPIFc even at a depth of 320 μm, giving promising confidence for three-photon phototherapy in the following animal estimations. An immunocompetent C57BL/6J mouse model bearing pigmented B16-F10 solid tumors was developed to evaluate the three-photon photoactivated therapeutic effect and the systemic immune response induction effect of Ir_2_BPIFc. Melanin enriched in B16-F10 solid tumors appears to have broad absorption of 300–700 nm, which severely hampers PDT treatment's effectiveness with conventional one-photon photosensitizers.^72^ PEG-formulated reagents were intravenously (i.v.) injected (1 mg kg^−1^ for hypericin and Ir_2_BPIFc), and the appropriate excitation sources (630 nm fiber optic light source for hypericin and 970 nm femtosecond laser source for Ir_2_BPIFc) were applied 12 h after injection (Fig. 4A). During 14 days of observation and recording, none of the mice in the experiment appeared to have any signs of discomfort or body weight loss, and the designed treatments did not cause visible damage to their major organs (Fig. S41 and S42†). Light irradiation alone or Ir_2_BPIFc treatment in the dark could not inhibit the tumors in group I and group II. Mice in these groups with tumor volumes that jeopardized their health had to be brought forward to the monitoring endpoint with euthanasia. On the contrary, hypericin and Ir_2_BPIFc under different phototherapeutic modules showed distinct tumor inhibition rates of 57.15% and 63.18%, respectively (Fig. 4B and S43†). The obvious down-regulation of GPX4 and upregulation of p-MLKL after phototherapy of Ir_2_BPIFc visualized by immunofluorescence verified the feasibility of three-photon photoactivation-induced ferroptosis and necroptosis in a solid tumor model (Fig. 4C, S44 and S45†).

With the satisfying tumor inhibition performance, the capacity of systemic immune response activation of Ir_2_BPIFc was investigated by inoculation of an untreated distant tumor 24 h after the treatment of the primary tumor. The distant tumors in group I and group II exponentially grew to a maximum of about 400 mm^3^ after 9 days of inoculation. Surprisingly, at the end of the observation, the tumor inhibition rates for distant tumor phototherapy in group III and group IV reached 98.71% and 90.03%, respectively (Fig. 4B). This result intuitively demonstrates the efficiency of the photoactivated therapy of Ir_2_BPIFc to evoke antitumor systemic immunity. For further evaluation of the systemic immune response, dendritic cells (DCs) in tumor-draining lymph nodes were collected after 7 days of treatment to analyze their maturation, considered one of the hallmarks of the initiation of immunogenic effects. Analysis by flow cytometry realized that the proportion of mature DC in tumor-draining lymph nodes after the photoactivated therapy of Ir_2_BPIFc increased to 44.5% on average, almost 4-fold higher than that in the negative control groups (Fig. 4D and F). 14 days after treatment, immune cell populations in the distant tumors and spleens were analyzed to assess the strength of the adaptive immune responses.

Along with the upregulation of plasmic IFN-γ (Fig. 4G), the induced ICD successfully recruited CD8^+^ T cells to the distant lesions, of which the population increased from an average of 17.39% to an average of 41.20% (Fig. 4E and H). Tumor-infiltrating regulatory T cells (T_reg_ cells, CD3^+^ CD4^+^ CD25^+^ Foxp3^+^) impede the effect of cytotoxic T cells when ICD promotes a systemic antitumor immune response. Analyses showed that the proportion of regulatory T cells in distant tumors reduced from 38.50% on average in group I to 15.20% on average in group IV (Fig. 4J and S46†). Last but not least, as an indicator of the durability of the long-term antitumor immune response, the activation level of effector memory T cells (T_em_ cells, CD3^+^ CD8^+^ CD44^+^ CD62L^−^) in distant tumors increased to 23.85% on average in group IV (Fig. 4I and S47†). To sum up, activated by a high penetration three-photon excitation, the photoactivated therapy of Ir_2_BPIFc shows potential in evoking a long-lasting antitumor immunity by ferroptosis and necroptosis-mediated ICD.

## Conclusion

In this work, we identified a novel photo-uncaging strategy by cyclometalated Ir(iii) complexes that uncovers the uncommon photoactivated homolysis of ferrocene. The excellent nonlinear optical properties of the Ir(iii) moiety first enable the three-photon excitation to reach the depths of solid tumors. The long-life triplet MLCT excited states and adequate excited potentials of the Ir(iii) moiety guarantee the accessibility of SET processes from the conjugated functional moieties, which, in this case, unearths the versatility of ferrocene. The novel homolysis of ferrocene generates the ready-to-use Fe^2+^ catalytic agent for long-lasting redox homeostasis disruption and the highly reactive carbon-centered radicals for direct electron extraction from the most abundant water molecules and other biomolecules such as NADH. This simple yet powerful molecular design meets the needs for treatments in hypoxic and pigmented solid tumor models, successfully inducing immunogenic ferroptosis and necroptosis to evoke a long-term systemic immune response to distant tumors in a bilateral B16-F10 mouse model. Not to be overlooked, in animal assessments, it was found that the therapeutic effect of Ir_2_BPIFc did not significantly differ from that of hypericin. This implies that the optimal three-photon therapeutic system may not yet have been developed under the existing laboratory conditions and also suggests that the performance of molecular photoactivated reagents still needs to be further improved. This work's concise yet practical molecular design is believed to provide a versatile platform for multi-photon photoactivated therapy against deep-seated and/or pigmented tumors.

## Statistical analysis

The significance of several experimental results was analyzed by using the analysis of *T*-test. Probabilities *p* < 0.05 (*), *p* < 0.01 (**), and *p* < 0.001 (***) are marked in the figures and 0.05 was chosen as the significance level.

## Author contributions

L. Xie and Z. Chen contributed equally to this work; Y. Chen, X. Zhang and H. Chao oversaw all experiments; L. Xie and Z. Chen performed all the experiments and wrote the manuscript; T. Wang assisted with performing and analyses of the TA experiments; J. Liang, Q. Lie and C. Jin assisted with the animal experiments; X. Zhang performed the simulation experiments. All authors discussed the results and commented on and proofread the manuscript.

## Conflicts of interest

The authors declare no competing interests.

## Supplementary Material

SC-OLF-D5SC04006J-s001

## Data Availability

ESI† is available and includes instruments, materials and methods, detailed synthesis procedures and characterization, additional biological experimental results, and DFT simulation details.
