# Supplementary material for: Photo-uncaging of a ferrocene-bridged dinuclear iridium(iii) complex for three-photon photoimmunotherapy against hypoxic melanoma
Source: Chem Sci. 2025 Jul 10;16(33):15045–55. doi: 10.1039/d5sc04006j (PMC12278504; doi:10.1039/d5sc04006j)
Supplement: SC-016-D5SC04006J-s001 [file SC-016-D5SC04006J-s001.pdf]

Supplementary Information (SI) for Chemical Science.
This journal is © The Royal Society of Chemistry 2025

SUPPORTING INFORMATION

Photo-Uncaging of a Ferrocene-Bridged Dinuclear Iridium(III) Complex for Three-Photon Photoimmunotherapy against Hypoxic Melanoma

Lina Xie,[a] ‡ Zhuoli Chen,[a] ‡ Tianying Wang,[a] Jinzhe Liang,[a] Qiaoshan Lie,[a] Chengzhi Jin,[a] Xiting Zhang,*[b] Yu Chen,*[a] Hui Chao*[a][c]

[a] MOE Key Laboratory of Bioinorganic and Synthetic Chemistry, State Key Laboratory of Anti-Infective Drug Discovery and Development, Guangdong Basic Research Center of Excellence for Functional Molecular Engineering, School of Chemistry, Sun Yat-Sen University, Guangzhou, 510006, P. R. China. E-mail: chenyu63@mail.sysu.edu.cn; ceschh@mail.sysu.edu.cn

[b] Huangpu Hydrogen Innovation Center, Guangzhou Key Laboratory for Clean Energy and Materials, School of Chemistry and Chemical Engineering, Guangzhou University, Guangzhou 510006, P. R. China. E-mail: zhxt@gzhu.edu.cn

[c] MOE Key Laboratory of Theoretical Organic Chemistry and Functional Molecule, School of Chemistry and Chemical Engineering, Hunan University of Science and Technology, Xiangtan, 400201, P. R. China

[‡] These authors contributed equally to this work

CONTENTS

Experimental Procedures 6

Materials and methods 6

Synthesis and characterization 7

Development of the experimental conditions in solution. 8

Photolysis monitoring in solution. 8

Stability in various conditions. 9

EPR detection. 9

Fe^{2+} detection in solution. 9

Hydroxyl radical detection in solution. 10

Superoxide anion detection in solution. 10

Carbon-centered radical detection in solution. 10

Estimation of excited-state energy. 10

Computational details. 11

Femtosecond transient absorption (fs-TA) Spectra. 11

Three-photon absorption cross-section measurements. 12

Cell lines and culture conditions. 12

Cell viability assay. 12

Subcellular localization. 12

ROS detection *in vitro* 13

Mitochondrial membrane potential (MMP) loss detection. 13

Lipid peroxidation *in vitro* 13

Fe^{2+} detection *in vitro*. 14

Hydroxyl radical detection *in vitro* 14

Cell death mechanism. 15

Western blotting. 15

Immunofluorescence of CRT, HMGB1 and HSP70 *in vitro*. 15

Extracellular ATP detection assay *in vitro*. 16

ROS detection in 3D cell spheroids. 16

In vivo photoimmunotherapy. 17

Ethics Statement. 18

Scheme S1. 19

Figure S1. 20

Figure S2. 20

Figure S3. 21

Figure S4. 21

Figure S5. 22

Figure S6. 23

Figure S7. 24

Figure S8. 24

Figure S9.. 25

Figure S10.. 25

Figure S11.. 26

Figure S12. 26

Figure S13. 27

Figure S14. 27

Figure S15. 28

Figure S16. 29

Figure S17. 30

Figure S18. 31

Figure S19. 31

Figure S20. 32

Figure S21. 33

Figure S22. 34

Figure S23. 34

Figure S24. 34

Figure S25. 35

Figure S26. 35

Figure S27. 36

Figure S28. 37

Figure S29. 37

Figure S30. 38

Figure S31. 38

Figure S32. 39

Figure S33. 40

Figure S34.. 40

Figure S35. 41

Figure S36. 41

Figure S37. 42

Figure S38. 43

Figure S39. 44

Figure S40. 45

Figure S41. 46

Figure S42. 47

Figure S43. 48

Figure S44. 49

Figure S45. 49

Table S1... 50

Table S2... 50

Table S3... 51

Experimental Procedures

Materials and methods

Unless noted otherwise, all reagents and solvents were commercially available and used without further purification. Double-distilled (DD) water was used throughout all the experiments. 2-phenylpyridine (ppy), 1,10-phenanthroline-5,6-dione, 4-tert-butylaniline, 3,3',5,5'-tetramethylbenzidine (TMB) and 1,10-phenanthroline were purchased from Energy Chemical. $IrCl_3 \cdot xH_2O$, ammonium acetate, acetic acid, 1,1'-ferrocenedicarboxaldehyde and D_2O were obtained from Aladdin. 5,5-dimethyl-1-pyrroline-N-oxide (DMPO) was purchased from Dojindo. PBS, Tris, Tween-20 and 3-(4,5-dimethylthiazol-2-yl)-2,5-diphenyltetrazolium bromide (MTT) were purchased from Sangong Biotech. 2,2,6,6-Tetramethylpiperidin-1-oxyl (TEMPO) was purchased from Energy Chemical. C11-BODIPY and hydroxyphenyl fluorescein (HPF) was broughted from Invitrogen. FeRhoNox-1 was obtained from MKBio. 2',7'-dichlorofluorescin diacetate (DCFH-DA), mitochondria membrane potential (JC-1) Kit, 3-Methyladenine, cycloheximide, and necrostatin-1 were purchased from Beyotime Biotechnology. Ferrstain-1 and z-VAD-fmk were purchased from Promega. Dulbecco's modified eagle medium (DMEM), fetal bovine serum (FBS), penicillin and streptomycin were purchased from Gibco. BCA protein assay reagent kit was purchased from Beyotime Biotechnology. Rabbit monoclonal anti-GPX4 antibody and anti-GAPDH antibody were purchased from Abcam. ECL prime western blot detection reagent was purchased from Thermo. Stock solutions of **Ir_2BPIFc** complexes (10 mM) were prepared in DMSO and stored in dark conditions.

Electrospray mass spectra (ESI-MS) were recorded on an LCQ system (Finnigan MAT, USA). ^{1}H NMR was recorded on Varian INOVA 600NB. UV-Vis spectra were recorded on a Perkin-Elmer Lambda 850 spectrophotometer. Instruments for western blot experiments were recorded from Bio-Rad. Confocal cell imaging was conducted on LSM 880 (Carl Zeiss, Germany) Laser Scanning Confocal Microscope. Flow cytometry experiments were conducted on BD FACS Canto II. High-resolution electrospray ionization mass spectra (HRMS) were recorded by

ESI-Q-TOF maxis 4G (Bruker Daltonics). The ESR measurements were carried out with a Bruker Model A300 spectrometer at 298 K. Elemental analyses were carried out with the Elementar Vario EL Cube CHNS/O elemental analyzer.

Synthesis and characterization

Synthesis of BPIFc. 1,10-phenanthroline-5,6-dionec (420.0 mg, 2.0 mmol, 2.0 eq), 4-tert-butylaniline (298.0 mg, 2.0 mmol, 2.0 eq), 1,1'-ferrocenedicarboxaldehyde (242.0 mg, 1.0 mmol, 1.0 eq) and ammonium acetate (154.0 mg, 5.0 mmol, 5.0 eq) were suspened in the 30 mL acetic acid and refluxed at 120°C for 24 h. After that, 30 mL water was added to the mixture and the resultant precipitate was collected by filtration and washed with water. The dryed crude product was dissolved in the dichloromethane and purified by column chromatography on silica gel using CH_2Cl_2 and CH_3OH (10:1-5:1) as the eluent, to get an red solid. Yield: 35%.. ^{1}H NMR (600 MHz, Chloroform-*d*) δ 8.74 (dd, *J* = 4.2, 1.4 Hz, 1H), 8.25 (d, *J* = 7.8 Hz, 1H), 8.03 (d, *J* = 7.4 Hz, 2H), 7.82 (d, *J* = 8.3 Hz, 2H), 7.05 (dd, *J* = 8.3, 4.2 Hz, 1H), 6.97 – 6.89 (m, 2H), 4.44 (s, 2H), 4.25 (t, *J* = 1.9 Hz, 2H), 1.58 (s, 9H). ^{13}C NMR (151 MHz, Chloroform-*d*) δ 154.43, 149.58, 147.90, 146.76, 143.43, 143.05, 135.81, 135.44, 128.95, 128.62, 127.40, 126.83, 126.38, 121.98, 121.67, 121.56, 119.04, 70.27, 69.28, 35.26, 31.58. ESI-MS: *m/z* 887.64 [M-H]$^+$. HRMS (ESI): m/z calcd for $C_{56}H_{47}FeN_8^+$: 887.3267, found: 887.3276. Anal calcd for $C_{56}H_{46}FeN_8$: C, 75.84; H, 5.23; N, 12.63; found C 75.73; H, 5.29; N, 12.51.

Synthesis of Ir_2BPIFc complex. In a general procedure, 1.0 mmol $[Ir(ppy)_2Cl_2]_2$ and 1.0 mmol **BPIFc** complex were placed in a 50 mL three-necked flask with 20 mL of methanol and chloroform (1:3, v/v) and refluxed at 65°C for 8 h under argon. After that, the solvent was removed under reduced pressure, the crude product was purified by column chromatography on aluminum oxide with CH_2Cl_2/CH_3OH (20:1, v/v) as the eluent to get an orange solid. Yield: 54%. ^{1}H NMR (600 MHz, Acetonitrile-*d3*) δ 9.05 (d, *J* = 8.1 Hz, 1H), 8.59 (d, *J* = 8.1 Hz, 1H), 8.27 (d, *J* = 3.9 Hz, 1H), 8.18 (s, 2H), 8.11 (t, *J* = 8.2 Hz, 2H), 8.07 (d, *J* = 8.0 Hz, 2H), 7.90 (d, *J* = 8.9 Hz, 3H), 7.86 – 7.81 (m, 4H), 7.74 (dt, *J* = 15.2, 8.7 Hz, 4H), 7.66 (d, *J* = 7.3 Hz, 2H), 7.53 (d, *J* = 10.3 Hz, 3H), 7.49 (s, 2H), 7.46 (d, *J* = 8.1 Hz, 3H), 7.40 (d, *J* = 8.3 Hz, 1H), 7.31 (d, *J* = 5.6 Hz, 1H), 7.27 (d, *J* = 7.2 Hz, 1H), 7.24 – 7.20 (m, 2H), 7.16 (dt, *J* = 15.7, 7.3 Hz, 2H), 7.10 (t, *J* = 7.5

Hz, 2H), 7.06 (t, *J* = 7.2 Hz, 1H), 7.00 (dt, *J* = 13.7, 6.8 Hz, 4H), 6.91 (dt, *J* = 12.7, 6.6 Hz, 3H), 6.86 (t, *J* = 6.4 Hz, 1H), 6.43 – 6.36 (m, 4H), 4.67 (s, 1H), 4.53 (s, 1H), 4.40 (d, *J* = 10.5 Hz, 2H), 4.29 (d, *J* = 10.0 Hz, 2H), 4.17 (s, 1H), 4.07 (s, 1H), 1.41 (s, 9H), 1.34 (s, 9H). ^{13}C NMR (151 MHz, DMSO-d_6) δ 166.83, 166.79, 166.75, 154.47, 154.16, 154.04, 150.36, 150.30, 149.96, 149.32, 148.99, 144.00, 143.97, 143.85, 143.71, 138.64, 136.34, 136.32, 133.64, 132.08, 131.03, 130.20, 129.15, 128.18, 128.07, 127.65, 127.59, 127.51, 127.31, 126.38, 125.38, 125.30, 125.03, 123.81, 122.30, 121.70, 121.66, 119.96, 73.87, 73.80, 72.63, 72.55, 72.41, 72.26, 69.59, 69.08, 68.91, 30.98, 30.96. ESI-MS: *m/z* 944.42 $[M]^{2+}$. HRMS (ESI): m/z calcd for $C_{100}H_{78}FeIr_2N_{12}^{2+}$: 944.2535, found: 944.2530. Anal calcd for $C_{100}H_{78}Cl_2FeIr_2N_{12}$: C, 61.31; H, 4.01; N, 8.58; found C 61.28; H, 4.28; N, 8.21. Purity (HPLC): t_R: 7.66 min, 99.09% at 254 nm.

Development of the experimental conditions in solution.

To develop the hypoxic conditions (2% O_2), the working solution was stirred and incubated in a binary-component gas incubator (LongYue) with a digital oxygen controller for 4 h.

Photolysis monitoring in solution.

The solution of **Ir$_2$BPIFc** (10 μM) in PBS buffer was irradiated with 405 nm LED (20 mW/cm^2) or kept in the dark. The UV-Vis absorption, emission, ESI-MS, and HRMS spectrum of sample solutions was recorded in certain time sequences. For the HPLC experiment, the solution of **Ir$_2$BPIFc** (10 μM) was irradiated with LED (20 mW/cm^2) and aliquots were collected every two minutes for analyses. The UV/vis detector of the HPLC apparatus was set at 365 nm, and the eluent were CH_3CN and H_2O. Diazepam was used as an internal reference.

Stability in various conditions.

Ir$_2$BPIFc (10 μM) was incubated with GSH (10 mM), H_2O_2 (200 μM) in PBS in the dark, aliquots were collected at 0, 4, 8 ,12, 24 h for analyses. For the examination of the stability of **Ir$_2$BPIFc** in cells, A375 cells were incubated with **Ir$_2$BPIFc** (5 μM) for 0, 4, 8 ,12, 24 h in the dark, cells were then washed, collected and lysed. Cell lysates were precipitated by acetonitrile and the supernatant was concentrated, redissolved in acetonitrile and filtrated for HPLC analyses.

The UV/vis detector of the HPLC apparatus was set at 365 nm, and the eluent were CH_3CN and H_2O. Diazepam was used as an internal reference.

EPR detection.

The radical generation was detected through electron paramagnetic resonance spectroscopy (EPR). Samples were irradiated with 405 nm LED (20 mW/cm^2, 5 min) or kept in dark. For the examination of singlet oxygen ($^1O_2$), 2,2,6,6-Tetramethylpiperidine (TEMP) was employed as the spin trap, the working solution was prepared in H_2O. For the examination of hydroxyl radical (·OH), 5,5-Dimethyl-1-pyrroline *N*-oxide (DMPO) was employed as the spin trap, the working solution was prepared in H_2O. For the examination of superoxide anion ($O_2^{\cdot-}$), DMPO was employed as the spin trap, the working solution was prepared in DMSO. For the examination of oorganic radical, POBN was employed as the spin trap, the working solution was prepared in dioxane. All measurements were carried out at 20 mW microwave power, 100 G-scan range, and 1 G field modulation.

Fe^{2+} detection in solution.

The release of Fe^{2+} was detected by phenanthroline (phen) coordination. **Ir_2BPIFc** (10 μM) with/without phen (1 mM) in acetonitrile/H_2O (1/1,v/v) was irradiated at 405 nm LED (20 mW/cm^2, 10 min) or kept in the dark. Afterward, the absorption spectra of the mixture were recorded.

Hydroxyl radical detection in solution.

3,3′,5,5′-tetramethyl-benzidine (TMB) was employed as the ·OH probe. **Ir_2BPIFc** working solution was prepared at 10 μM in PBS in hypoxia (2% O_2) and then irradiated at 405 nm LED (20 mW/cm^2). The absorbance of TMB at 652 nm was recorded by UV-vis absorption spectrometer. TEMPO radical was employed as the organic radical scavenger. For the examination of ·OH generated by the Fenton reaction of the released Fe^{2+}, phen (1 mM) was employed as the Fe^{2+} scavenger, and H_2O_2 (100 μM) as the Fenton reaction fuel.

Superoxide anion detection in solution.

dihydroethidium (DHE) was employed as the $O_2^{\cdot-}$ probe. **Ir_2BPIFc** working solution was prepared at 10 μM in DMSO in hypoxia (2% O_2) and then irradiated at 405 nm LED (20 mW/cm^2). The fluorescence of DHE at 610 nm was recorded by an emission spectrometer.

Carbon-centered radical detection in solution.

2,2'-azino-bis(3-ethylbenzothiazoline-6-sulfonic acid) (ABTS) was employed as the probe. **Ir_2BPIFc** working solution was prepared at 10 μM in dioxane in hypoxia (2% O_2) and then irradiated at 405 nm LED (20 mW/cm^2). The absorbance of ABTS at 750 nm was recorded by UV-vis absorption spectrometer.

Estimation of excited-state energy.

The excited-state energy was determined according to a literature method based on Rehm–Weller theory,

$$E^*(X^*/X^-) = E(X/X^-) + E_{00}(X^*/X)$$

$$E^*(X^+/X^*) = E(X^+/X^*) - E_{00}(X^*/X)$$

$$E_{00}(X^*/X) \sim hc/\lambda$$

where h is Planck constant (6.62×10^{-34} J·s), c is lightspeed (3×10^8 m·s^{-1}), λ is the wavelength that corresponds to the crossing point between the absorption and the emission profile of the species.

Computational Details.

B3LYP method DFT calculations were performed with a 6-31G(d) basis set for C, H, N and Cl atoms and SDD basis set for Ir and Fe atoms. Frequency calculations using the identical method and basis set were done to determine that the geometries were at local minima (with all-real frequencies). TD-B3LYP/SDD(Ir, Fe)-6-31G*(H_2O) calculations were used to compute the UV-vis spectra. The effect of solvent polarity on the stability of the relevant species was examined with structural optimization calculations done using the integral equation formalism

polarizable continuum model (IEFPCM) in H_2O (e=78.5) in which the nonelectrostatic terms and the radii used the universal solvation model (SMD) developed by Truhlar and co-workers. The Gibbs free energy ΔG was used in the discussion of the result. All of computations were done with the gaussian 16 program.

Femtosecond Transient Absorption (fs-TA) Spectra.

TA spectra were recorded on a femtosecond pump–probe detection system. The laser is generated from an ultrafast laser amplifier (Coherent, central wavelength: 800 nm, repetition rate: 1 kHz, pulse width: 100 fs, pulse energy: 6 mJ). The output laser is divided into two beams of light with a beam splitter. The reflected light is applied to a pump optical parametric amplifier (OPA, Spectra-physics, TOPAS) to generate a pump beam of different wavelengths (250–2500 nm). The pump beam is converted to 500 Hz by a chopper, which is used to excite the sample, and its intensity is tuned via a continuously variable neutral density filter wheel. The transmitted light was used to generate the continuous white light (350–800 nm) via a CaF_2 crystal and acted as the probe beam. Then, the probe beam is collected by a fiber-coupled spectrometer after the sample. Using an optical delay line, the delay between the pump and probe beams is controlled. The excitation wavelength for the fs-TA experiment was selected at 390 nm.

Three-photon absorption cross-section measurements.

Three-photon cross-section was detected according to the previous report.[1] Rhodamine 6G was used as the reference sample. The **Ir_2BPIFc** and rhodamine 6G were prepared in CH_2Cl_2 at a concentration of 10 mM and 100 µM, respectively. Three cross-section was detected flowing the equation.

$$\frac{f_3}{f_2} = \frac{\eta_3 \sigma_3 I_0}{\eta_2 \sigma_2}$$

where σ is the three-photon cross-section, f is the fluorescence intensity, η is quantum efficiency, I is laser power (8 mW). The subcript 2 is refers to the process of two-photon excitation process, and 3 is refers to the process of three-photon excitation. The exictation of **Ir_2BPIFc** was measured at 900 – 1040 nm. In this experiment, the spot size of the focus is d =

0.45 mm, the distance between the lens (K9 glass planoconvex lens) and the sample is 9.5 cm.

Cell lines and culture conditions.

The melanoma cell line A375 and B16-F10 were purchased from the Experimental Animal Center at Sun Yat-Sen University (Guangzhou, China). The cell was maintained at Dulbecco's modified Eagle's medium (DMEM, Gibco BRL) with 10% (v/v) fetal bovine serum (FBS, Gibco BRL), 100 μg/mL streptomycin, and 100 U/mL penicillin (Gibco BRL) under 5% CO_2 and 95% air atmosphere. All the hypoxia experiments were performed in a LongYue hypoxia station.

Cell viability assay.

The cell viability was determined by MTT assay. Briefly, the cells were seeded in 96-well microplate and incubated for 24 h to adhere. Afterward, the cell was transferred into a hypoxia (2% O_2) incubator for another 24 h incubation. The cells were cultured with different concentrations of **Ir_2BPIFc** for 6 h. After that, the culture medium was removed and then the cells were washed three times with fresh medium. The light group was irradiated at 405 nm LED (20 mW/cm^2) for 10 min while the dark group was not exposed to LED irradiation. After 18 h incubation, MTT (20 μL, 5 mg/mL) solution was added to each well and incubated for another 4 h. The culture medium was removed and 150 μL of DMSO solution was added to dissolve the formazan crystals. The optical density of each well was measured on a microplate spectrophotometer at a wavelength of 595 nm. Data were reported as the means ± standard deviation (n = 3).

Subcellular localization.

The cells were seeded in the 2 cm plates and incubated for 24 h for adherence. The cells were transfered hypoxic incubator for another 24h incubation. Afterward, the cells were incubated with 2 μM **Ir_2BPIFc** complex for 3 h. After that, the culture medium was removed and maintained in fresh medium containing MitoTracker Deep Red (500 nM) and Lysotracker Deep Red (500 nM) for 30 min. Finally, the cell was washed three times with phosphate buffer saline and irradiated by LSM 880 NLO (Carl Zeiss) laser scanning confocal microscope. **Ir_2BPIFc**

($\lambda_{ex/em}$ = 970/550 ± 10 nm), MTDR ($\lambda_{ex/em}$ = 633/670 ± 10 nm), LTDR ($\lambda_{ex/em}$ = 633/656 ± 10 nm)

ROS detection *in vitro*

a. Laser scanning confocal microscopy. A375 cells were seeded in 2 cm quartz dishes and incubated in normoxic conditions for 24 to adhere. The cells were then transferred into hypoxic conditions for another 24 h incubation. After that, A375 cells were added 2 µM **Ir$_2$BPIFc** and incubated for another 6 h. The culture medium in cells was removed and the cells were maintained in fresh medium containing 1.0 µM DCFH-DA or 1.0 µM DHE for 30 min. Finally, the cells were transferred into hypoxic microincubator in micoscope and maintained for another 2 h. The cells were irradiated with 405 nm (1PE) or 970 nm (3PE) in hypoxic conditions (2% O_2) by Zeiss LSM 880 NLO confocal microscope. DCF: λ_{ex} = 488 nm, λ_{em} = 525 ± 10 nm; DHE: λ_{ex} = 561 nm, λ_{em} = 625 ± 10 nm.

b. Flow cytometry. A375 cells were seeded in 6-well plates and allowed to adhere upon incubation for 24 h. The cells were then transferred into a hypoxic incubator for 24 h. A375 cells were treated with **Ir$_2$BPIFc** (2 µM) for 6 h in the dark. Then culture medium was removed and the cell was maintained in fresh medium with 1.0 µM DCFH-DA. Finally, the cells were irradiated at 405 nm (20 mW/cm^2, 10 min) in hypoxic conditions (2% O_2) or maintained in the dark. The ROS generation in all group was detected using the flow cytometry in FITC channel.

Mitochondrial membrane potential (MMP) loss detection.

MMP loss was measured with JC-1 assay kit by flow cytometry in FITC and PE channels, and the protocol are the same as mentioned above.

Lipid peroxidation *in vitro*

a. Laser scanning confocal microscopy. A375 cells were seeded in 2 cm quartz dishes and incubated in normoxic conditions for 24 h to adhere. The cells were then transferred into hypoxic incubator for another 24 h. After that, A375 cells were added 2 µM **Ir$_2$BPIFc** and incubated for another 6 h. The culture medium was then replaced with fresh medium after being washed three times with phosphate-buffered saline. The cells were transferred into the

hypoxic microincubator in micoscope and maintained for another 2 h. The cells were irradiated with 970 nm (3PE) for 10 min in hypoxic conditions (2% O_2) by Zeiss LSM 880 NLO confocal microscope. The cells were then incubated in fresh medium containing 1.0 µM C11-BODIPY for 30 min. After washing, the lipid peroxidation induced by irradiation was recorded by Zeiss LSM 880 NLO confocal microscope. C11-BODIPY: λ_{ex} = 488 nm, λ_{em} = 525 ± 10 nm.

b. Flow cytometry. A375 cells were seeded in 6-well plates and allowed to adhere upon incubation for 24 h. The cells were then transferred into a hypoxic incubator for 24 h. A375 cells were treated with **Ir_2BPIFc** (2 µM) for 6 h in the dark conditions. Then culture medium was refrashed and kept in the hypoxic incubator for another 2 h. The cells were irradiated at 405 nm (20 mW/cm^2, 10 min) in hypoxic conditions (2% O_2) or kept in the dark. The cell was then stained with 1.0 µM C11-BODIPY for 30 min in dark conditions. Finally, the lipid peroxidation generation in all groups was detected using FITC channel.

Fe^{2+} detection *in vitro*.

Fe^{2+} was probed by RhoNox-1 staining and the protocol are the same as the peroxidation generation detection assay mentioned above. RhoNox-1: λ_{ex} = 535 nm, λ_{em} = 575-610 nm.

Hydroxyl radical detection *in vitro*.

Hydroxyl radical generated in cells was measured by HPF staining in laser scanning confocal microscopy. A375 cells were seeded in 2 cm quartz dishes and incubated in normoxic conditions for 24 to adhere. The cells were then transferred into hypoxic incubator for another 24 h. After that, A375 cells were added **Ir_2BPIFc** (2 µM) with/without phen (100 µM) and incubated for another 6 h. The cells were transferred into the hypoxic microincubator in micoscope and maintained for another 2 h. The cells were irradiated with 970 nm (3PE) for 10 min in hypoxic conditions (2% O_2) by Zeiss LSM 880 NLO confocal microscope. Confocal images were captured right after the irradiation and again 30 min atfer the irradiation. HPF: λ_{ex} = 488 nm, λ_{em} = 515 ± 10 nm.

Cell death mechanism.

A375 cells were seeded in the 96 plates and incubated for 24 h to adhere. The cell was transferred into hypoxic incubator for 24 h and then pretreated with ferrostain-1 (10.0 µM), necrostatin-1 (30.0 µM), z-VAD-fmk (20.0 µM), 3-methyladenine (100.0 µM) and deferoxamine (100 µM) for 1 h. Subsequently, the cells was treated with 2 µM **Ir_2BPIFc** complex and incubated for 6 h. After this time, cell culture medium was replaced with fresh medium after the cell was washed three times with phosphate buffer saline. The light group was irradiated at 405 nm LED (20 mW/cm^2) for 10 min while the dark group was not exposed in LED irradiation. After incubation for another 18 h, the cell viability was measured by MTT assay.

Western blotting.

A375 cells were seeded in 10 cm culture plates and incubated for overnight to adhere. The cell was transferred into a hypoxic incubator for 24 h and treated with **Ir_2BPIFc** (2 µM) for 6 h. Subsequently, cells were washed with PBS for 3 times and then maintained in fresh medium. The light group was irradiated at 405 nm LED (20 mW/cm^2) for 10 min while the dark group was not exposed in LED irradiation. After being incubated for different times, the collected cells were treated with RIPA combined with protease inhibitor cocktail for 30 min on ice. The lysates were centrifuged at 12000 rpm for 15 min at 4 °C. The protein concentrations were detected by the BCA protein assay reagent kit (Novagen Inc, USA). For the blotting of phosphoproteins, the blots were first probed with the relative phosphoprotein antibodies, then stripped by stripping buffer (EpiZyme, China) and re-probed with antibodies against the total protein to serves as a control.[2]

Immunofluorescence of CRT, HMGB1 and HSP70 *in vitro*.

A375 or B16F10 cells were seeded in 2 cm quartz dishes and incubated in normoxic conditions for 24 to adhere. The cells were then transferred into hypoxic incubator for another 24 h. After that, A375 cells were added **Ir_2BPIFc** (2 µM) or hypericin (1 µM) and incubated for another 6 h. The culture medium was then replaced with fresh medium after being washed

three times with phosphate-buffered saline. The cells were irradiated at 405 nm (20 mW/cm^2, 10 min) in hypoxic conditions (2% O_2) or maintained in the dark. After another 12 h incubation, the cells were processed and stained with Calreticulin (D3E6) XP® Rabbit mAb and Anti-Rabbit IgG (H+L) (Alexa Fluor® 488 Conjugate) for CRT, HMGB1 Antibody and Anti-rabbit IgG (H+L), F(ab')2 Fragment (Alexa Fluor® 555 Conjugate) for HMGB1 and anti-Hsp70 Rabbit mAb and Anti-Rabbit IgG (H+L),F(ab')2 Fragment (Alexa Fluor® 555 Conjugate) for HSP70, according to the manufacturer's protocol, and then Hoechst 33342 for 15 min after being washed with PBS. After washing with PBS again, the cells were imaged by the confocal microscope.

Extracellular ATP detection assay *in vitro*.

The extracellular ATP was detected by ATP Bioluminescence Detection Kit (Promega). The cells were seeded in opaque-walled plates and treated the same way as in immunofluorescence *in vitro*, 100 µL supernatant was mixed with 100 µL Reagents for each assay. The Chemiluminescence Signal of ATP was measured by the TECAN Infinite M200 PRO multifunctional reader. The actual values of ATP content in each group were corrected by cell viability under each culture condition. The data were reported as mean ± standard deviation (SD) (n = 3).

ROS detection in 3D cell spheroids.

A suspension of A375 cells at 2*10^4 cells/mL was added to the 1.5% agarose-coated 96-well plates, reaching a total volume of 200 µL/well. The culture medium was refreshed every two days. A375 3D cell spheroids formed within 3 days with an average diameter of 500 µm. The spheroids were incubated with **Ir_2BPIFc** (2 µM) for 24 h in the dark in hypoxic conditions (2% O_2). Then, the cell spheroids were transferred into a hypoxic microincubator in the microscope and maintained for another 2 h. The cell spheroids were irradiated with 405 nm (1PE) or 970 nm (3PE) in hypoxic conditions (2% O_2) by Zeiss LSM 880 NLO confocal microscope using z-stack mode with a 10 µm interval. Finally culture medium was refreshed with fresh medium containing 1.0 µM DCFH-DA for 2 h. DCF was incubated after the z-stack irradiation to avoid photobleaching, and for the best resolution of DCF signals in different depths, the fluorescence

of DCF was recorded with two-photon excitation ($\lambda_{ex/em}$ = 740/525 ± 10 nm).[1]

***In vivo* photoimmunotherapy**.

4-6 weeks female C57BL/6J mice were purchased from Beijing Vital River Laboratory Animal Technology Co., Ltd.. 2*10^5 B16F10 cells in 100 µL of a mixture with PBS and Matrigel (Corning) (v/v: 7/3) were subcutaneously injected in right flanks of mice (marked as primary tumor). After 1 days, B16F10 cell suspension was subcutaneously injected in the left flanks of mice (marked as the distant tumor). Then the tumor-bearing mice were randomly separated into four groups:

Group 1 (Saline + Laser): intravenous injected with 20 µL saline in the primary tumors. 12 h later, the primary tumors in mice were irradiated with 970 nm laser (50 mW, 1 kHz, pulse width 35 fs);

Group 2 (**Ir_2BPIFc** + Dark): intravenous injected with 20 µL **Ir_2BPIFc** (1.0 mg/kg) in the primary tumor.

Group 3 (Hyper. + Light): intravenous injected with 20 µL hypericin (1.0 mg/kg) in the primary tumor, 12 h later, the primary tumors in mice were irradiated with 630 nm fiber optics light for 5 min (50 mW);

Group 4 (**Ir_2BPIFc** + Laser): intravenous injected with 20 µL **Ir_2BPIFc** (1.0 mg/kg) in the primary tumor. 12 h later, the primary tumor in mice was irradiated with 970 nm laser (50 mW, 1 kHz, pulse width 35 fs);

Three-photon exictation laser irradiation was achieved with a Coherent Legend Elite laser system. After being anaesthetized, a mouse was fixed in a warm three-axes holder. The laser was focused through a convex lens with a focal length of 5 cm. Tumor was exposed to reciprocating linear scanning for 50 s/slice with 1 mm stepping. Treatments were conducted on day 0 and day 3. After treatment, the body weight and tumor volume were recorded every two days until the experiment was finished. The tumor volume was calculated by the following Eq.

$$volume = \frac{length \times width^2}{2}$$

On day 7, dendritic cells in tumor-draining lymph nodes were analyzed by flow cytometry

from 5 mice in each group. On day 14, lymphocytes from the tumors and spleens were analyzed by flow cytometry from 5 mice in each group. Samples were prepared into single-cell suspension by collagenase IV (1000 U/mL) and hyaluronidase (1000 U/mL) at 37 °C in complete culture medium. After being filtered through the 40 µm nylon strainer, cells were fixed, blocked and stained. The immunofluorescence antibodies involved included: (1) pacific blue-CD45, percpcy5.5-CD11C, FITC-CD80, PE-CD86; (2) PE-CD3, FITC-CD4, SB600 (Q-Dot)-CD8, Percp-Cy5.5-CD44, APC-CY7-CD62L; (3) PE-CY7-CD3, Percpcy5.5-CD4, APC-CD25, PE-Foxp3.

14 days after the treatment, the mice were sacrificed. The tumor tissue in each group was frozen and sliced. The slices of the tumor were analyzed by immunofluorescence of GPX4.

Ethics Statement.

The mice were raised in well-ventilated conditions, with a relative humidity of 50–60% at 20 °C. After 10 days of adaptive feeding, the mice were applied for the experiment. This study was performed with the Institutional Animal Care and Use Committee (IACUC) of Sun Yat-Sen University (Approval No: SYSU-IACUC-2023-000831). Animals were treated as the guidelines of IACUC.

Reference

[1] a) J. H. Yu, S.-H. Kwon, Z. Petrášek, O. K. Park, S. W. Jun, K. Shin, M. Choi, Y. I. Park, K. Park, H. B. Na, N. Lee, D. W. Lee, J. H. Kim, P. Schwille and T. Hyeon, *Nat. Mater.*, **2013**, *12*, 359-366; b) N. S. Makarov, M. Drobizhev, A. Rebane, *Opt. Express* **2008**, *16*, 4029-4047.

[2] Cell Signaling Technology, "Have You Ever Wondered: What's The Lowdown on Phospho-Specific Antibodies?", can be found under https://blog.cellsignal.com/have-you-ever-wondered-whats-the-lowdown-on-phospho-specific-antibodies, **2024** (assessed: Nov. 10th, 2024)

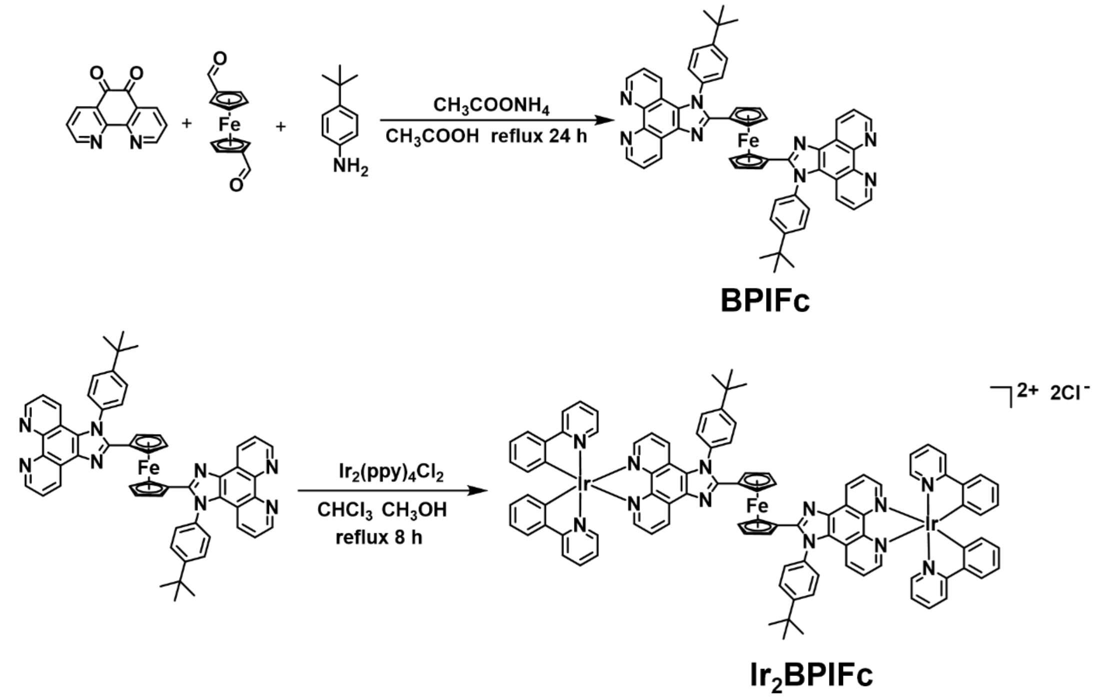

Scheme S1. Synthesis of **BPIFc** and **Ir_2BPIFc.**

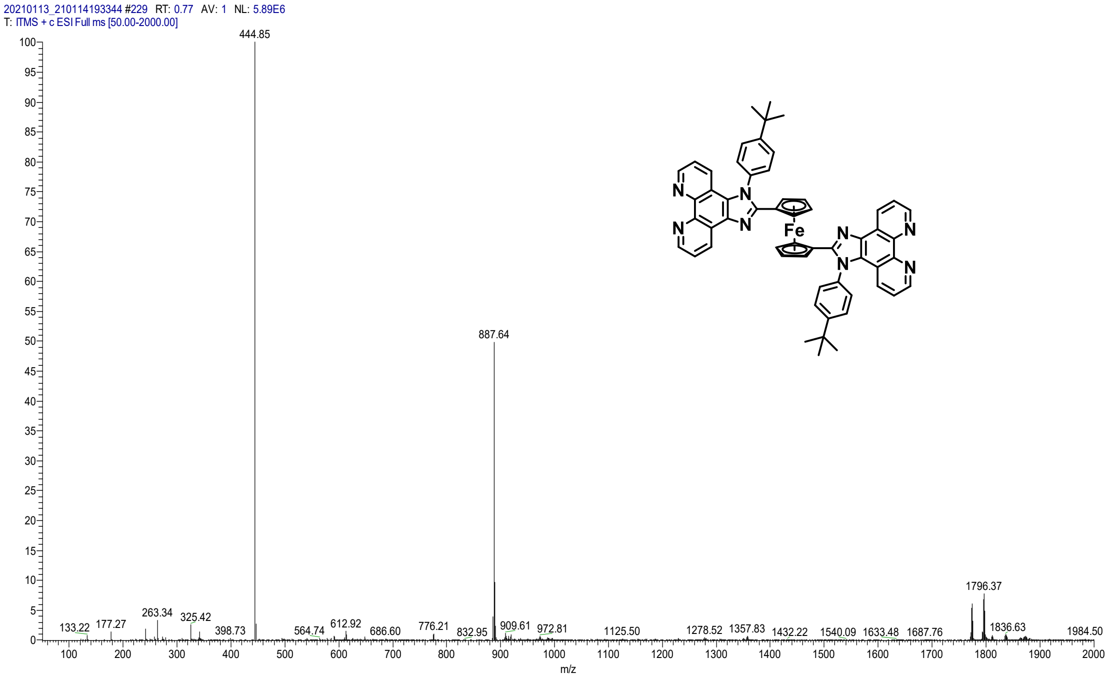

Figure S1. ESI-MS characterization of **BPIFc.**

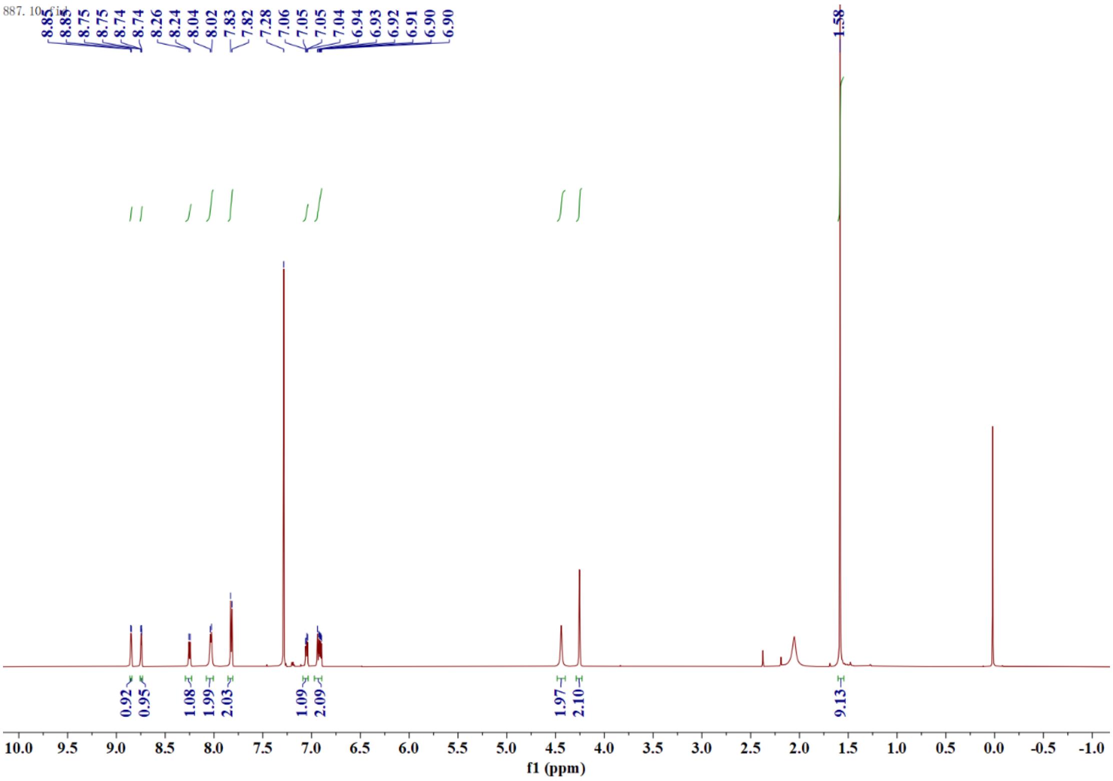

Figure S2. ^{1}H NMR spectrum of **BPIFc** in Chloroform-*d*.

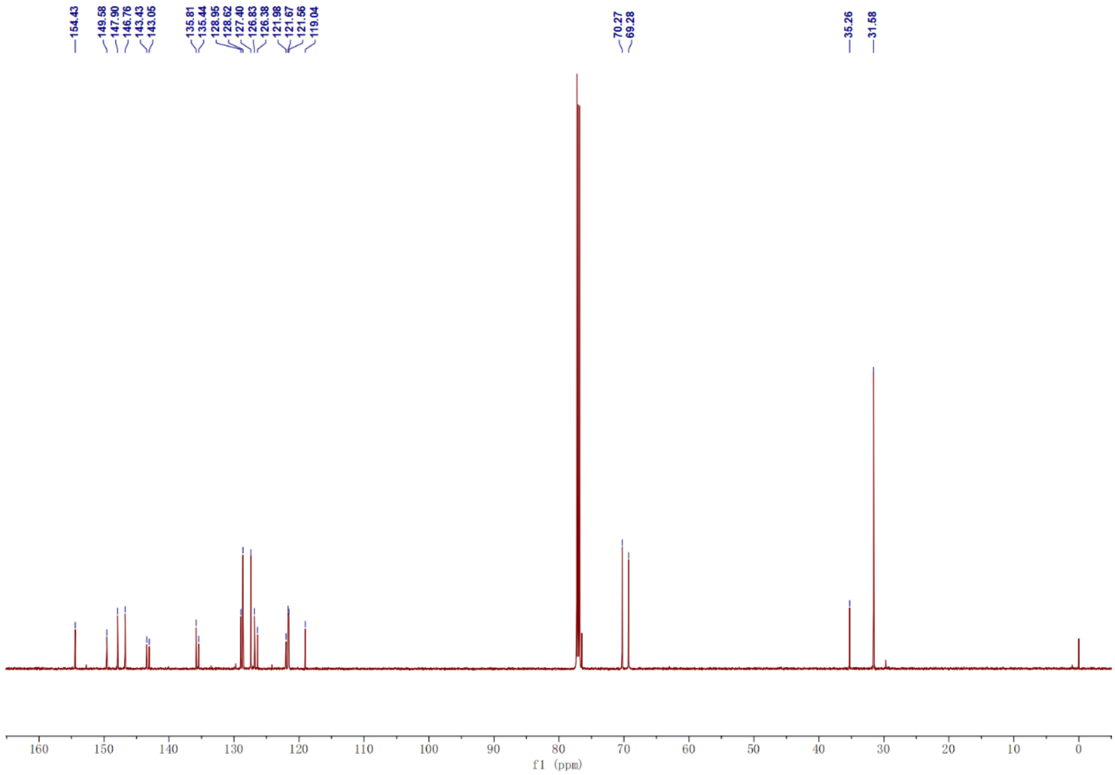

Figure S3. ^{13}C NMR spectrum of **BPIFc** in Chloroform-*d*.

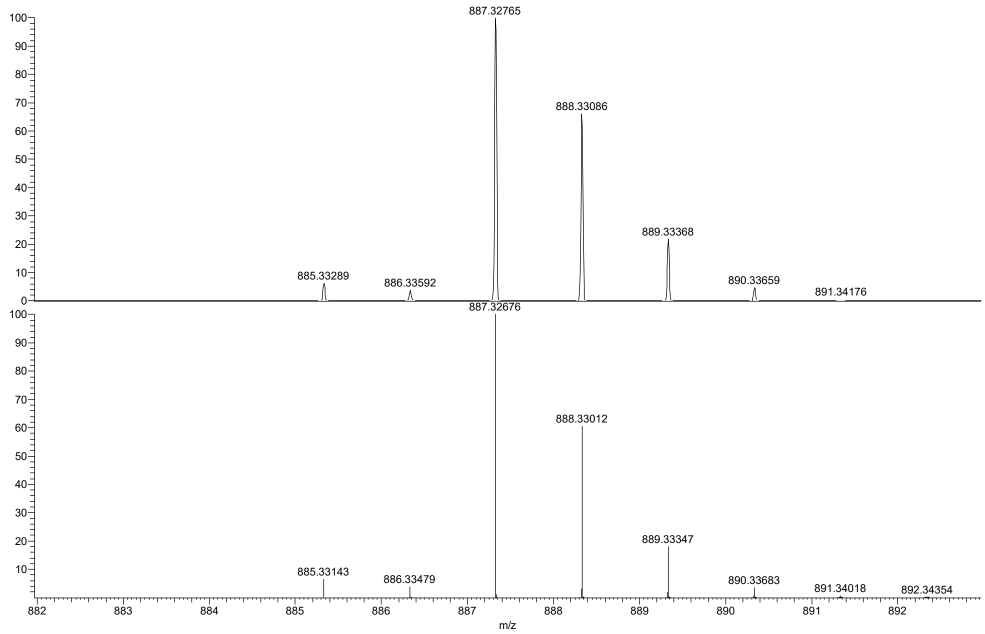

Figure S4. HR-MS characterization of **BPIFc.**

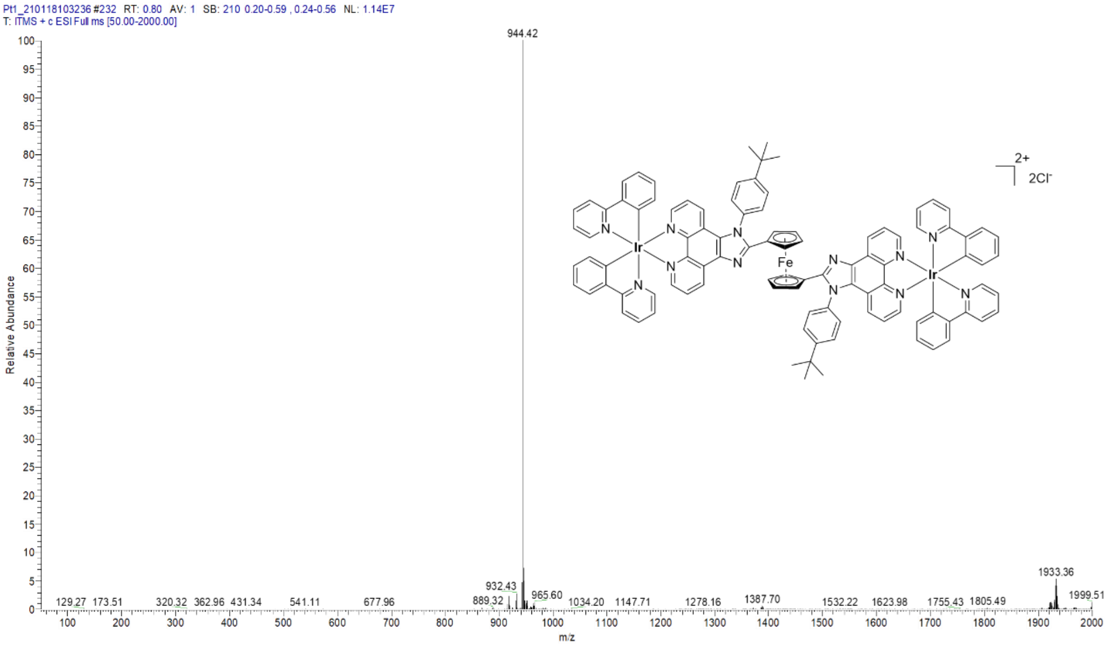

Figure S5. ESI-MS characterization of **Ir_2BPIFc.**

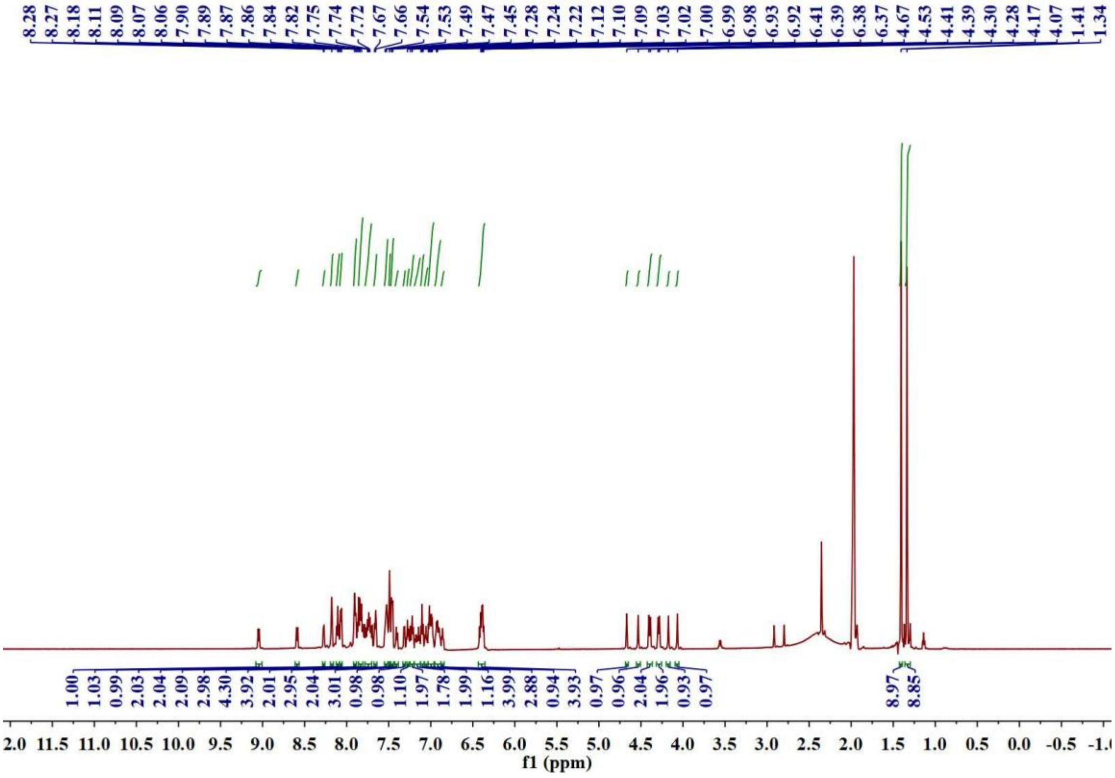

Figure S6. ^{1}H NMR spectrum of **Ir$_2$BPIFc** in acetonitrile-*d3*.

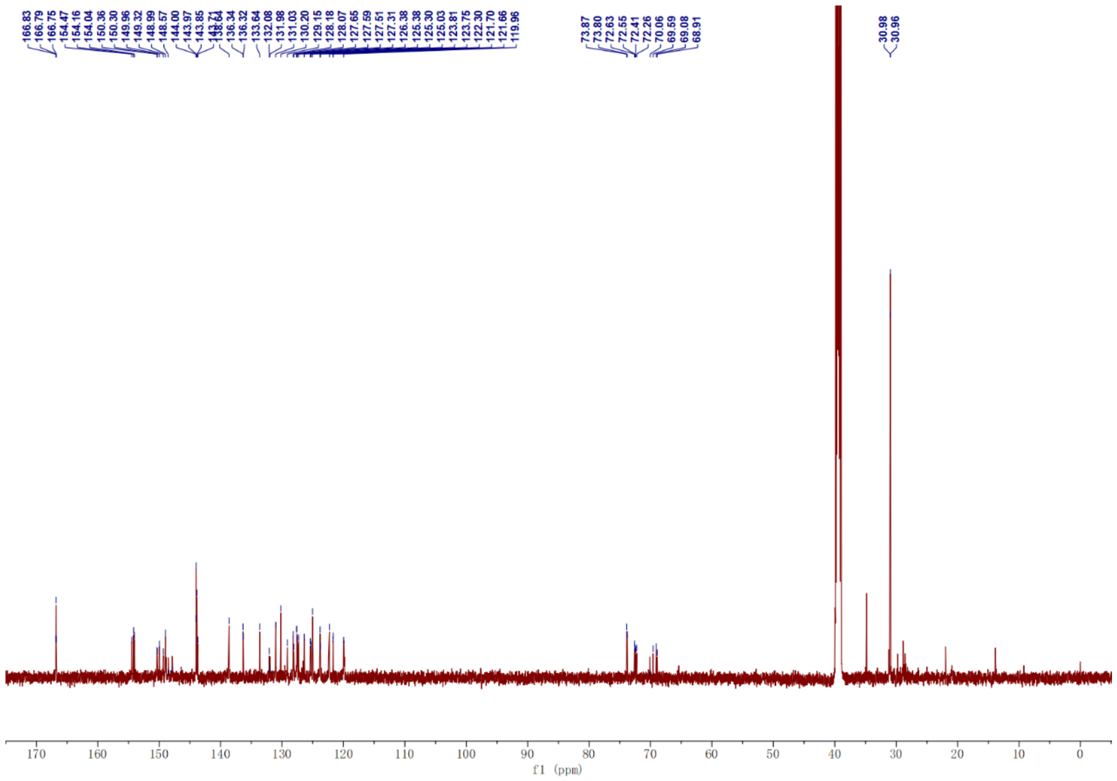

Figure S7. ^{13}C NMR spectrum of **Ir_2BPIFc** in *DMSO-d6*.

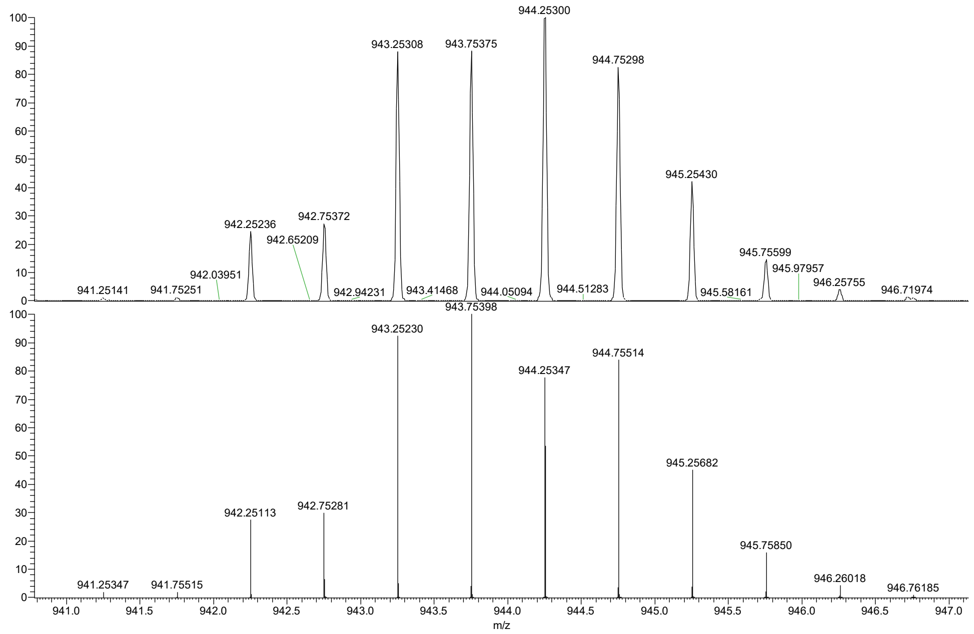

Figure S8. HR-MS characterization of **Ir_2BPIFc.**

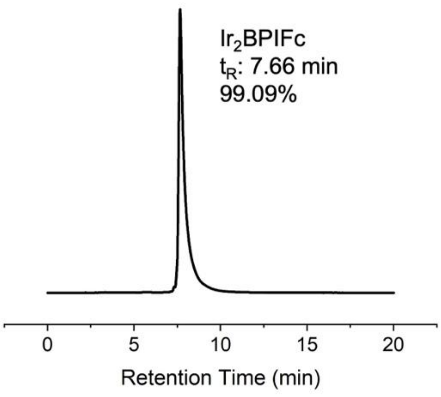

Figure S9. HPLC analysis of the purity of **Ir_2BPIFc**.

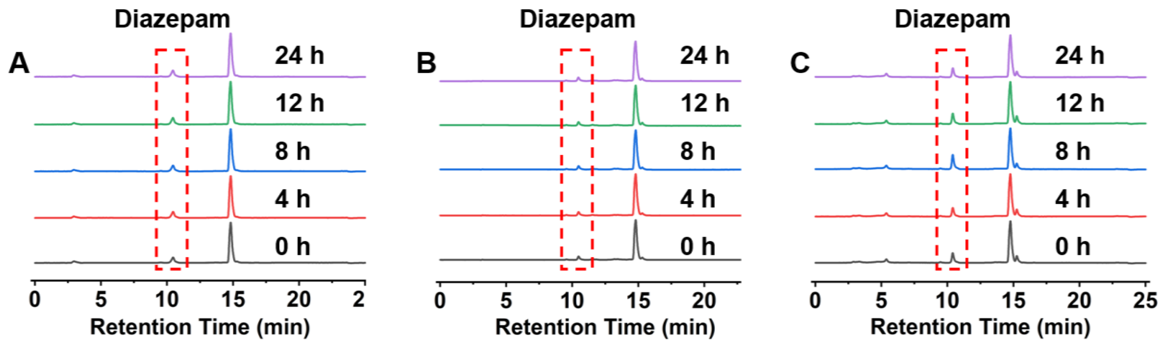

Figure S10. With increasing incubated time, monitoring the change of **Ir_2BPIFc** (10 μM) by HPLC in (A) H_2O_2, (B) GSH and (C) cell lysate, respectively, diazepam was used as an internal standard.

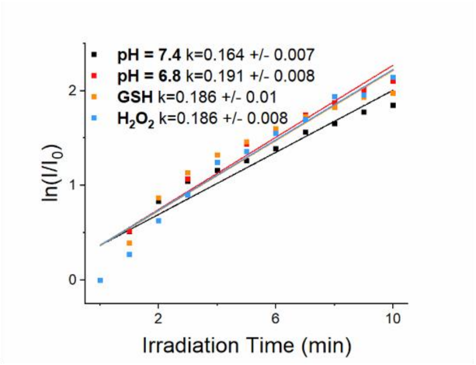

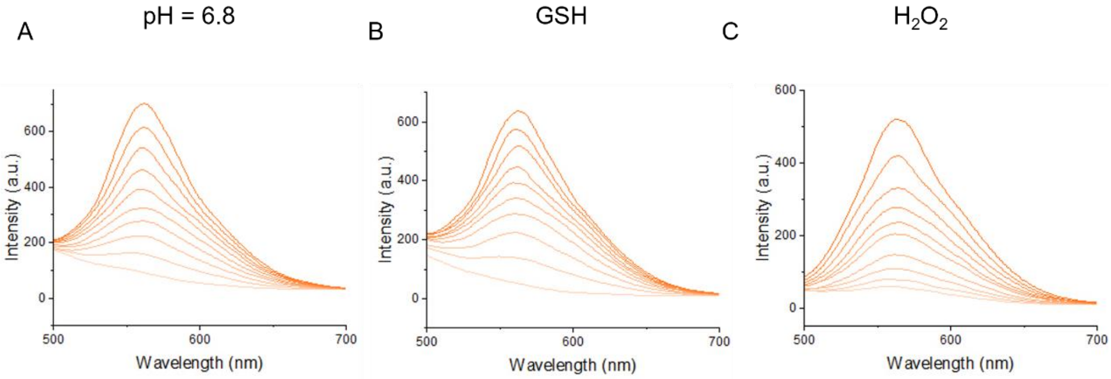

Figure S11. The emission recovery by the photolysis of Ir_2BPIFc (10 μM) in (A) Tris-HCl buffer (pH = 6.8), (B) 10 mM GSH solution and (C) 1 mM H_2O_2 solution upon irradiation at 405 nm (20 mW/cm^2).

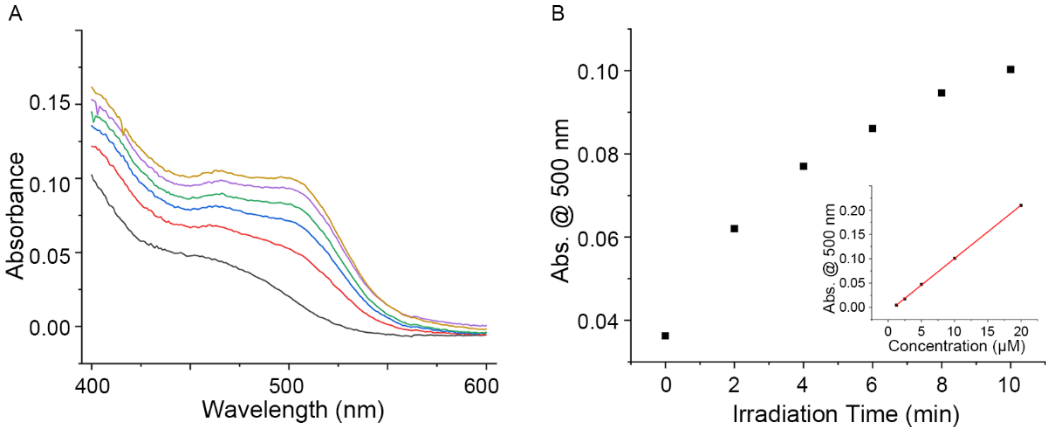

Figure S12. (A) Detection of the released Fe^{2+} from Ir_2BPIFc (10 μM) by phenanthroline (phen, 1 mM) upon irradiation at 405 nm (20 mW/cm^2, up to 10 min) in hypoxia; (B) The absorbance at 500 nm in graph A, inset: the standard curve of $Fe(phen)_3^{2+}$.

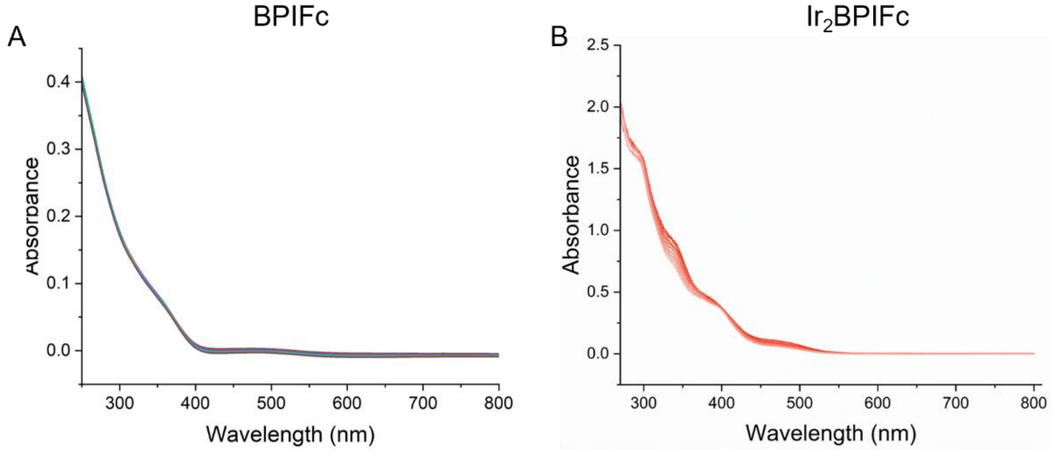

Figure S13. The change of the absorption spectra of **BPIFc** (20 μM) and $\mathbf{Ir_2BPIFc}$ (20 μM) in PBS upon irradiation at 405 nm (20 mW/cm^2).

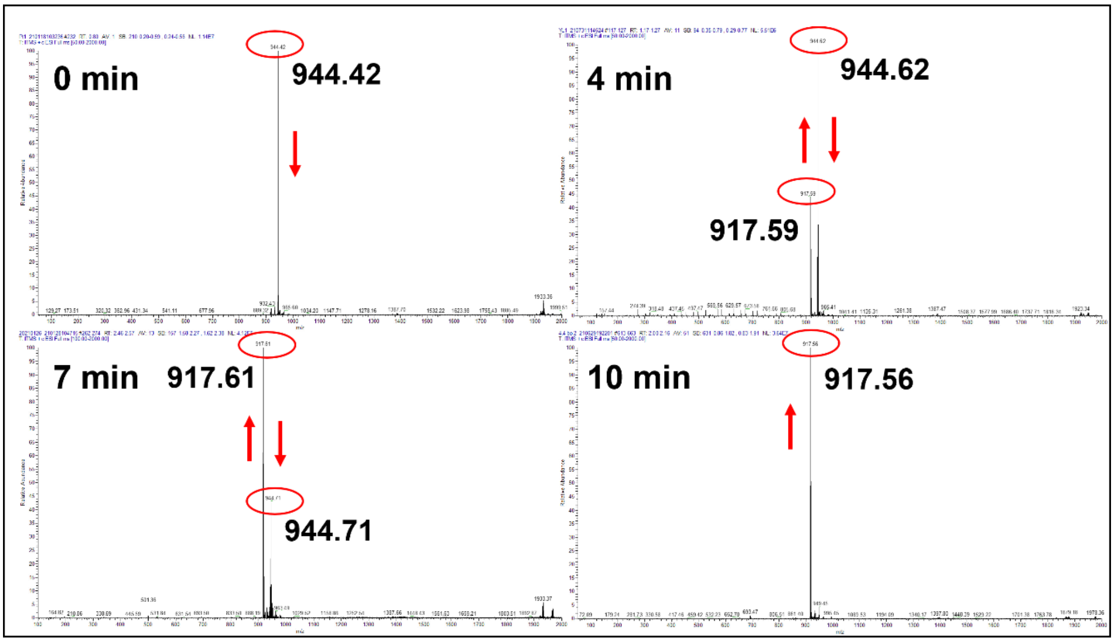

Figure S14. Time-dependent ESI-MS analyses of $\mathbf{Ir_2BPIFc}$ in acetonitrile/H_2O (1/1, v/v) upon irradiation at 405 nm (20 mW/cm^2).

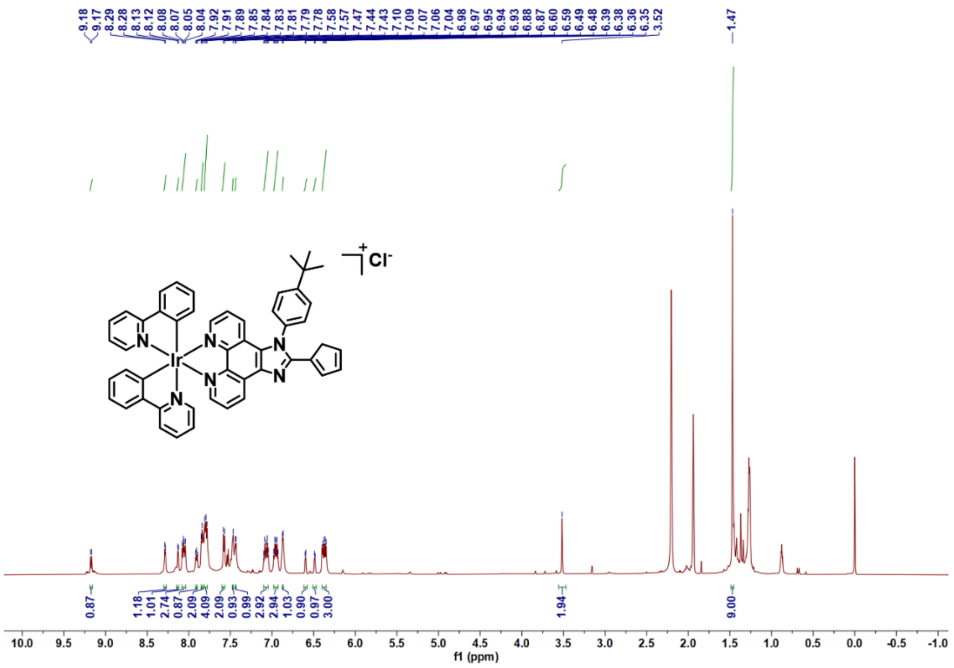

Figure S15. ^{1}H NMR spectrum of **IrPICp** in acetonitrile-*d3*.

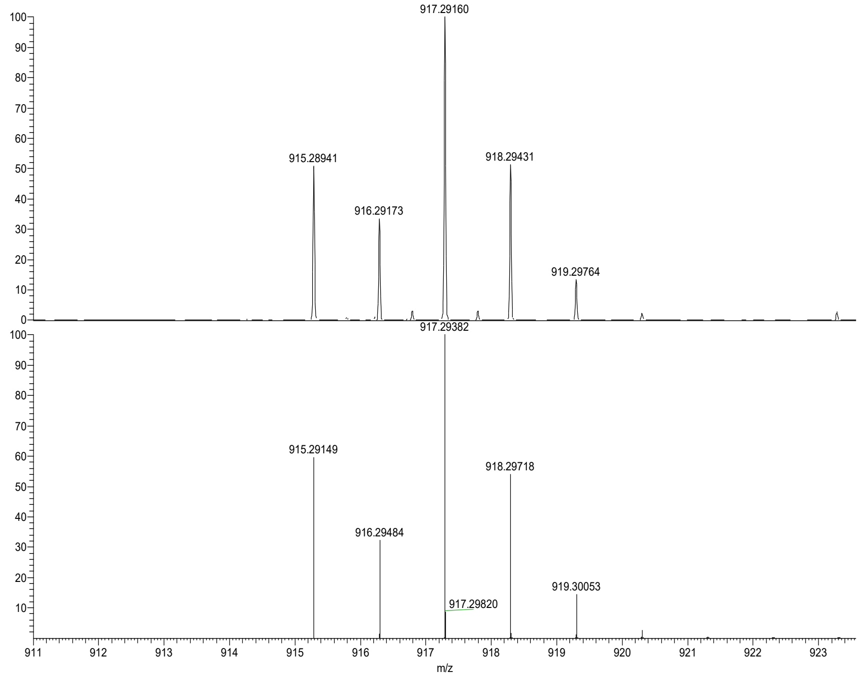

Figure S16. HR-MS characterization of **IrPICp.**

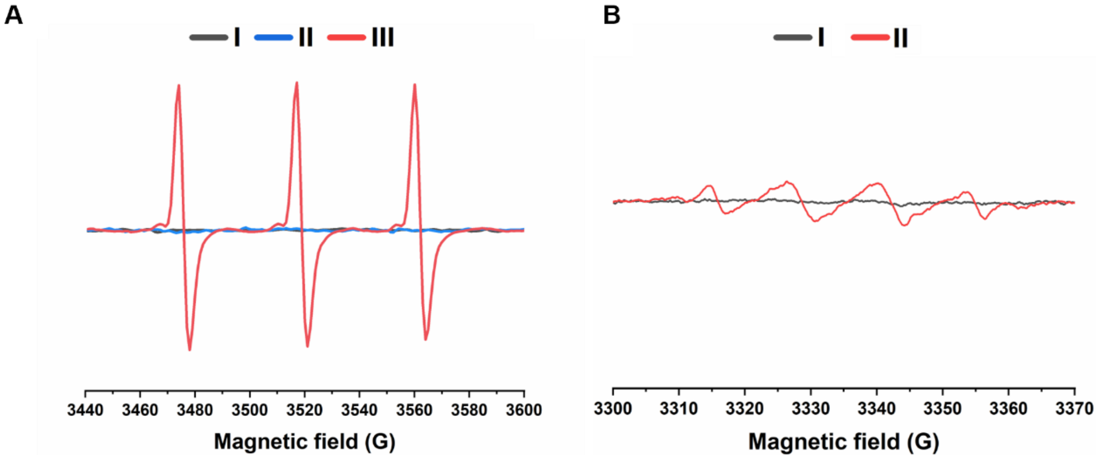

Figure S17. (A) ESR analyses of the generation of singlet oxygen by **Ir$_2$BPIFc** (10 μM**)** with/without irradiation at 405 nm (20 mW/cm^2, 5 min) in vacuous conditions (Vac.) or in ambient air (Nor.), I: **Ir$_2$BPIFc** + dark; II: **Ir$_2$BPIFc** + light in Vac.; III: **Ir$_2$BPIFc** + light in Nor.. (B) ESR analyses of the generation of hydroxyl radical by **Ir$_2$BPIFc** (10 μM) with/without irradiation at 405 nm (20 mW/cm^2, 5 min) in dry acetonitrile, I: **Ir$_2$BPIFc** + dark; II: **Ir$_2$BPIFc** + light.

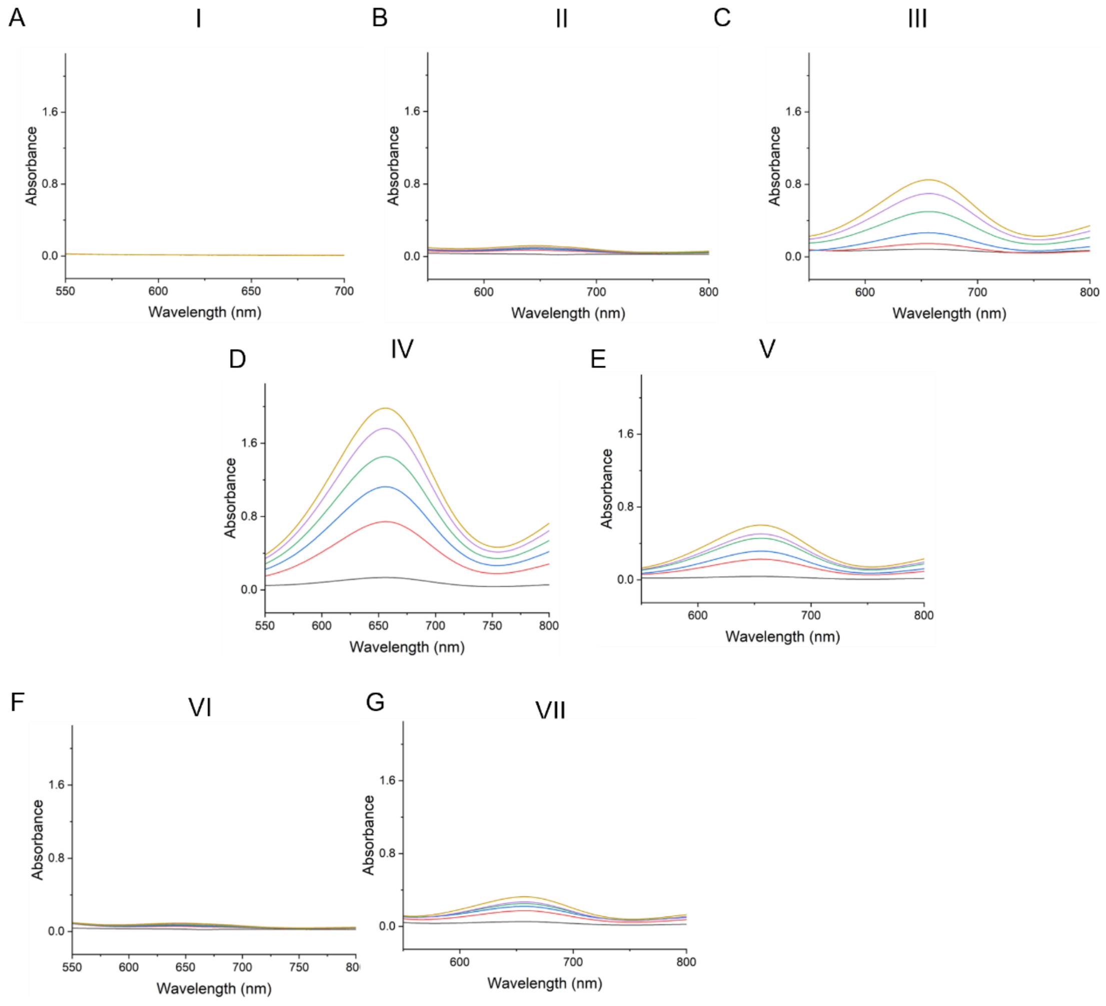

Figure S18. TMB as the probe to detect the ·OH generation of **Ir$_2$BPIFc** (10 μM) and **IrPICp** (20 μM) with/without irradiation at 405 nm (20 mW/cm^2); I: TMB + light; II: **Ir$_2$BPIFc** + dark; III: **Ir$_2$BPIFc** + light; IV: **Ir$_2$BPIFc** + H_2O_2 (100 μM) + light; V: **Ir$_2$BPIFc** + phen (1 mM) + H_2O_2 + light; VI: **IrPICp** + dark; VII: **IrPICp** + light.

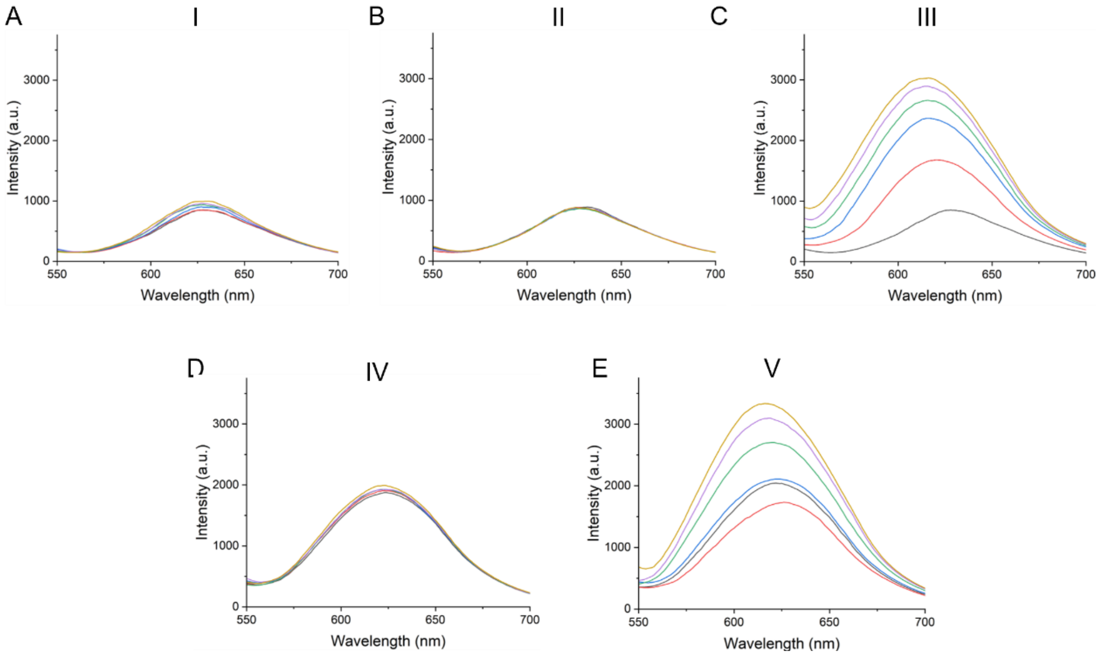

Figure S19. DHE as the probe to detect the $O_2^{\cdot-}$ generation of **Ir₂BPIFc** (10 μM) and **IrPICp** (20 μM) with/without irradiation at 405 nm (20 mW/cm^2); I: DHE + light; II: **Ir₂BPIFc** + dark; III: **Ir₂BPIFc** + light; IV: **IrPICp** + dark; V: **IrPICp** + light.

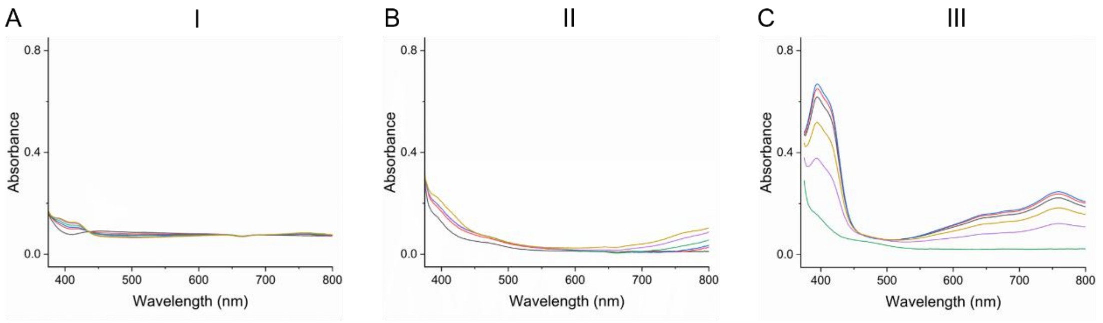

Figure S20. ABTS as the probe to detect the organic radical generation of **Ir₂BPIFc** (10 μM); I: dioxane + light; II: **Ir₂BPIFc** + dark; III: **Ir₂BPIFc** + light.

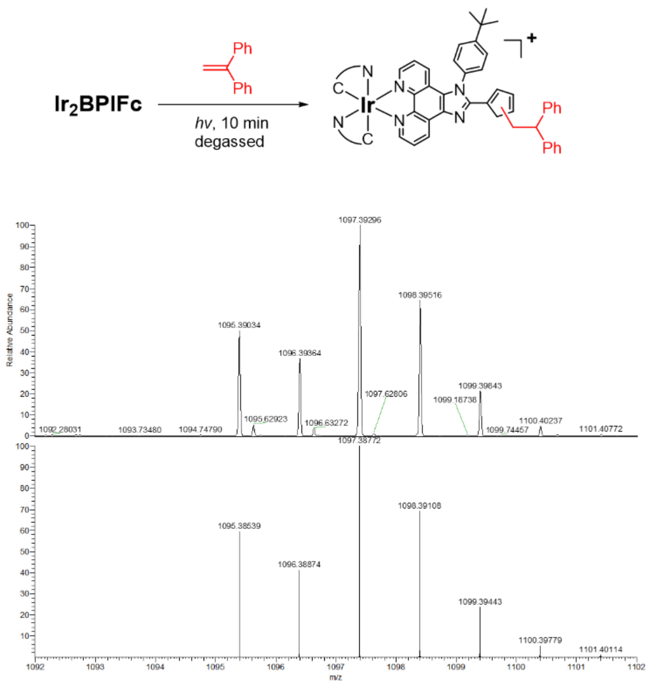

Figure S21. Diphenylethene as the probe to capture the organic radical generation of **Ir_2BPIFc** (10 μM) pon irradiation at 405 nm (20 mW/cm^2). The resulting mixture was examined by HRMS.

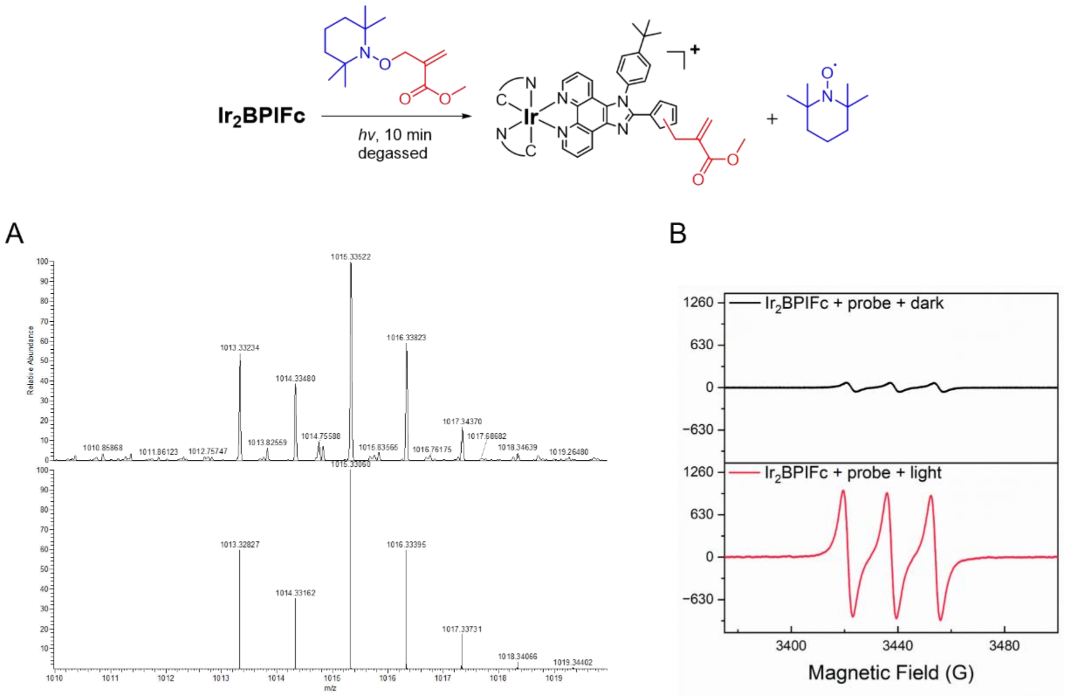

Figure S22. A synthesized probe to capture the organic radical generation of **Ir₂BPIFc** (10 μM) upon irradiation at 405 nm (20 mW/cm^2). The resulting mixture was examined by HRMS and EPR.

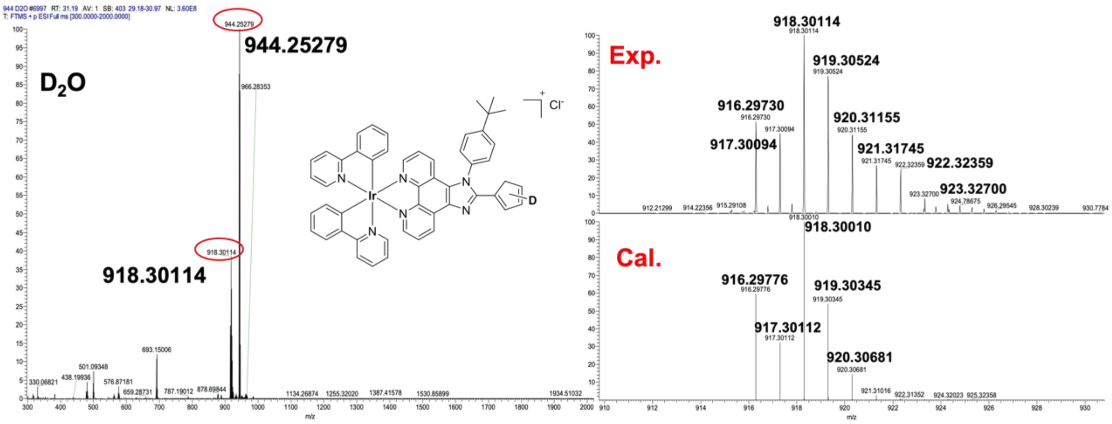

Figure S23. The HRMS of **Ir_2BPIFc** in acetonitrile/D_2O (1/1, v/v) upon irradiation at 405 nm (20 mW/cm^2).

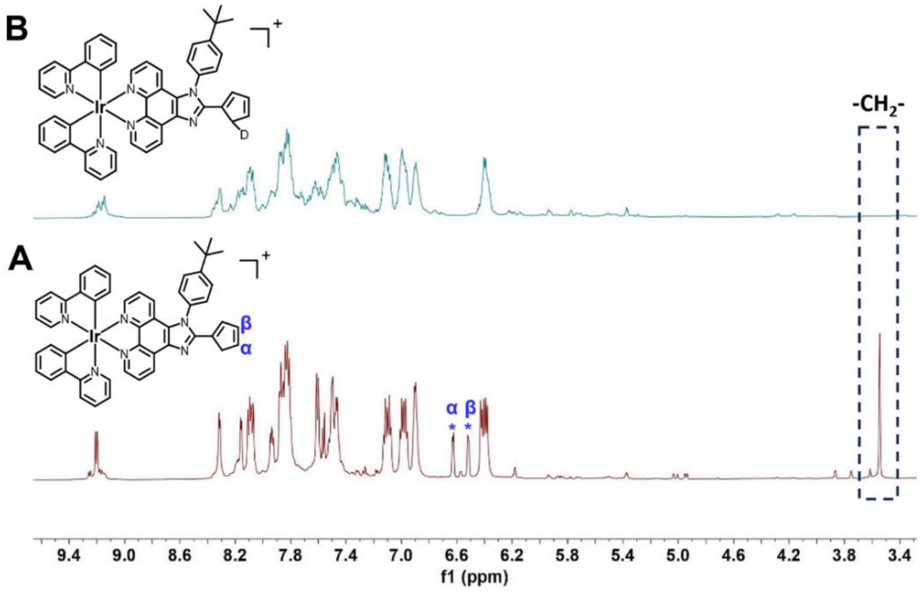

Figure S24. ^{1}H NMR spectra in acetonitrile-*d3* of the final product of **Ir_2BPIFc** upon irradiation at 405 nm (20 mW/cm^2, 10 min) in (A) acetonitrile/H_2O (1/1, v/v) or (B) acetonitrile/D_2O (1/1, v/v) in vacuous conditions.

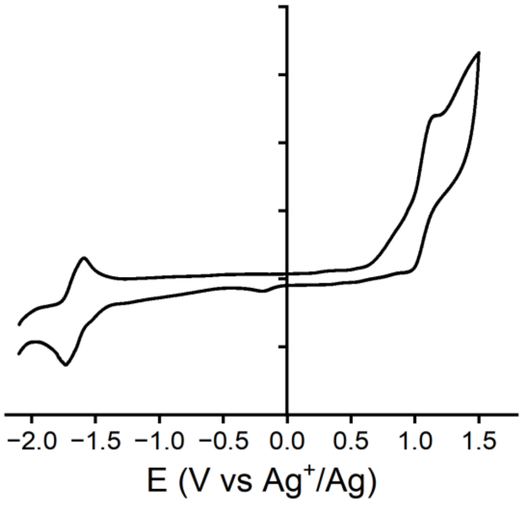

Figure S25. The cyclic voltammogram of **Ir_2BPIFc** in DCM. The scan rate for CV is 100 mV/s.

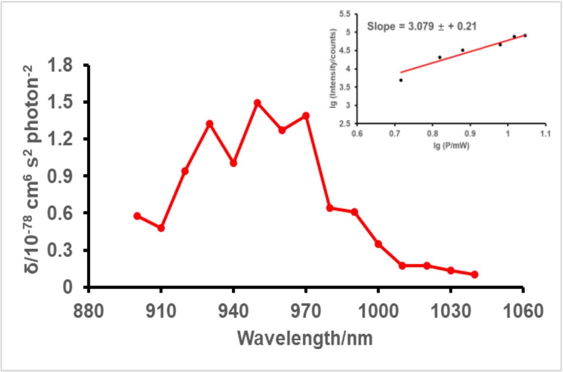

Figure S26. Three-photon absorption cross-sections of **Ir_2BPIFc** at different excitation wavelengths. Insert graph: the logarithmic plots of the power dependence of relative luminescence intensity of **Ir_2BPIFc** as a function of pump power at an excitation wavelength of 970 nm, respectively. The solid lines are the best-fit straight lines with gradient k = 3.08 ± 0.21.

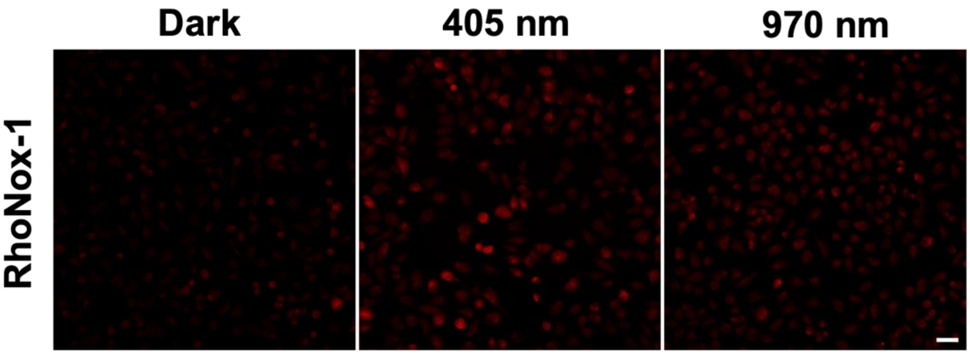

Figure S27. Fluorescent imaging probe RhoNox-1 for the detection of Fe^{2+} in **Ir_2BPIFc** (2 μM) treated A375 cells upon irradiation at 405 and 970 nm.

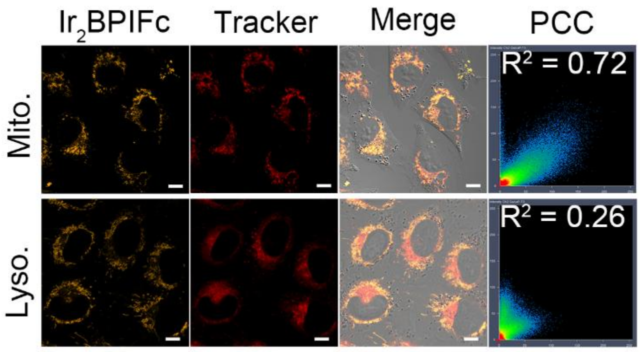

Figure S28. The subcellular localization of **Ir_2BPIFc** in A375 cells, Ir_2BPIFc: $\lambda_{ex/em}$ = 970/590 ± 10 nm, MTDR: $\lambda_{ex/em}$ = 633/670 ± 10 nm, LTDR: $\lambda_{ex/em}$ = 633/656 ± 10 nm, scale bar: 10 μm.

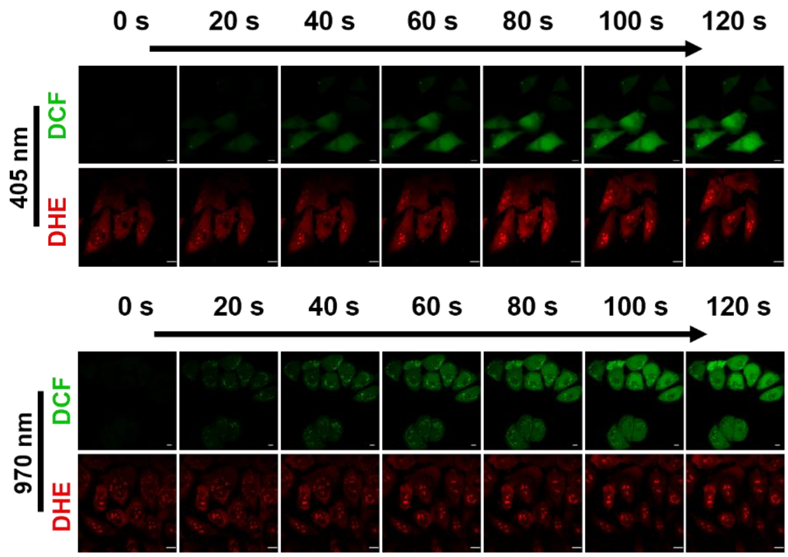

Figure S29. Total ROS generation (upper group) and $O_2^{\cdot-}$ generation (lower group) of the photoactivation of **Ir₂BPIFc** (2 µM) in A375 cells upon irradiation with 405 nm (1PE) or 970 nm (3PE) in hypoxia (2% O_2). DCF: $\lambda_{ex/em}$ = 488/525 ± 10 nm, DHE: $\lambda_{ex/em}$ = 561/625 ± 10 nm, scale bar: 10 µm.

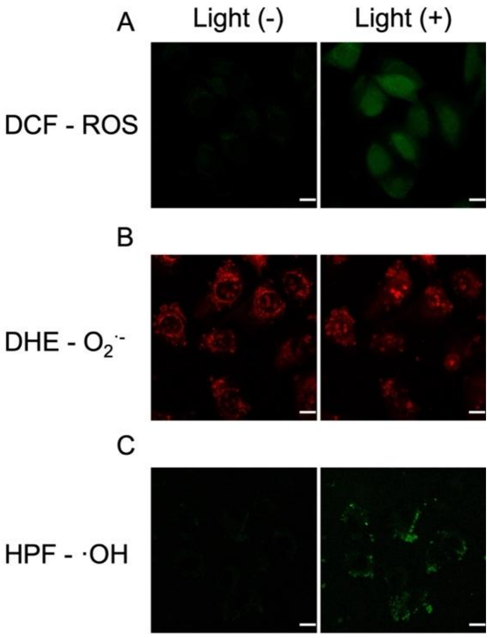

Figure S30. (A) Total ROS generation, (B) $O_2^{\cdot-}$ generation and (C) ·OH generation of **IrPICp** (5 µM) in A375 cells upon irradiation with 970 nm (3PE) in hypoxia (2% O_2). DCF: $\lambda_{ex/em}$ = 488/525 ± 10 nm, DHE: $\lambda_{ex/em}$ = 561/625 ± 10 nm, HPF: $\lambda_{ex/em}$ = 488/525 ± 10 nm, scale bar: 10 µm.

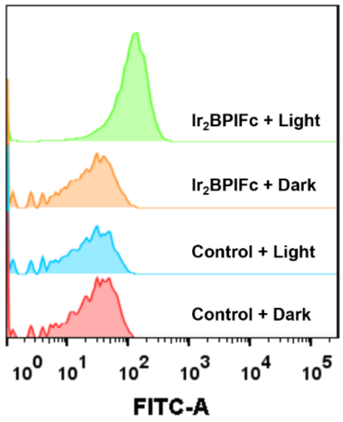

Figure S31. Lipid peroxidation generation detection by flow cytometry, the cells were incubated with **Ir$_2$BPIFc** (2 μM) for 6 h then irradiated at 405 nm (20 mW/cm^2, 10 min) in hypoxic conditions (2% O_2), then stained with C11-BODIPY (1.0 μM) for 30 min before detection.

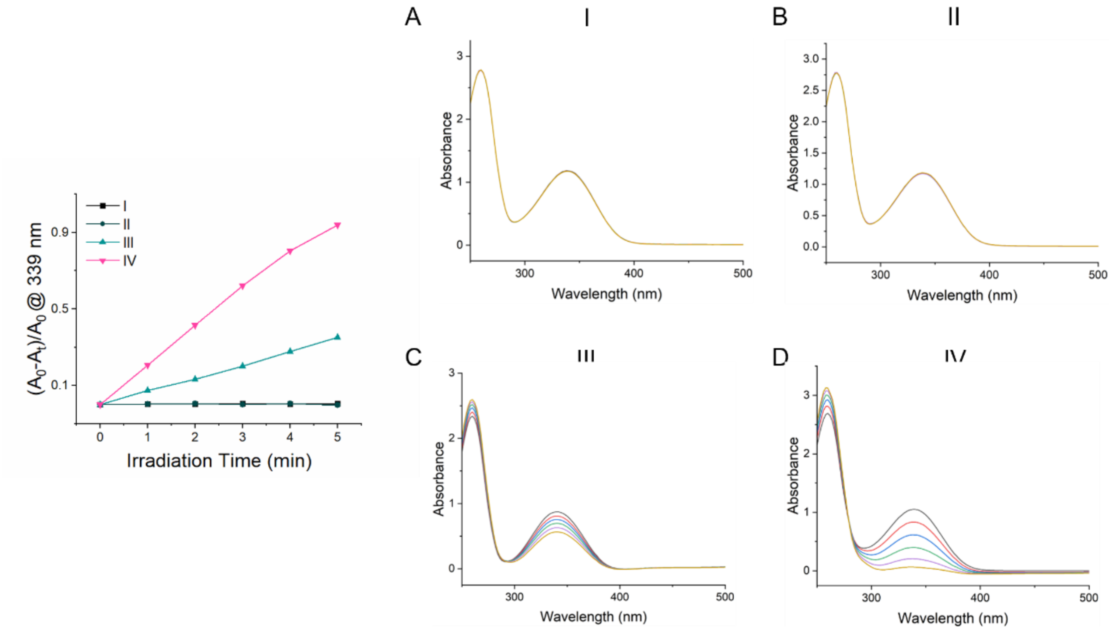

Figure S32. Changes of the absorption spectra of NADH upon the photolysis of **Ir_2BPIFc** (10 µM); I: NADH+ dark; II: **Ir_2BPIFc** + dark; III: **Ir_2BPIFc** + TEMPO + light; IV: **Ir_2BPIFc** + light.

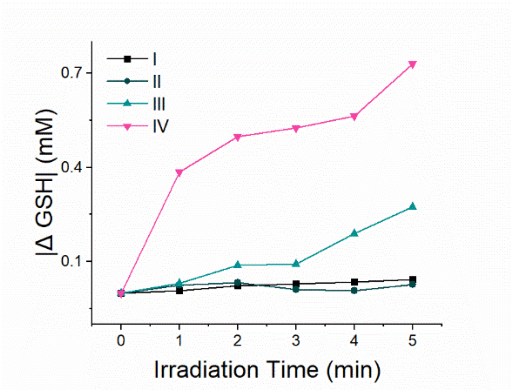

Figure S33. DTNB as the probe to detect the oxidation of GSH upon the photolysis of **Ir_2BPIFc** (10 µM); I: DTNB + dark; II: **Ir_2BPIFc** + dark; III: **Ir_2BPIFc** + light + phen (1 Mm); IV: **Ir_2BPIFc** + light.

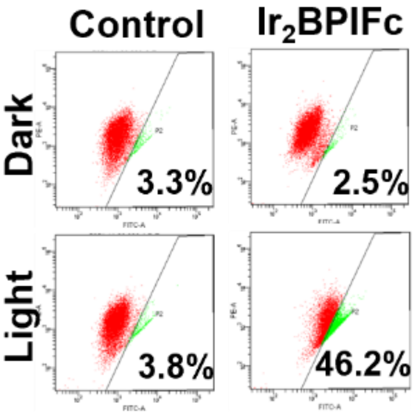

Figure S34. Mitochondria membrane potential detection by flow cytometry, the cells were incubated with **Ir_2BPIFc** (2 μM) for 6 h then irradiated at 405 nm (20 mW/cm^2, 10 min) in hypoxic conditions (2% O_2) or kept in the dark, then stained with JC-1 assay kit before detection.

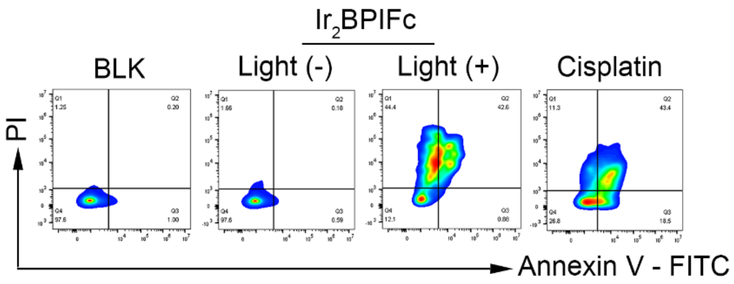

Figure S35. Flow cytometry analysis of A375 cell viability by Annexin V-FITC/PI assay upon irradiation with 405 nm (1PE) in hypoxia (2% O_2), cisplatin (30 μm) as a typical apoptosis inducer was employed.

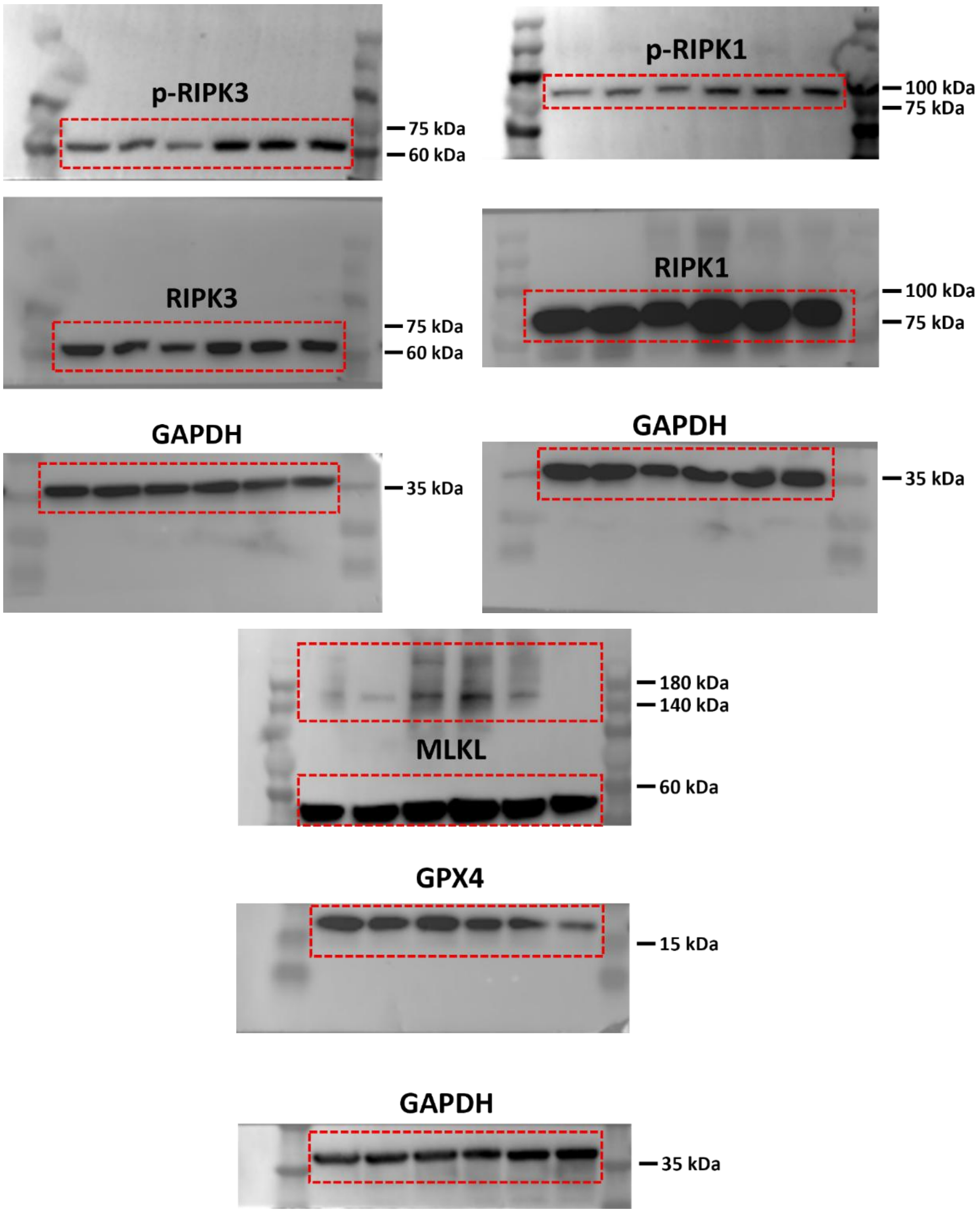

Figure S36. The uncropped western blotting image of Figure 3H.

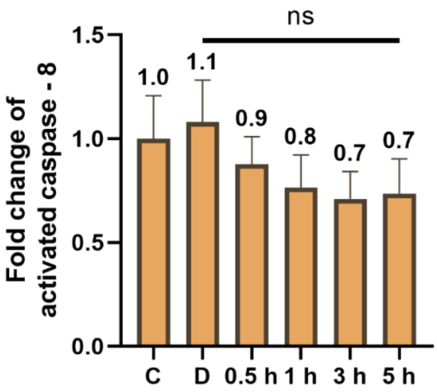

Figure S37. Time-dependent fold change of activated caspase-8 in A375 cells after the photoactivation of **Ir_2BPIFc** (2 μM) in hypoxia (2% O_2) upon irradiation at 405 nm (1PE).

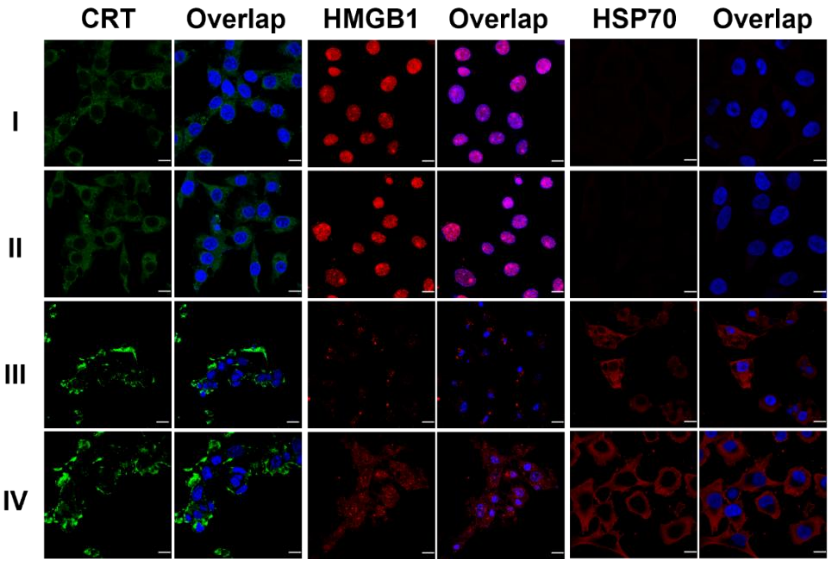

Figure S38. Evaluation for hallmarks of ICD in B16F10 cells at 12 h after treatments. Confocal images of the immunofluorescence stained with the calreticulin (CRT) specific antibody, the nuclear high-mobility group box 1 (HMGB1) specific antibody and the heatshock protein 70 (HSP70) specific antibody. Hoechst 33342 was employed as the nuclear probe. Cells were incubated with **Ir$_2$BPIFc** (2 µM) or hypericin (1 µM) for 6 h in the dark. Scale bar: 10 µm.

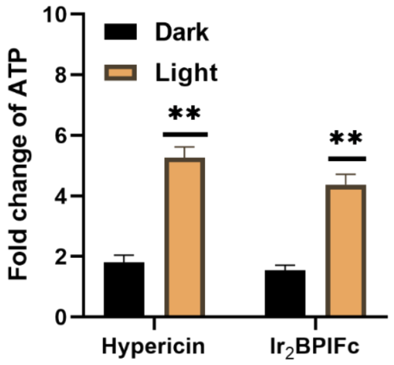

Figure S39. Fold change of extracellular ATP in B16F10 cells detected at 12 h after treated with **Ir$_2$BPIFc** (2 µM) or hypericin (1 µM) upon irradiation with 405 nm (1PE)**.**

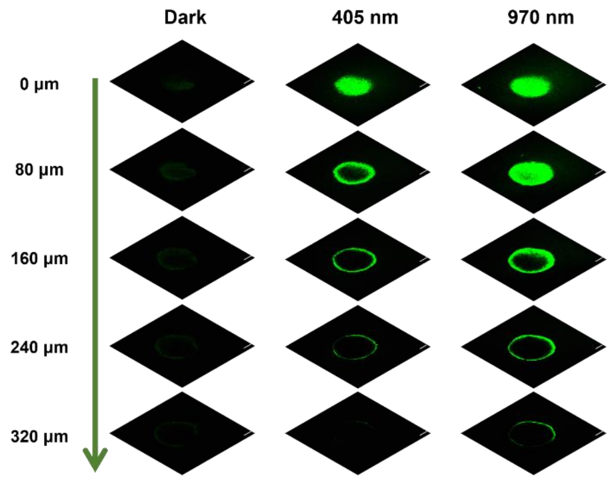

Figure S40. Total ROS generation of the photoactivation of **Ir_2BPIFc** (2 µM) in A375 cell pheroids upon irradiation with 405 nm (1PE) or 970 nm (3PE) in hypoxia (2% O2). DCF: $\lambda_{ex/em}$ = 740 (2PE)/525 ± 10 nm, scale bar: 100 µm.

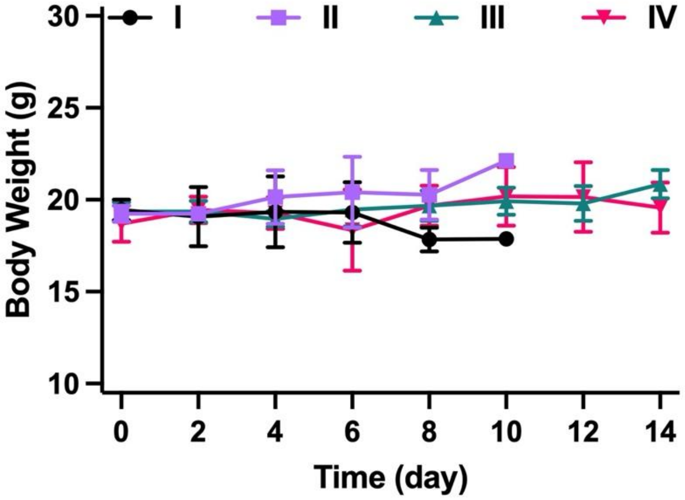

Figure S41. Changes in the body weight of the mice during treatments.

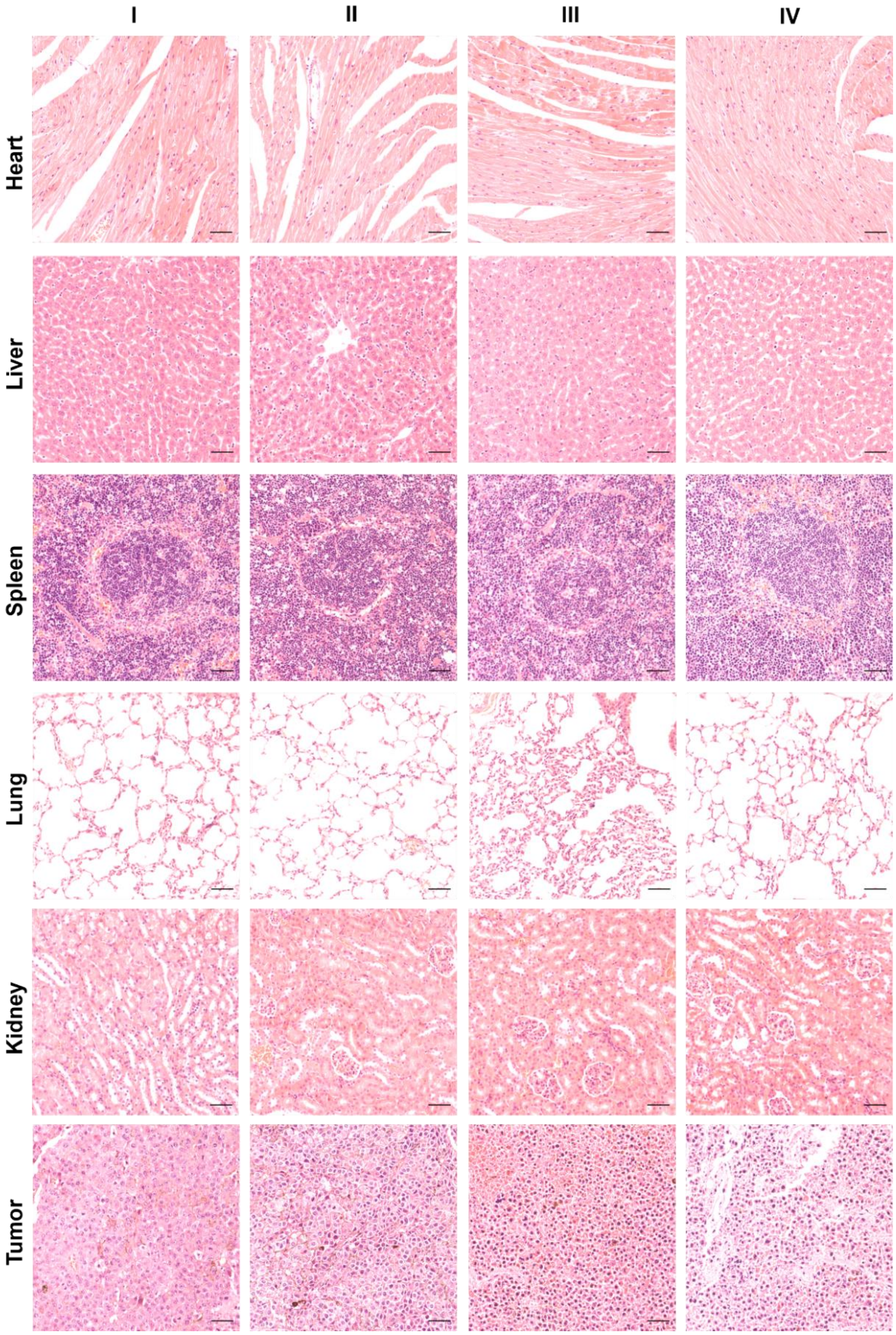

Figure S42. Histological examination of the main organs and tumors of the mice on day 14. Slices stained by hematoxylin-eosin (H&E). Scale bar: 50 μm.

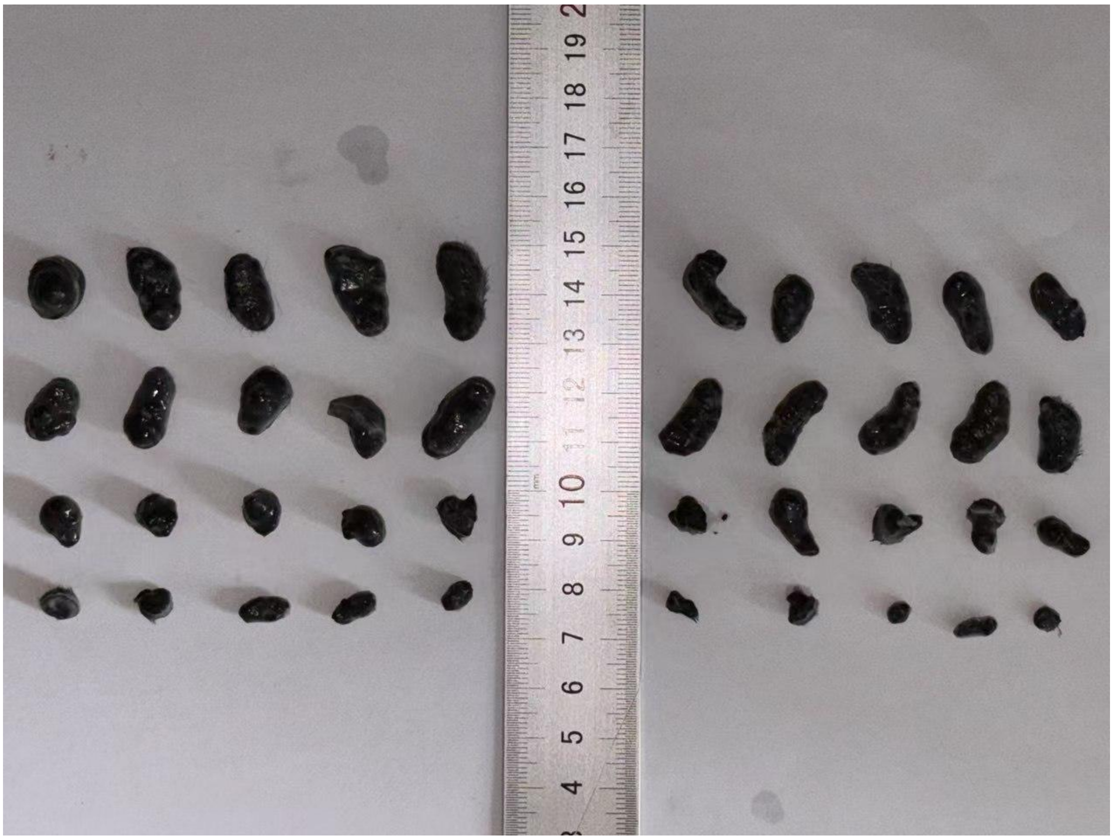

Figure S43. Volume monitoring of left: primary tumors and right: distant tumors of each individual mouse upon different treatments.

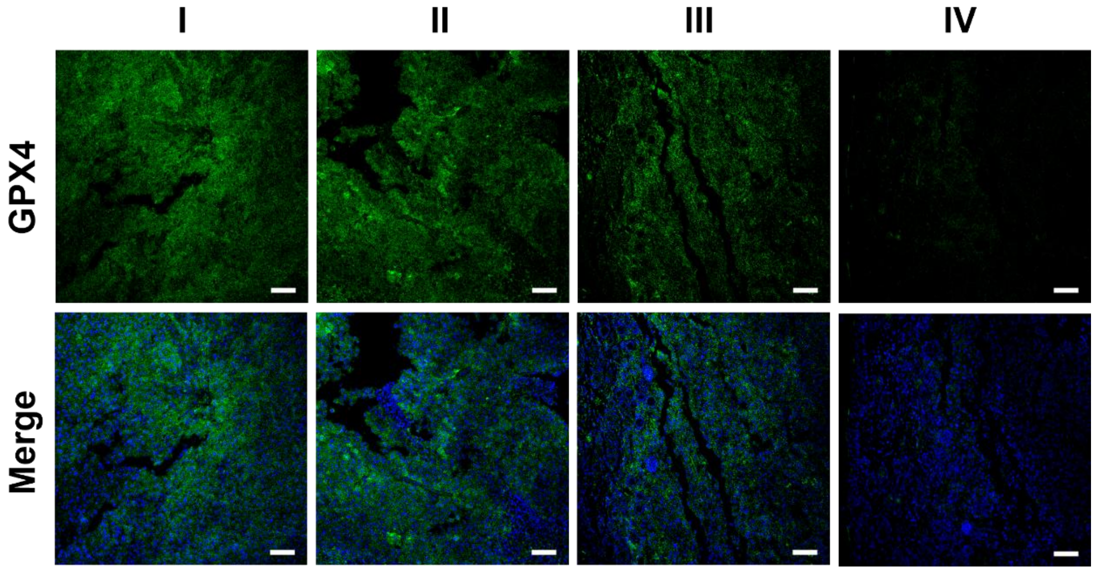

Figure S44. Evaluation of the expression of Glutathione Peroxidase 4 (GPX4) in primary tumors on day 14. Confocal images of immunofluorescene stained with GPX4 specific antibody. DAPI was employed as the nuclear probe. Scale bar: 100 µm.

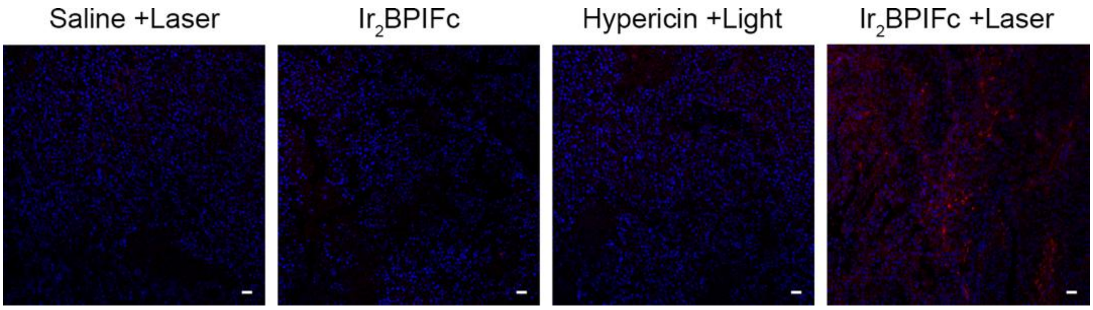

Figure S45. Confocal images of immunofluorescene in primary tumors on day 14 stained with p-MLKL specific antibody. DAPI was employed as the nuclear probe. Scale bar: 50 µm.

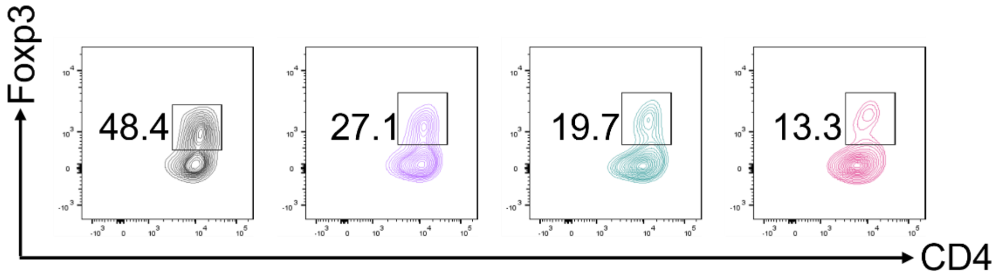

Figure S46. Representative flow cytometry plots indicate the proportions of regulatory T cells in distant tumors on day 14.

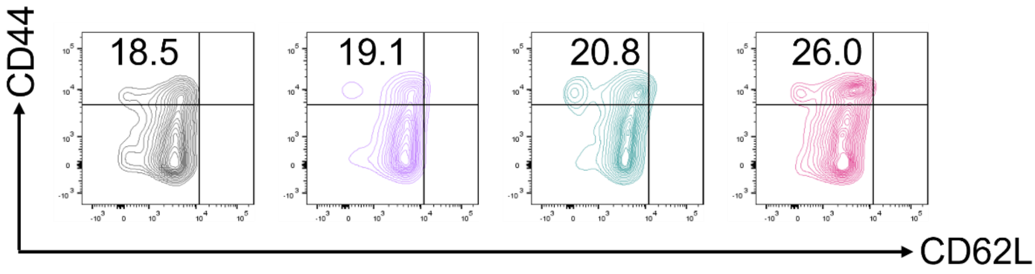

Figure S47. Representative flow cytometry plots indicate the proportions of effector memory T cells in distant tumors on day 14.

Table S1. (Photo)cytotoxicity (µM) towards different cells for 24 h in hypoxia (2% O_2).

	Ir_2**BPIFc**			**IrPICp**		
	**Dark**	**Light[a]**	**PI[b]**	**Dark**	**Light[a]**	**PI[b]**
**A375**	16.32 ± 0.67	2.06 ± 0.22	7.92	17.26 ± 0.16	5.19 ± 0.73	3.32
**B16F10**	28.90 ± 1.07	2.34 ± 0.46	12.35	n.a.	n.a.	n.a.

[a] Irradiated by LED at 405 nm (20 mW/cm^2, 10 min).
[b] PI (photocytotoxicity index) is the ratio of dark-to-light toxicity.

Table S2. Computational detail of each species.

	**Gas phase**	
**Species**	***G* (hartree)**	**D*G* (eV)**
**$1S_0$**	-4846.019944	
**$1T_1$-1**	-4845.935748	0.02
**$1T_1$-2**	-4845.956615	0.27
**$1T_1$-3**	-4846.011849	-0.24
**$2T_1$**	-2361.092365	-0.19
**$3P_1$**	-3405.658792	
**Cl^-**	-460.315733	-0.26

Table S3. Routine blood indexes of healthy C57BL/6 mice after different treatments. The data are presented as mean ± standard deviation (n = 3).

Item	Control	Hypericin@PEG	Ir_2BPIFc@PEG
WBC (10^9/L)	6.05 ± 1.34	6.25 ± 2.05	5.85 ± 0.07
RBC (10^12/L)	7.23 ± 0.75	8.97 ± 3.11	7.77 ± 0.21
HGB (g/L)	78.5 ± 16.26	133 ± 43.84	121.5 ± 2.12
HCT (%)	28.9 ± 6.36	43.85 ± 11.24	42.3 ± 0.98
MCV (fL)	55 ± 4.24	49.7 ± 4.66	54.4 ± 0.21
MCH (pg)	14.9 ± 0.98	14.8 ± 0.28	15.6 ± 0.14
MCHC (g/L)	271.5 ± 3.54	330 ± 22.62	287 ± 1.41
PLT (10^9/L)	703.5 ± 78.89	741.5 ± 35.06	841 ± 101.93
RDW-CV (%)	16.2 ± 0.98	15.85 ± 0.35	14.9 ± 0.42
PDW (fL)	17.05 ± 0.21	16.6 ± 0.14	17.3 ± 0.37
MPV (fL)	7.7 ± 0.28	6.45 ± 1.06	7.2 ± 0.75
LYMPH (10^9/L)	3.9 ± 2.68	4.8 ± 1.97	4.95 ± 0.07
MONO (10^9/L)	0.98 ± 0.09	0.2 ± 0.02	0.2 ± 0.04
LYMPH (%)	56.9 ± 12.31	74.7 ± 7.49	84.5 ± 1.84
MONO (%)	9.45 ± 5.16	3.9 ± 1.27	3.35 ± 0.49
ALT (U/L)	45.17 ± 2.66	48.83 ± 7.95	50.42 ± 1.15
AST (U/L)	143.98 ± 68.55	195.38 ± 10.31	194.02 ± 31.8
TBIL (μmol/L)	16.95 ± 9.40	9.48 ± 0.12	15.77 ± 6.12
TP (g/L)	39.83 ± 6.85	42.26 ± 2.24	47. 33 ± 1.83
ALB (g/L)	25.19 ± 3.33	27.93 ± 3.25	32.8 ± 1.16
GLOB (g/L)	14.62 ± 3.51	14.32 ± 1.01	14.53 ± 0.67
CREA (μmol/L)	23.93 ± 2.73	13.79 ± 4.62	17.3 ± 0.95
GLU (mmol/L)	8.95 ± 1.03	7.60 ± 1.56	6.82 ± 0.43
GGT (U/L)	5.55 ± 1.03	6.85 ± 0.50	5.48 ± 0.05

1S$_0$

Electronic Energy *E*= -4847.4241

Thermal correction to Gibbs Free Energy= 1.404175

Center Number	Atomic Number	Atomic Type	Coordinates (Angstroms) X	Y	Z
1	6	0	2.946470	-1.281646	1.288934
2	6	0	-2.646514	-1.848021	-0.993923
3	6	0	-8.256918	-3.097045	0.121088
4	6	0	-6.896418	-3.092159	-0.117934
5	6	0	-6.232636	-1.866467	-0.350631
6	6	0	-7.021189	-0.678015	-0.330217
7	6	0	-8.960684	-1.886570	0.131718
8	6	0	-4.834315	-1.689699	-0.614312
9	6	0	-6.431937	0.624339	-0.567128
10	6	0	-5.049930	0.756038	-0.844065
11	6	0	-4.265524	-0.442297	-0.862709
12	6	0	-4.531967	2.045533	-1.076274
13	1	0	-3.476484	2.171234	-1.291106
14	6	0	-5.383812	3.131905	-1.024177
15	6	0	-6.742765	2.925093	-0.743028
16	1	0	-8.788471	-4.024703	0.302105
17	1	0	-6.347392	-4.024104	-0.128462
18	1	0	-10.027440	-1.857297	0.322206
19	1	0	-5.023053	4.140197	-1.195205
20	1	0	-7.437420	3.756403	-0.698012
21	6	0	6.432295	3.213347	-0.104953
22	6	0	5.397681	2.355717	0.214869
23	6	0	5.665072	0.982147	0.413558
24	6	0	7.016266	0.548514	0.268179
25	6	0	7.733625	2.710988	-0.226955
26	6	0	4.711388	-0.033329	0.754876
27	6	0	7.388614	-0.839535	0.454064
28	6	0	6.417671	-1.813170	0.791172
29	6	0	5.064584	-1.368178	0.941447
30	6	0	6.835430	-3.148562	0.955377
31	1	0	6.109227	-3.911066	1.214684
32	6	0	8.169267	-3.462465	0.780203
33	6	0	9.075249	-2.444495	0.447031
34	1	0	6.252029	4.271171	-0.261096
35	1	0	4.390727	2.738680	0.313000
36	1	0	8.567088	3.359505	-0.471831
37	1	0	8.530092	-4.478600	0.894947

38	1	0	10.128124	-2.658428	0.301484
39	7	0	-2.922924	-0.552529	-1.101005
40	7	0	-3.779960	-2.590921	-0.702897
41	7	0	3.341307	0.012124	0.983079
42	7	0	3.973662	-2.124510	1.272291
43	7	0	-7.254403	1.712817	-0.520614
44	7	0	-8.363126	-0.714384	-0.088126
45	7	0	8.017341	1.420006	-0.047474
46	7	0	8.702001	-1.173023	0.290856
47	6	0	-8.238234	1.124247	4.148941
48	6	0	-8.558332	2.412865	4.583016
49	6	0	-9.100900	3.317525	3.678829
50	6	0	-9.320098	2.936426	2.348388
51	6	0	-8.469341	0.795548	2.820910
52	6	0	-9.899838	3.782680	1.300553
53	6	0	-10.292334	5.115255	1.514582
54	6	0	-10.837800	5.858517	0.470766
55	6	0	-10.991135	5.268844	-0.789433
56	6	0	-10.601327	3.942922	-1.005452
57	1	0	-7.818120	0.383119	4.819621
58	1	0	-8.242066	-0.191562	2.437259
59	1	0	-11.418112	5.844385	-1.607521
60	1	0	-10.733919	3.513446	-1.995475
61	6	0	-13.232843	-0.123122	1.480083
62	6	0	-13.902664	-0.456784	0.297186
63	6	0	-13.265104	-0.274231	-0.927516
64	6	0	-11.958457	0.241736	-0.971408
65	6	0	-11.932344	0.390603	1.439429
66	6	0	-11.220793	0.458971	-2.220094
67	6	0	-11.711369	0.223191	-3.511262
68	6	0	-10.913581	0.483290	-4.618670
69	6	0	-9.622740	0.981725	-4.426905
70	6	0	-9.181203	1.196853	-3.129271
71	1	0	-13.726712	-0.262677	2.438975
72	1	0	-11.439129	0.640102	2.375755
73	1	0	-8.965920	1.203368	-5.260569
74	1	0	-8.190867	1.586849	-2.928433
75	7	0	-8.989624	1.673120	1.939242
76	7	0	-9.951200	0.941087	-2.052312
77	6	0	9.139217	-0.574280	-2.916574
78	6	0	10.353254	1.439278	-2.981669
79	6	0	9.011153	-0.601880	-4.297800
80	1	0	8.723045	-1.358076	-2.295435
81	6	0	10.248832	1.457102	-4.378954

82	6	0	9.576902	0.436906	-5.041029
83	1	0	8.481365	-1.420798	-4.771302
84	6	0	11.042398	2.439938	-2.160447
85	6	0	11.691534	3.123374	0.063702
86	6	0	11.666679	3.580165	-2.695083
87	6	0	12.313021	4.256630	-0.471255
88	1	0	11.716730	2.971774	1.140122
89	6	0	12.301980	4.488298	-1.851666
90	1	0	12.808929	4.962029	0.191572
91	7	0	9.786692	0.416607	-2.270950
92	6	0	12.369831	-0.628770	1.131659
93	6	0	12.449825	-1.124727	-1.231361
94	6	0	13.565888	-1.341692	1.323545
95	6	0	13.639504	-1.834376	-1.037005
96	1	0	12.040108	-1.055977	-2.236122
97	6	0	14.200719	-1.944739	0.240539
98	1	0	14.132141	-2.302006	-1.886384
99	6	0	9.752820	1.331489	2.740685
100	6	0	11.643342	0.032845	2.220398
101	6	0	10.101464	1.394491	4.082181
102	1	0	8.863263	1.823014	2.366009
103	6	0	12.034241	0.062476	3.565735
104	6	0	11.263857	0.742564	4.500946
105	1	0	9.472188	1.942098	4.774794
106	7	0	10.494547	0.666624	1.831979
107	1	0	11.567013	0.767171	5.543002
108	1	0	12.941543	-0.445219	3.871526
109	1	0	14.004129	-1.428590	2.313885
110	1	0	15.125370	-2.494970	0.389551
111	1	0	11.657747	3.763922	-3.765841
112	1	0	10.698592	2.267712	-4.940296
113	1	0	9.497025	0.449545	-6.123595
114	1	0	-8.389887	2.706998	5.614332
115	1	0	-9.359717	4.320250	3.998398
116	1	0	-10.174383	5.575252	2.491743
117	1	0	-11.141582	6.888128	0.636811
118	1	0	-13.786390	-0.536214	-1.844103
119	1	0	-14.912635	-0.855216	0.330712
120	1	0	-12.717475	-0.158349	-3.640698
121	1	0	-11.293727	0.302840	-5.619386
122	77	0	10.029329	0.517113	-0.198999
123	77	0	-9.364691	1.254605	-0.072577
124	6	0	-10.044770	3.166119	0.026034
125	6	0	-11.259215	0.584085	0.220184

126	6	0	11.038508	2.188832	-0.759338
127	6	0	11.782063	-0.507313	-0.158994
128	1	0	12.784595	5.369082	-2.265479
129	6	0	-3.843531	-4.008984	-0.468143
130	6	0	-4.040755	-4.886063	-1.532299
131	6	0	-3.708583	-4.500122	0.833425
132	6	0	-4.097626	-6.261361	-1.291721
133	1	0	-4.147074	-4.500146	-2.541438
134	6	0	-3.769085	-5.873386	1.057095
135	1	0	-3.559507	-3.811409	1.659519
136	6	0	-3.963170	-6.786837	0.002825
137	1	0	-4.250835	-6.921295	-2.137526
138	1	0	-3.663381	-6.235711	2.074990
139	6	0	-4.024886	-8.294773	0.300191
140	6	0	-5.200996	-8.576377	1.264087
141	1	0	-5.252755	-9.648383	1.489543
142	1	0	-5.088405	-8.040158	2.212447
143	1	0	-6.156427	-8.277482	0.816690
144	6	0	-4.231208	-9.134498	-0.972994
145	1	0	-3.412777	-8.998961	-1.689418
146	1	0	-4.266063	-10.196755	-0.706208
147	1	0	-5.172499	-8.888315	-1.477667
148	6	0	-2.702308	-8.739746	0.966491
149	1	0	-2.736070	-9.813534	1.186763
150	1	0	-1.847425	-8.556360	0.304560
151	1	0	-2.521790	-8.210999	1.908494
152	6	0	2.491514	1.168305	0.876116
153	6	0	2.238686	1.955275	1.997661
154	6	0	1.918428	1.494726	-0.355826
155	6	0	1.397885	3.065812	1.886917
156	1	0	2.689292	1.700673	2.952028
157	6	0	1.083891	2.605824	-0.450557
158	1	0	2.125879	0.883782	-1.228992
159	6	0	0.798461	3.414603	0.666494
160	1	0	1.215354	3.657870	2.776024
161	1	0	0.647895	2.842644	-1.416164
162	6	0	-0.140265	4.624145	0.515081
163	6	0	-1.526275	4.134375	0.032499
164	1	0	-2.206073	4.986969	-0.085627
165	1	0	-1.461270	3.620951	-0.932802
166	1	0	-1.972240	3.442612	0.756854
167	6	0	0.446641	5.602012	-0.529242
168	1	0	-0.217155	6.466969	-0.647125
169	1	0	1.430225	5.970239	-0.214058

170	1	0	0.559684	5.131564	-1.511851
171	6	0	-0.330587	5.386716	1.838415
172	1	0	-0.773645	4.753244	2.615504
173	1	0	0.616263	5.785727	2.220138
174	1	0	-1.006235	6.233993	1.675893
175	6	0	0.613694	-3.681933	-1.337920
176	6	0	0.976028	-2.337226	-1.648155
177	6	0	-0.190203	-1.530392	-1.549498
178	6	0	-1.298443	-2.377086	-1.187451
179	6	0	-0.780928	-3.714577	-1.047701
180	1	0	1.283317	-4.531481	-1.298445
181	1	0	1.972867	-1.982640	-1.877773
182	1	0	-0.245225	-0.463481	-1.710404
183	1	0	-1.333131	-4.595617	-0.760422
184	6	0	1.271864	-3.085242	1.917056
185	6	0	-0.116641	-3.172075	2.210554
186	6	0	-0.675437	-1.864277	2.092869
187	6	0	0.362894	-0.961925	1.725167
188	6	0	1.590128	-1.711385	1.622262
189	1	0	1.975577	-3.905349	1.907278
190	1	0	-0.658316	-4.078574	2.447725
191	1	0	-1.718195	-1.601453	2.218339
192	1	0	0.232264	0.094248	1.548952
193	26	0	0.176754	-2.438288	0.278416

1T$_1$-1

Electronic Energy *E*= -4847.3349

Thermal correction to Gibbs Free Energy= 1.399167

Center Number	Atomic Number	Atomic Type	Coordinates (Angstroms) X	Y	Z
1	6	0	2.958969	-1.304470	1.273060
2	6	0	-2.638178	-1.872127	-1.004802
3	6	0	-8.252622	-3.091672	0.124519
4	6	0	-6.892841	-3.094066	-0.118581
5	6	0	-6.223254	-1.872005	-0.353981
6	6	0	-7.005657	-0.679419	-0.331847
7	6	0	-8.950047	-1.877503	0.136799
8	6	0	-4.824806	-1.702669	-0.621444
9	6	0	-6.410360	0.619639	-0.571254

10	6	0	-5.028570	0.743837	-0.852675
11	6	0	-4.250270	-0.458384	-0.872171
12	6	0	-4.504620	2.030338	-1.087905
13	1	0	-3.449260	2.150133	-1.306553
14	6	0	-5.350421	3.121307	-1.033710
15	6	0	-6.709466	2.921950	-0.747563
16	1	0	-8.788454	-4.016452	0.307658
17	1	0	-6.348765	-4.028898	-0.130182
18	1	0	-10.016020	-1.842534	0.330691
19	1	0	-4.984797	4.127506	-1.206789
20	1	0	-7.399488	3.756995	-0.700616
21	6	0	6.436608	3.210652	-0.181005
22	6	0	5.386168	2.332143	0.161165
23	6	0	5.660073	0.984054	0.375279
24	6	0	7.024789	0.525119	0.239221
25	6	0	7.724979	2.719431	-0.295488
26	6	0	4.721208	-0.050136	0.729909
27	6	0	7.397762	-0.818939	0.433765
28	6	0	6.442209	-1.831440	0.782049
29	6	0	5.082457	-1.379651	0.925483
30	6	0	6.866518	-3.141860	0.954783
31	1	0	6.151859	-3.914136	1.218653
32	6	0	8.233978	-3.449977	0.780918
33	6	0	9.117777	-2.437515	0.448732
34	1	0	6.247679	4.264494	-0.352804
35	1	0	4.378758	2.715000	0.254331
36	1	0	8.549455	3.375889	-0.550168
37	1	0	8.602758	-4.462563	0.902455
38	1	0	10.172206	-2.646832	0.306669
39	7	0	-2.908706	-0.575340	-1.112808
40	7	0	-3.774927	-2.609138	-0.711204
41	7	0	3.348009	-0.011682	0.958487
42	7	0	3.991390	-2.139709	1.261761
43	7	0	-7.226800	1.712566	-0.522576
44	7	0	-8.347028	-0.708594	-0.085514
45	7	0	8.037716	1.421455	-0.096068
46	7	0	8.740879	-1.150057	0.276849
47	6	0	-8.189940	1.127230	4.150563
48	6	0	-8.501209	2.417192	4.587033
49	6	0	-9.043599	3.325233	3.686134
50	6	0	-9.271479	2.946112	2.356593
51	6	0	-8.429528	0.800534	2.823535
52	6	0	-9.851776	3.796156	1.312136
53	6	0	-10.235796	5.130741	1.528952

54	6	0	-10.782259	5.877727	0.488317
55	6	0	-10.945040	5.289761	-0.771490
56	6	0	-10.563609	3.961855	-0.990294
57	1	0	-7.770174	0.383547	4.818627
58	1	0	-8.209248	-0.187484	2.438145
59	1	0	-11.372811	5.868215	-1.587106
60	1	0	-10.703418	3.533812	-1.979948
61	6	0	-13.206034	-0.089777	1.505970
62	6	0	-13.883721	-0.418571	0.326188
63	6	0	-13.251109	-0.239140	-0.901540
64	6	0	-11.941571	0.268842	-0.951540
65	6	0	-11.902613	0.415965	1.459217
66	6	0	-11.208892	0.482602	-2.203734
67	6	0	-11.707338	0.250591	-3.492566
68	6	0	-10.913699	0.507080	-4.603798
69	6	0	-9.619106	0.998066	-4.418165
70	6	0	-9.169738	1.209531	-3.122611
71	1	0	-13.696015	-0.226930	2.467202
72	1	0	-11.403264	0.661819	2.393261
73	1	0	-8.965271	1.216702	-5.254967
74	1	0	-8.176075	1.593465	-2.926544
75	7	0	-8.949853	1.681296	1.945072
76	7	0	-9.935715	0.957247	-2.041969
77	6	0	9.210430	-0.614439	-2.912250
78	6	0	10.325198	1.455174	-2.992173
79	6	0	9.077117	-0.645812	-4.295132
80	1	0	8.835551	-1.412876	-2.283727
81	6	0	10.216444	1.470363	-4.386290
82	6	0	9.589760	0.415244	-5.042135
83	1	0	8.582424	-1.487991	-4.765349
84	6	0	10.955410	2.492749	-2.169012
85	6	0	11.518248	3.235275	0.085703
86	6	0	11.551453	3.644278	-2.694523
87	6	0	12.107509	4.379039	-0.446697
88	1	0	11.509123	3.093967	1.162179
89	6	0	12.124921	4.582944	-1.834940
90	1	0	12.555500	5.116816	0.212974
91	7	0	9.815941	0.406442	-2.280016
92	6	0	12.282598	-0.662002	1.209221
93	6	0	12.384638	-1.220813	-1.163707
94	6	0	13.461678	-1.383044	1.426883
95	6	0	13.555494	-1.939346	-0.935926
96	1	0	11.979832	-1.170162	-2.169876
97	6	0	14.094187	-2.019074	0.357252

98	1	0	14.055390	-2.439054	-1.760842
99	6	0	9.686153	1.403813	2.707888
100	6	0	11.548297	0.035222	2.270675
101	6	0	9.984591	1.451637	4.064353
102	1	0	8.831495	1.923629	2.292889
103	6	0	11.887984	0.048086	3.627176
104	6	0	11.103095	0.759471	4.529436
105	1	0	9.347394	2.020459	4.731879
106	7	0	10.444319	0.713526	1.837693
107	1	0	11.362877	0.773497	5.583043
108	1	0	12.759859	-0.496011	3.970321
109	1	0	13.894596	-1.454602	2.419684
110	1	0	15.006907	-2.580930	0.531357
111	1	0	11.572864	3.820595	-3.765334
112	1	0	10.621319	2.302358	-4.950159
113	1	0	9.503394	0.421801	-6.123936
114	1	0	-8.326049	2.709804	5.617663
115	1	0	-9.295524	4.329122	4.007580
116	1	0	-10.110433	5.589406	2.505813
117	1	0	-11.079489	6.908901	0.656517
118	1	0	-13.778482	-0.497304	-1.815730
119	1	0	-14.895938	-0.810829	0.364473
120	1	0	-12.716228	-0.125155	-3.617140
121	1	0	-11.299916	0.329607	-5.602720
122	77	0	10.055106	0.545640	-0.208700
123	77	0	-9.337499	1.265973	-0.065075
124	6	0	-10.006354	3.181245	0.037939
125	6	0	-11.234364	0.606170	0.236771
126	6	0	10.943195	2.259427	-0.756369
127	6	0	11.730030	-0.552803	-0.106908
128	1	0	12.582991	5.477309	-2.246370
129	6	0	-3.845529	-4.026885	-0.476923
130	6	0	-4.050642	-4.902229	-1.541036
131	6	0	-3.709707	-4.519601	0.823947
132	6	0	-4.114715	-6.277324	-1.301150
133	1	0	-4.157541	-4.515102	-2.549655
134	6	0	-3.777413	-5.892647	1.046958
135	1	0	-3.554239	-3.832278	1.650024
136	6	0	-3.979684	-6.804333	-0.007294
137	1	0	-4.274044	-6.935858	-2.146924
138	1	0	-3.670890	-6.256214	2.064321
139	6	0	-4.049245	-8.312076	0.289340
140	6	0	-5.224316	-8.587489	1.256294
141	1	0	-5.281752	-9.659333	1.481168

142	1	0	-5.105958	-8.052601	2.204717
143	1	0	-6.179212	-8.282664	0.811758
144	6	0	-4.263936	-9.149795	-0.983790
145	1	0	-3.446801	-9.018410	-1.702462
146	1	0	-4.304025	-10.212019	-0.717588
147	1	0	-5.205263	-8.898002	-1.485630
148	6	0	-2.727392	-8.765105	0.951621
149	1	0	-2.766838	-9.838766	1.171584
150	1	0	-1.873406	-8.586508	0.287234
151	1	0	-2.540933	-8.237791	1.893267
152	6	0	2.495523	1.141035	0.854568
153	6	0	2.248408	1.930953	1.975546
154	6	0	1.913939	1.467452	-0.373547
155	6	0	1.407714	3.042087	1.869010
156	1	0	2.705047	1.678086	2.927603
157	6	0	1.079318	2.578995	-0.465319
158	1	0	2.116003	0.855784	-1.247536
159	6	0	0.801732	3.390412	0.651768
160	1	0	1.231211	3.635582	2.758476
161	1	0	0.637769	2.814780	-1.428754
162	6	0	-0.134557	4.602442	0.503812
163	6	0	-1.524632	4.116972	0.028999
164	1	0	-2.202462	4.971560	-0.086430
165	1	0	-1.466345	3.602364	-0.936090
166	1	0	-1.969070	3.427220	0.756219
167	6	0	0.449697	5.577595	-0.544517
168	1	0	-0.212188	6.444336	-0.660480
169	1	0	1.435856	5.943466	-0.234633
170	1	0	0.556839	5.105479	-1.527002
171	6	0	-0.315806	5.367195	1.827199
172	1	0	-0.756415	4.735857	2.607425
173	1	0	0.634152	5.763972	2.203498
174	1	0	-0.989973	6.216209	1.667381
175	6	0	0.613466	-3.720955	-1.348728
176	6	0	0.981696	-2.378201	-1.660885
177	6	0	-0.181064	-1.566338	-1.562511
178	6	0	-1.292935	-2.407695	-1.198785
179	6	0	-0.781101	-3.747426	-1.057671
180	1	0	1.279471	-4.573350	-1.308695
181	1	0	1.979884	-2.028340	-1.891880
182	1	0	-0.231652	-0.499429	-1.724892
183	1	0	-1.337011	-4.625982	-0.769867
184	6	0	1.288024	-3.114151	1.901204
185	6	0	-0.100277	-3.205643	2.198254

186	6	0	-0.662609	-1.899465	2.081609
187	6	0	0.374102	-0.994952	1.711493
188	6	0	1.603172	-1.740134	1.606283
189	1	0	1.993658	-3.932624	1.889318
190	1	0	-0.638681	-4.113688	2.437231
191	1	0	-1.705596	-1.638668	2.209902
192	1	0	0.240738	0.060888	1.534982
193	26	0	0.183023	-2.473138	0.266425

1T$_1$-2

Electronic Energy *E*= -4847.35

Thermal correction to Gibbs Free Energy= 1.393353

Center Number	Atomic Number	Atomic Type	Coordinates (Angstroms) X	Y	Z
1	6	0	3.475899	-0.254986	-1.549844
2	6	0	7.604719	2.995472	1.041127
3	6	0	6.452598	2.445838	0.513795
4	6	0	6.518980	1.218510	-0.184187
5	6	0	7.799167	0.599485	-0.301550
6	6	0	8.825737	2.330070	0.876168
7	6	0	5.421560	0.516239	-0.782596
8	6	0	7.964392	-0.669937	-0.978875
9	6	0	6.852104	-1.343858	-1.538884
10	6	0	5.570512	-0.716096	-1.416451
11	6	0	7.070929	-2.577665	-2.183252
12	1	0	6.233898	-3.110850	-2.620587
13	6	0	8.354448	-3.084098	-2.249173
14	6	0	9.408509	-2.359312	-1.672637
15	1	0	7.578056	3.934882	1.581987
16	1	0	5.506766	2.955418	0.639318
17	1	0	9.746618	2.736247	1.278685
18	1	0	8.564518	-4.028713	-2.738692
19	1	0	10.427352	-2.728087	-1.710579
20	7	0	4.370551	-1.181214	-1.882390
21	7	0	4.066283	0.809539	-0.880628
22	7	0	9.223998	-1.191023	-1.055166
23	7	0	8.920872	1.169810	0.225225
24	6	0	9.653184	-1.826743	3.684450

25	6	0	10.579449	-2.866499	3.796037
26	6	0	11.531344	-3.036566	2.798327
27	6	0	11.555874	-2.172680	1.695324
28	6	0	9.714708	-1.000751	2.571274
29	6	0	12.511296	-2.229269	0.584476
30	6	0	13.525008	-3.198015	0.487605
31	6	0	14.394013	-3.192736	-0.600660
32	6	0	14.249660	-2.216983	-1.593835
33	6	0	13.243004	-1.250313	-1.498944
34	1	0	8.895375	-1.652978	4.440113
35	1	0	9.021217	-0.179091	2.440436
36	1	0	14.925957	-2.207802	-2.445539
37	1	0	13.157788	-0.505227	-2.286209
38	6	0	13.391049	2.450576	2.479452
39	6	0	13.821415	3.441515	1.589421
40	6	0	13.387610	3.411249	0.266327
41	6	0	12.523875	2.391305	-0.168621
42	6	0	12.531232	1.434449	2.049376
43	6	0	12.022657	2.292758	-1.543299
44	6	0	12.346006	3.159126	-2.596038
45	6	0	11.811284	2.949206	-3.861179
46	6	0	10.952245	1.867079	-4.067273
47	6	0	10.660459	1.038456	-2.993310
48	1	0	13.728212	2.468880	3.513275
49	1	0	12.215020	0.678986	2.764637
50	1	0	10.513941	1.661743	-5.037506
51	1	0	10.003908	0.183887	-3.103123
52	7	0	10.633596	-1.166593	1.598578
53	7	0	11.174100	1.242070	-1.763380
54	1	0	10.561065	-3.534430	4.651645
55	1	0	12.260610	-3.835079	2.870215
56	1	0	13.638712	-3.956544	1.257043
57	1	0	15.177413	-3.941545	-0.675146
58	1	0	13.721873	4.182350	-0.422161
59	1	0	14.489128	4.229815	1.925137
60	1	0	13.018327	3.990397	-2.418603
61	1	0	12.062749	3.618971	-4.677647
62	77	0	10.788968	0.034939	-0.102990
63	6	0	12.347531	-1.229500	-0.415119
64	6	0	12.072195	1.376767	0.721547
65	6	0	3.384987	1.939020	-0.306916
66	6	0	3.334529	3.152067	-0.991228
67	6	0	2.777235	1.815495	0.945225
68	6	0	2.671356	4.241808	-0.420264

69	1	0	3.809881	3.247436	-1.962606
70	6	0	2.117780	2.910066	1.500558
71	1	0	2.827641	0.872646	1.481837
72	6	0	2.047225	4.147911	0.833614
73	1	0	2.649084	5.172448	-0.975110
74	1	0	1.654441	2.792305	2.475170
75	6	0	1.307279	5.326964	1.488004
76	6	0	1.961265	5.649302	2.851800
77	1	0	1.439519	6.487797	3.328535
78	1	0	1.918649	4.796463	3.537685
79	1	0	3.013302	5.931859	2.726522
80	6	0	1.348099	6.597663	0.620623
81	1	0	0.868653	6.444508	-0.353105
82	1	0	0.810297	7.404085	1.131677
83	1	0	2.374483	6.940951	0.447120
84	6	0	-0.172848	4.938234	1.712151
85	1	0	-0.711189	5.766732	2.188039
86	1	0	-0.669002	4.714891	0.759863
87	1	0	-0.270163	4.060278	2.360309
88	6	0	-0.140015	0.038719	-2.446458
89	6	0	0.067269	-1.379132	-2.445571
90	6	0	1.415830	-1.623845	-2.103814
91	6	0	2.061946	-0.370122	-1.876436
92	6	0	1.085854	0.660964	-2.081072
93	1	0	-1.055749	0.553187	-2.711754
94	1	0	-0.678888	-2.130821	-2.672579
95	1	0	1.881693	-2.594552	-1.994880
96	1	0	1.255269	1.726543	-2.021142
97	6	0	-3.428568	-0.632735	1.695803
98	6	0	-8.249639	-3.282325	-0.391693
99	6	0	-6.972600	-2.916022	0.093928
100	6	0	-6.726957	-1.589338	0.439306
101	6	0	-7.789481	-0.618515	0.291397
102	6	0	-9.227469	-2.312012	-0.508740
103	6	0	-5.493132	-1.047497	0.948353
104	6	0	-7.627245	0.750676	0.620153
105	6	0	-6.386676	1.274783	1.111349
106	6	0	-5.318749	0.316035	1.263759
107	6	0	-6.276543	2.624883	1.408406
108	1	0	-5.339407	3.023887	1.782141
109	6	0	-7.397261	3.462589	1.218209
110	6	0	-8.575255	2.903725	0.745409
111	1	0	-8.467203	-4.307601	-0.669327
112	1	0	-6.201988	-3.668061	0.193502

113	1	0	-10.219433	-2.561251	-0.873025
114	1	0	-7.352442	4.523729	1.436771
115	1	0	-9.457058	3.517309	0.588468
116	7	0	-4.068051	0.557142	1.708202
117	7	0	-4.281013	-1.640458	1.231930
118	7	0	-8.717866	1.596109	0.450312
119	7	0	-9.036217	-1.015114	-0.185180
120	6	0	-11.231829	-0.742043	3.818949
121	6	0	-12.103003	0.234469	4.307950
122	6	0	-12.496280	1.271219	3.470735
123	6	0	-12.020243	1.333103	2.153769
124	6	0	-10.784715	-0.635795	2.509631
125	6	0	-12.367379	2.358880	1.164140
126	6	0	-13.223727	3.439125	1.441605
127	6	0	-13.510448	4.375639	0.451417
128	6	0	-12.941022	4.230798	-0.819568
129	6	0	-12.089309	3.156833	-1.097366
130	1	0	-10.901109	-1.572193	4.433249
131	1	0	-10.111073	-1.366816	2.079835
132	1	0	-13.162854	4.958182	-1.597386
133	1	0	-11.662798	3.073122	-2.094633
134	6	0	-14.003914	-1.947317	-1.163692
135	6	0	-13.961807	-1.995375	-2.562468
136	6	0	-12.965168	-1.299704	-3.242438
137	6	0	-12.009481	-0.556959	-2.526810
138	6	0	-13.053605	-1.210037	-0.450193
139	6	0	-10.934184	0.204324	-3.172067
140	6	0	-10.723719	0.315307	-4.553629
141	6	0	-9.673210	1.083121	-5.040747
142	6	0	-8.834634	1.740985	-4.137424
143	6	0	-9.081117	1.598178	-2.779388
144	1	0	-14.781886	-2.486523	-0.627635
145	1	0	-13.113300	-1.193513	0.635907
146	1	0	-8.004645	2.354083	-4.470792
147	1	0	-8.463765	2.088014	-2.036190
148	7	0	-11.159286	0.372298	1.696306
149	7	0	-10.096619	0.850027	-2.302855
150	1	0	-12.472792	0.186247	5.327519
151	1	0	-13.175634	2.034145	3.832131
152	1	0	-13.668093	3.555308	2.426560
153	1	0	-14.171982	5.210075	0.666510
154	1	0	-12.936199	-1.338965	-4.328061
155	1	0	-14.700320	-2.568407	-3.116051
156	1	0	-11.387140	-0.197873	-5.240045

157	1	0	-9.511158	1.170194	-6.110727
158	77	0	-10.523981	0.594092	-0.277602
159	6	0	-11.772710	2.194117	-0.120419
160	6	0	-12.029379	-0.498436	-1.103124
161	6	0	-3.926924	-3.018145	1.018633
162	6	0	-3.375895	-3.408480	-0.200448
163	6	0	-4.134233	-3.959289	2.029479
164	6	0	-3.027495	-4.746013	-0.405617
165	1	0	-3.224106	-2.675318	-0.987147
166	6	0	-3.781279	-5.288778	1.810173
167	1	0	-4.564604	-3.651613	2.977410
168	6	0	-3.219818	-5.715371	0.591095
169	1	0	-2.603288	-5.021387	-1.364149
170	1	0	-3.949073	-6.004750	2.608731
171	6	0	-2.848938	-7.195571	0.395370
172	6	0	-4.117076	-8.065656	0.558094
173	1	0	-3.864980	-9.124559	0.424458
174	1	0	-4.566211	-7.951477	1.550461
175	1	0	-4.874337	-7.800574	-0.189316
176	6	0	-2.250295	-7.470034	-0.995792
177	1	0	-1.328213	-6.901642	-1.164024
178	1	0	-2.004029	-8.534320	-1.082010
179	1	0	-2.954305	-7.226657	-1.799861
180	6	0	-1.807689	-7.607905	1.461655
181	1	0	-1.538869	-8.663507	1.334557
182	1	0	-0.892040	-7.011591	1.368865
183	1	0	-2.192897	-7.481517	2.479119
184	6	0	-0.045251	-1.553955	2.793256
185	6	0	-0.055339	-0.097236	2.942897
186	6	0	-1.280551	0.368097	2.542439
187	6	0	-2.084616	-0.779346	2.125642
188	6	0	-1.264265	-1.960693	2.294602
189	1	0	0.783908	-2.201817	3.055803
190	1	0	0.765448	0.495246	3.330402
191	1	0	-1.627935	1.392063	2.547153
192	1	0	-1.563226	-2.980205	2.104642
193	26	0	0.146318	-0.478156	-0.273009

$1T_1$-3

Electronic Energy *E*= -4847.4099

Thermal correction to Gibbs Free Energy= 1.398094

Center Atomic Atomic Coordinates (Angstroms)

Number	Number	Type	X	Y	Z
1	6	0	3.117127	0.206442	1.337524
2	6	0	-3.100644	-0.561652	-1.350960
3	6	0	-7.937405	-3.362341	0.408552
4	6	0	-6.673720	-2.966976	0.015980
5	6	0	-6.447318	-1.623809	-0.363806
6	6	0	-7.563466	-0.735323	-0.326927
7	6	0	-8.979995	-2.427187	0.426358
8	6	0	-5.201151	-1.053151	-0.782181
9	6	0	-7.433002	0.655759	-0.707703
10	6	0	-6.186757	1.179856	-1.129394
11	6	0	-5.068112	0.285146	-1.150131
12	6	0	-6.121194	2.538643	-1.496456
13	1	0	-5.177553	2.961648	-1.823258
14	6	0	-7.266140	3.309072	-1.434231
15	6	0	-8.465520	2.721436	-1.004361
16	1	0	-8.134447	-4.386719	0.704704
17	1	0	-5.865092	-3.685449	-0.000141
18	1	0	-9.981078	-2.705674	0.735384
19	1	0	-7.257024	4.357717	-1.710153
20	1	0	-9.381536	3.298485	-0.945294
21	6	0	7.729201	3.368267	-0.409044
22	6	0	6.498285	2.876444	-0.021555
23	6	0	6.375602	1.520712	0.360839
24	6	0	7.557468	0.721460	0.331157
25	6	0	8.841305	2.517034	-0.419720
26	6	0	5.176250	0.856753	0.777593
27	6	0	7.535399	-0.672469	0.722721
28	6	0	6.331355	-1.290544	1.140549
29	6	0	5.145645	-0.486232	1.150566
30	6	0	6.369749	-2.649245	1.511456
31	1	0	5.460342	-3.143394	1.835615
32	6	0	7.570888	-3.329221	1.454240
33	6	0	8.722799	-2.652075	1.026211
34	1	0	7.847795	4.404597	-0.705435
35	1	0	5.636505	3.529843	-0.011071
36	1	0	9.820134	2.873690	-0.719621
37	1	0	7.641532	-4.375338	1.731027
38	1	0	9.679039	-3.159344	0.964715
39	7	0	-3.779504	0.575640	-1.500272
40	7	0	-3.927774	-1.592408	-0.920021
41	7	0	3.864711	1.296905	0.907188
42	7	0	3.881139	-0.874483	1.494655

43	7	0	-8.550023	1.438064	-0.649667
44	7	0	-8.799885	-1.155131	0.068113
45	7	0	8.758442	1.234924	-0.061626
46	7	0	8.710376	-1.365786	0.671856
47	6	0	-8.899416	1.072090	4.124332
48	6	0	-9.571735	2.238400	4.497437
49	6	0	-10.444418	2.835942	3.596313
50	6	0	-10.641323	2.271727	2.328918
51	6	0	-9.125812	0.552883	2.857697
52	6	0	-11.541065	2.787595	1.292226
53	6	0	-12.304953	3.957366	1.445892
54	6	0	-13.139298	4.386428	0.416581
55	6	0	-13.210331	3.644855	-0.768502
56	6	0	-12.450550	2.480758	-0.924669
57	1	0	-8.213075	0.568477	4.795822
58	1	0	-8.633621	-0.352026	2.522992
59	1	0	-13.861568	3.974716	-1.574682
60	1	0	-12.527347	1.926857	-1.857190
61	6	0	-13.479574	-1.877445	2.150059
62	6	0	-14.106822	-2.531247	1.083082
63	6	0	-13.657377	-2.311141	-0.216595
64	6	0	-12.581296	-1.437766	-0.451634
65	6	0	-12.408446	-1.007978	1.919224
66	6	0	-12.050177	-1.155014	-1.789157
67	6	0	-12.538086	-1.682145	-2.992348
68	6	0	-11.951467	-1.323554	-4.199757
69	6	0	-10.875250	-0.433010	-4.196990
70	6	0	-10.426082	0.059720	-2.980206
71	1	0	-13.827731	-2.044464	3.166801
72	1	0	-11.942807	-0.514841	2.769069
73	1	0	-10.388480	-0.121494	-5.114373
74	1	0	-9.597755	0.755586	-2.925068
75	7	0	-9.966909	1.133974	1.978674
76	7	0	-10.989560	-0.290715	-1.806495
77	6	0	9.203130	-1.396905	-2.575992
78	6	0	10.917572	0.173238	-2.929706
79	6	0	9.025963	-1.587397	-3.938852
80	1	0	8.609907	-1.939083	-1.849783
81	6	0	10.773757	0.018730	-4.314941
82	6	0	9.826795	-0.861520	-4.823823
83	1	0	8.277513	-2.289089	-4.289564
84	6	0	11.884098	1.051124	-2.262575
85	6	0	12.773148	1.846361	-0.162388
86	6	0	12.783293	1.877208	-2.958668

87	6	0	13.667318	2.667245	-0.857724
88	1	0	12.792552	1.855116	0.924757
89	6	0	13.674707	2.685327	-2.257350
90	1	0	14.362634	3.295021	-0.305187
91	7	0	10.117048	-0.539018	-2.078884
92	6	0	12.428313	-1.728228	1.470313
93	6	0	12.291200	-2.577127	-0.787149
94	6	0	13.393295	-2.702160	1.780028
95	6	0	13.252162	-3.544821	-0.476154
96	1	0	11.881196	-2.551665	-1.793812
97	6	0	13.805761	-3.610075	0.807858
98	1	0	13.572329	-4.250148	-1.239581
99	6	0	10.495403	1.090318	2.734464
100	6	0	11.945942	-0.736817	2.437162
101	6	0	10.894414	1.258706	4.052623
102	1	0	9.758922	1.742213	2.280906
103	6	0	12.377371	-0.610556	3.764308
104	6	0	11.852143	0.387025	4.576438
105	1	0	10.460460	2.055195	4.646629
106	7	0	10.998162	0.119058	1.945958
107	1	0	12.187058	0.486178	5.604284
108	1	0	13.125013	-1.292528	4.152137
109	1	0	13.824145	-2.755236	2.776078
110	1	0	14.552380	-4.361851	1.047187
111	1	0	12.790497	1.892969	-4.045012
112	1	0	11.407250	0.586239	-4.986557
113	1	0	9.715862	-0.983008	-5.896894
114	1	0	-9.419841	2.674115	5.480101
115	1	0	-10.980260	3.737005	3.871142
116	1	0	-12.250767	4.535642	2.364061
117	1	0	-13.729661	5.290394	0.535931
118	1	0	-14.145676	-2.821028	-1.042498
119	1	0	-14.938663	-3.205956	1.264051
120	1	0	-13.377087	-2.367918	-2.976159
121	1	0	-12.329983	-1.731081	-5.131984
122	77	0	10.443334	-0.200346	-0.042467
123	77	0	-10.365725	0.406796	0.061216
124	6	0	-11.595814	2.022386	0.093353
125	6	0	-11.928042	-0.766228	0.619968
126	6	0	11.858027	1.021172	-0.839833
127	6	0	11.851316	-1.647469	0.171659
128	1	0	14.369971	3.323215	-2.795562
129	6	0	-3.535790	-2.942650	-0.618239
130	6	0	-3.532762	-3.909630	-1.621260

131	6	0	-3.162418	-3.282006	0.685162
132	6	0	-3.146003	-5.218057	-1.319285
133	1	0	-3.826491	-3.643501	-2.632006
134	6	0	-2.780818	-4.590565	0.971122
135	1	0	-3.174027	-2.527550	1.465991
136	6	0	-2.760016	-5.589176	-0.021814
137	1	0	-3.151493	-5.948956	-2.119378
138	1	0	-2.495188	-4.833350	1.989992
139	6	0	-2.327491	-7.020324	0.340805
140	6	0	-3.270439	-7.579563	1.431590
141	1	0	-2.969983	-8.599576	1.699930
142	1	0	-3.245196	-6.973280	2.343395
143	1	0	-4.307002	-7.614219	1.075689
144	6	0	-2.372559	-7.969529	-0.869989
145	1	0	-1.699880	-7.642750	-1.671329
146	1	0	-2.055601	-8.971241	-0.558637
147	1	0	-3.383417	-8.055392	-1.284924
148	6	0	-0.879697	-6.994258	0.883897
149	1	0	-0.560769	-8.008915	1.151343
150	1	0	-0.183644	-6.607988	0.129615
151	1	0	-0.791081	-6.369317	1.778984
152	6	0	3.371856	2.609840	0.588343
153	6	0	3.303164	3.590538	1.575840
154	6	0	2.969466	2.900124	-0.718228
155	6	0	2.822260	4.862739	1.254692
156	1	0	3.619533	3.363076	2.589191
157	6	0	2.493979	4.173032	-1.023377
158	1	0	3.032828	2.135645	-1.486692
159	6	0	2.405824	5.183817	-0.046798
160	1	0	2.778628	5.605359	2.042808
161	1	0	2.188186	4.378286	-2.044632
162	6	0	1.871663	6.574080	-0.431994
163	6	0	0.427518	6.436109	-0.967903
164	1	0	0.036305	7.420992	-1.250241
165	1	0	0.380479	5.791978	-1.852570
166	1	0	-0.236716	6.013842	-0.204288
167	6	0	2.769074	7.178588	-1.536913
168	1	0	2.395317	8.168988	-1.823483
169	1	0	3.801353	7.294464	-1.185728
170	1	0	2.785138	6.554402	-2.436739
171	6	0	1.853808	7.545446	0.761715
172	1	0	1.207714	7.186787	1.571266
173	1	0	2.857395	7.708534	1.171185
174	1	0	1.467171	8.517079	0.434253

175	6	0	0.541054	-1.323011	-1.830433
176	6	0	0.479273	0.067669	-2.112721
177	6	0	-0.868947	0.483835	-1.917686
178	6	0	-1.675975	-0.660834	-1.612880
179	6	0	-0.775167	-1.761304	-1.480766
180	1	0	1.416823	-1.957526	-1.882934
181	1	0	1.300826	0.706914	-2.409505
182	1	0	-1.242355	1.491290	-2.041918
183	1	0	-1.028848	-2.777186	-1.218383
184	6	0	0.961385	-1.003601	1.873886
185	6	0	-0.412359	-0.680317	2.085248
186	6	0	-0.564286	0.711569	1.848088
187	6	0	0.715363	1.240439	1.496520
188	6	0	1.688544	0.198341	1.596302
189	1	0	1.399577	-1.987412	1.973254
190	1	0	-1.188583	-1.378188	2.371638
191	1	0	-1.479492	1.285815	1.918391
192	1	0	0.901412	2.276659	1.257894
193	26	0	0.013304	-0.272001	-0.006027

2T$_1$

Electronic Energy *E*= -2361.7727

Thermal correction to Gibbs Free Energy= 0..680335

C	-4.33732410	-2.45395480	-0.39837470
C	-1.16113866	2.43481235	0.40900011
C	-2.15512139	1.44124019	0.24753386
C	-1.77280902	0.12162750	0.01846422
C	-0.36329750	-0.19656479	-0.04968399
C	0.17069214	2.07170348	0.33467940
C	-2.64765161	-1.00858021	-0.16586072
C	0.11393659	-1.51160381	-0.27541407
C	-0.77328902	-2.62230333	-0.46298405
C	-2.18240549	-2.31704690	-0.39751737
C	-0.25465876	-3.88865226	-0.68925312
H	-0.92336151	-4.73106916	-0.83184251
C	1.14635687	-4.05929038	-0.73056908
C	1.95979811	-2.95192287	-0.54027443
H	-1.43162443	3.46945502	0.58834972
H	-3.19968372	1.71519781	0.30337260
H	0.95765368	2.80960234	0.45736777
H	1.59159381	-5.03258245	-0.90500011

H	3.04110302	-3.04621930	-0.56597631
N	-4.02388969	-1.11008373	-0.16799768
N	-3.20966437	-3.18324621	-0.53748180
N	0.58847387	0.80703682	0.11294365
N	1.49042517	-1.70831282	-0.31714122
C	2.27323560	-0.10443048	-3.02726967
C	3.26628722	1.89049858	-2.27659140
C	2.33799755	0.30098503	-4.35278448
H	1.86006593	-1.06591112	-2.74759224
C	3.35196621	2.34527543	-3.59982228
C	2.88771831	1.55244877	-4.64213094
H	1.96504689	-0.35332585	-5.13294626
C	3.72697994	2.61836930	-1.08928442
C	3.98676050	2.60602319	1.31068686
C	4.29825095	3.90178236	-1.14620021
C	4.55464239	3.88277014	1.25235570
H	3.87800109	2.12664363	2.28123015
C	4.71218358	4.53486979	0.02320930
H	4.87704849	4.37211710	2.16885610
N	2.71934681	0.66357534	-2.01282518
C	4.90652865	-1.26688751	1.28753733
C	5.38014514	-0.97945848	-1.06235907
C	6.13290327	-1.93402693	1.45410835
C	6.60032533	-1.64177770	-0.89432427
H	5.11388482	-0.61929838	-2.05373298
C	6.98036444	-2.12173168	0.36490332
H	7.25905114	-1.78386952	-1.74828875
C	1.88968206	-0.07370423	2.94866522
C	3.96572732	-1.02581918	2.38730264
C	2.03495566	-0.41604385	4.28552047
H	1.01487749	0.45457899	2.58960284
C	4.15694731	-1.39565744	3.72601230
C	3.19196134	-1.09309352	4.67886708
H	1.25655630	-0.15538067	4.99397863
N	2.82096899	-0.37094865	2.02021155
H	3.34069931	-1.37884513	5.71565818
H	5.06250041	-1.91597736	4.01586471
H	6.43177322	-2.30824171	2.42936974
H	7.92815937	-2.63649122	0.49497592
H	4.42095528	4.41038995	-2.09868219
H	3.78526571	3.31731955	-3.80544362
H	2.95516346	1.90393284	-5.66709458
Ir	2.66495053	0.11194906	-0.00264876
C	3.55396806	1.93692311	0.15011482

C	4.49674635	-0.77462018	0.01446643
H	5.15336304	5.52662596	-0.02126855
C	-4.96322755	-0.04107405	0.03695224
C	-5.40090900	0.26185817	1.32451911
C	-5.43598324	0.68976262	-1.05553722
C	-6.31510355	1.30048052	1.51782871
H	-5.03191807	-0.30915344	2.17095124
C	-6.34675899	1.72247087	-0.84709364
H	-5.09318120	0.44999137	-2.05732607
C	-6.80864018	2.05363760	0.44122505
H	-6.63852409	1.51363173	2.53000858
H	-6.70238853	2.27840009	-1.70906989
C	-7.81499508	3.20336729	0.62056366
C	-9.09946506	2.88783776	-0.18057960
H	-9.82322544	3.70395677	-0.06681477
H	-8.89448730	2.76927441	-1.24996617
H	-9.57008493	1.96487271	0.17868594
C	-7.19252362	4.51546039	0.08910979
H	-7.90247390	5.34286032	0.20843693
H	-6.27988067	4.76982363	0.64118281
H	-6.93708960	4.44510334	-0.97365803
C	-8.20434100	3.41588941	2.09441978
H	-8.67924546	2.52649110	2.52446093
H	-7.33735858	3.67430650	2.71331901
H	-8.92082567	4.24198436	2.16604002
C	-5.83018499	-4.45175865	-0.73552394
C	-7.17341497	-4.69203356	-0.76624222
C	-7.87225248	-3.42594278	-0.53549456
C	-6.94391810	-2.42588789	-0.36687906
C	-5.63338694	-3.02477748	-0.48522929
H	-5.02880827	-5.16510853	-0.86945838
H	-7.65622074	-5.64854326	-0.93138556
H	-8.94972652	-3.30639730	-0.50444412
H	-7.15903363	-1.38506084	-0.18033894

$3P_1$

Electronic Energy *E*= -3406.3408

Thermal correction to Gibbs Free Energy= 0.682043

Center Number	Atomic Number	Atomic Type	Coordinates (Angstroms) X	Y	Z
1	6	0	-3.704620	-1.160024	-1.338481
2	6	0	0.101106	2.761961	0.782515
3	6	0	-0.992455	2.048105	0.333604

4	6	0	-0.811423	0.750551	-0.199357
5	6	0	0.522196	0.242418	-0.245894
6	6	0	1.378420	2.192223	0.704844
7	6	0	-1.835519	-0.119048	-0.695170
8	6	0	0.808299	-1.075689	-0.771391
9	6	0	-0.232655	-1.905750	-1.255103
10	6	0	-1.568488	-1.392080	-1.197086
11	6	0	0.105078	-3.177886	-1.758749
12	1	0	-0.675132	-3.830891	-2.134378
13	6	0	1.429564	-3.569848	-1.766246
14	6	0	2.406958	-2.694093	-1.269528
15	1	0	-0.014215	3.757873	1.195797
16	1	0	-1.980695	2.483787	0.392819
17	1	0	2.255457	2.725934	1.052894
18	1	0	1.729453	-4.539837	-2.147279
19	1	0	3.454947	-2.971892	-1.262436
20	7	0	-2.716496	-2.019590	-1.590827
21	7	0	-3.214874	0.023457	-0.795291
22	7	0	2.110516	-1.486757	-0.785581
23	7	0	1.582763	0.972678	0.205282
24	6	0	2.474689	-1.532029	3.995893
25	6	0	3.475830	-2.475787	4.237530
26	6	0	4.461468	-2.679162	3.279521
27	6	0	4.444276	-1.944908	2.086149
28	6	0	2.497754	-0.833244	2.797399
29	6	0	5.427967	-2.047644	1.003262
30	6	0	6.518279	-2.934080	1.032954
31	6	0	7.413114	-2.976222	-0.033390
32	6	0	7.218585	-2.129628	-1.131008
33	6	0	6.134320	-1.246476	-1.163473
34	1	0	1.687987	-1.336141	4.715760
35	1	0	1.746087	-0.088957	2.564681
36	1	0	7.915626	-2.156983	-1.965449
37	1	0	6.010863	-0.601414	-2.030030
38	6	0	5.875007	2.884579	2.350127
39	6	0	6.242351	3.798756	1.355836
40	6	0	5.841700	3.579828	0.040062
41	6	0	5.073941	2.447674	-0.283693
42	6	0	5.112414	1.756306	2.030767
43	6	0	4.612845	2.148604	-1.643674
44	6	0	4.880221	2.914428	-2.786318
45	6	0	4.397438	2.510960	-4.025224
46	6	0	3.646275	1.336646	-4.115008
47	6	0	3.403520	0.615268	-2.954963

48	1	0	6.186156	3.050338	3.379013
49	1	0	4.844734	1.064041	2.825358
50	1	0	3.253227	0.980170	-5.060481
51	1	0	2.829671	-0.303206	-2.971593
52	7	0	3.448712	-1.033123	1.862695
53	7	0	3.865939	1.007073	-1.750632
54	1	0	3.488684	-3.044077	5.162358
55	1	0	5.248460	-3.404052	3.451922
56	1	0	6.672214	-3.591931	1.883706
57	1	0	8.255844	-3.661238	-0.009689
58	1	0	6.129291	4.291212	-0.729099
59	1	0	6.835986	4.673594	1.605299
60	1	0	5.468246	3.820481	-2.699622
61	1	0	4.606609	3.102858	-4.910915
62	77	0	3.545093	-0.027597	0.034448
63	6	0	5.210447	-1.181899	-0.105400
64	6	0	4.689451	1.506800	0.713129
65	6	0	-3.983564	1.167487	-0.386956
66	6	0	-4.215565	2.207523	-1.285006
67	6	0	-4.490480	1.238578	0.913339
68	6	0	-4.961281	3.318080	-0.881284
69	1	0	-3.819360	2.150692	-2.294244
70	6	0	-5.232635	2.351620	1.301462
71	1	0	-4.303629	0.428565	1.611713
72	6	0	-5.487297	3.416887	0.416284
73	1	0	-5.126481	4.111020	-1.601315
74	1	0	-5.617349	2.386613	2.316078
75	6	0	-6.311294	4.626392	0.890616
76	6	0	-5.600919	5.289709	2.093496
77	1	0	-6.181237	6.152150	2.443069
78	1	0	-5.491013	4.597578	2.935209
79	1	0	-4.601861	5.644570	1.813496
80	6	0	-6.483547	5.685710	-0.212689
81	1	0	-7.008371	5.284031	-1.087099
82	1	0	-7.076388	6.521803	0.175030
83	1	0	-5.520499	6.088750	-0.546731
84	6	0	-7.715189	4.148154	1.328529
85	1	0	-8.312181	5.001150	1.673246
86	1	0	-8.247915	3.676794	0.493896
87	1	0	-7.661675	3.424252	2.148744
88	6	0	-7.385498	-1.437211	-1.876279
89	6	0	-6.921724	-2.754555	-2.185456
90	6	0	-5.525268	-2.768488	-2.017826
91	6	0	-5.097567	-1.465227	-1.607453

92	6	0	-6.267781	-0.643902	-1.513878
93	1	0	-8.409197	-1.088720	-1.940616
94	1	0	-7.535030	-3.589104	-2.503304
95	1	0	-4.874476	-3.622351	-2.150800
96	1	0	-6.307584	0.404329	-1.259279
97	26	0	-6.771425	-2.292807	0.184429
98	17	0	-8.870605	-2.490547	1.238155
99	17	0	-5.139908	-2.997815	1.709441

Cl^-

Electronic Energy *E*=-460.300710433

Thermal correction to Gibbs Free Energy= -0.015023

Center Number	Atomic Number	Atomic Type	Coordinates (Angstroms) X	Y	Z
1	17	0	0.000000	0.000000	0.000000
